# Supplementary material for: Effects of a non-standard information leaflet on patient recruitment in acute care: Embedded cluster-randomised controlled trial
Source: PLoS One. 2025 Aug 1;20(8):e0327634. doi: 10.1371/journal.pone.0327634 (PMC12316219; doi:10.1371/journal.pone.0327634)
Supplement: S1 Table — Consolidated Standards for Reporting Trials (CONSORT) for recruitment SWATs and cRCT. (DOCX) [file pone.0327634.s001.docx]

**S1 CONSORT Checklist**

**Checklist of items for reporting embedded recruitment trials** (Madurasinghe et al., 2016)

| No. | Item description | Page in manuscript where item is located |
| --- | --- | --- |
| **Title and abstract** | | |
| 1a | Identification as an embedded randomised recruitment trial in the title | p. 1 |
| 1b | Structured summary of embedded recruitment trial design, methods, results, and conclusions (for specific guidance see CONSORT for abstracts) | p. 2 |
| **Introduction** – Background and objectives | | |
| 2a | Scientific background and explanation of rationale for the embedded recruitment trial including a brief description of the host trial(s) as appropriate | p. 3, 4 |
| 2b | Specific objectives or hypotheses for the embedded recruitment trial | p. 4 |
| **Methods** – Trial design | | |
| 3a | Description of embedded recruitment trial design (such as parallel, factorial, cluster) including allocation ratio | p. 4, 8 |
| 3b | Important changes to methods of the embedded recruitment trial after commencement (such as eligibility criteria), with reasons | n.a. |
| Participants | | |
| 4a | Eligibility criteria for participants for the embedded recruitment trial, including any differences from those for the host trial(s) | p. 5 |
| 4b | Settings and locations where the embedded recruitment trial was carried out, including a brief description of the host trial(s) as appropriate | p. 4, 5 |
| Interventions | | |
| 5 | The interventions for each group (including control group) within the embedded recruitment trial with sufficient details to allow replication, including how, where and when they were actually administered | p. 6 |
| Outcomes | | |
| 6a | Completely defined pre-specified primary and secondary outcome measures for the embedded recruitment trial, including how and when they were assessed | p. 7 |
| 6b | Any changes to embedded recruitment trial outcomes after the embedded recruitment trial commenced, with reasons | n.a. |
| Sample size | | |
| 7a | How sample size for the embedded recruitment trial was determined | p. 8 |
| 7b | When applicable, explanation of any interim analyses and stopping guidelines for the embedded recruitment trial | p. 15, 16 |
| Randomisation – Sequence generation | | |
| 8a | Method used to generate the random allocation sequence for the embedded recruitment trial | p. 5 |
| 8b | Type of randomisation; details of any restriction (such as blocking and block size) in the embedded recruitment trial | p. 5 |
| Allocation concealment mechanism | | |
| 9 | Mechanism used in the embedded recruitment trial to  implement the random allocation sequence (such as  sequentially numbered containers), describing any steps taken to conceal the sequence until interventions were assigned | p. 8 |
| Implementation | | |
| 10 | Who generated the random allocation sequence(s), who enrolled participants, and who assigned participants to embedded recruitment interventions? | p. 8 |
| Blinding | | |
| 11a | If done, who was blinded after assignment to embedded recruitment interventions (for example, participants, care providers, those assessing outcomes) and how? | p. 8 |
| 11b | If relevant, description of the similarity of interventions in the embedded recruitment trial | n.a. |
| Statistical methods | | |
| 12a | Statistical methods used to compare groups for primary and secondary outcomes of the embedded recruitment trial | p. 9 |
| 12b | Methods for additional analyses, such as subgroup analyses and adjusted analyses for the embedded recruitment trial | n.a. |
| **Results** – Participant flow (a diagram is strongly recommended) | | |
| 13a | For each group in the embedded recruitment trial, the  numbers of participants who were randomly assigned, received intended treatment, and were analysed for the primary outcome | p. 10 |
| 13b | For each group, losses and exclusions after randomisation to the embedded recruitment trial, together with reasons | p. 10 |
| Recruitment | | |
| 14a | Dates defining the periods of recruitment and follow-up for both embedded recruitment trial and host trial(s) | p. 9 |
| 14b | Why the embedded recruitment trial ended or was stopped | p. 15 |
| Baseline data | | |
| 15 | If possible a table showing baseline characteristics of each arm of the embedded recruitment trial | p. 10, 11 |
| Numbers analysed | | |
| 16 | For each group in the embedded recruitment trial, number of participants (denominator) included in each analysis and whether the analysis was by original assigned groups | p. 10 - 12 |
| Outcomes and estimation | | |
| 17a | For each primary and secondary outcome, results for each group in the embedded recruitment trial, and the estimated effect size and its precision (such as 95 % confidence interval) | p. 11 - 13 |
| 17b | For binary outcomes in the embedded recruitment trial, presentation of both absolute and relative effect sizes is recommended | p. 11 - 12 |
| Ancillary analyses | | |
| 18 | Results of any other analyses performed for the embedded recruitment trial, including subgroup analyses and adjusted analyses, distinguishing pre-specified from exploratory | n.a. |
| Harms | | |
| 19 | All important harms or unintended effects in each group for both the embedded recruitment trial and host trial(s) (for specific guidance see CONSORT for harms) | n.a. |
| **Discussion** - Limitations | | |
| 20 | Embedded recruitment trial limitations, addressing sources of potential bias, imprecision, and, if relevant, multiplicity of analyses | p. 15, 16 |
| Generalisability | | |
| 21 | Generalisability (external validity, applicability) of the embedded recruitment trial findings | p. 14, 15 |
| Interpretation |  |  |
| 22 | Interpretation consistent with results of the embedded  recruitment trial, balancing benefits and harms, and  considering other relevant evidence | p. 13, 14, 15 |
| **Other information** - Registration | | |
| 23 | Registration number and name of trial registry  (for all host trials and embedded recruitment trial if available) | p. 2 |
| Protocol | | |
| 24 | Where the embedded recruitment trial protocol can be accessed, if available | n.a. |
| Funding | | |
| 25 | For the embedded recruitment trial, sources of funding and other support, role of funders and collaborators | p. 15, 23 |

**CONSORT 2010 checklist of information to include when reporting a cluster randomised trial** (Campbell et al., 2012)

| No. | Item description | Page in manuscript where item is located |
| --- | --- | --- |
| **Title and abstract** | | |
| 1a | Identification as a cluster randomised trial in the title | p. 1 |
| 1b | Extension of CONSORT for abstracts to reports of cluster randomised trials | p. 2 |
| **Introduction** – Background and objectives | | |
| 2a | Rationale for using a cluster design | p. 3, 4 |
| 2b | Whether objectives pertain to the cluster level, the individual participant level, or both | p. 4, 8 |
| **Methods** – Trial design | | |
| 3a | Definition of cluster and description of how the design features apply to the clusters | p. 4, 8 |
| 3b | Important changes to methods after trial commencement (such as eligibility criteria), with reasons | n.a. |
| Participants | | |
| 4a | Eligibility criteria for clusters | p. 5 |
| 4b | Settings and locations where the data were collected | p. 5 |
| Interventions | | |
| 5 | Whether interventions pertain to the cluster level, the individual participant level, or both | p. 6 |
| Outcomes | | |
| 6a | Whether outcome measures pertain to the cluster level, the individual participant level, or both | p. 7 |
| 6b | Any changes to trial outcomes after the trial commenced, with reasons | n.a. |
| Sample size | | |
| 7a | Method of calculation, number of clusters(s) (and whether equal or unequal cluster sizes are assumed), cluster size, a coefficient of intracluster correlation (ICC or k), and an indication of its uncertainty | p. 8 |
| 7b | When applicable, explanation of any interim analyses and stopping guidelines | p. 15, 16 |
| **Randomisation** – Sequence generation | | |
| 8a | Method used to generate the random allocation sequence | p. 5 |
| 8b | Details of stratification or matching if used | p. 5 |
| Allocation concealment mechanism | | |
| 9 | Specification that allocation was based on clusters rather than individuals and whether allocation concealment (if any) was at the cluster level, the individual participant level, or both | p. 8 |
| Implementation | | |
| 10a | Who generated the random allocation sequence, who enrolled clusters, and who assigned clusters to interventions | p. 8 |
| 10b | Mechanism by which individual participants were included in clusters for the purposes of the trial (such as complete enumeration, random sampling) | p. 8 |
| 10c | From whom consent was sought (representatives of the cluster, or individual cluster members, or both) and whether consent was sought before or after randomisation | p. 4 |
| Blinding | | |
| 11a | If done, who was blinded after assignment to interventions (for example, participants, care providers, those assessing outcomes) and how | p. 8 |
| 11b | If relevant, description of the similarity of interventions | n.a. |
| Statistical methods | | |
| 12a | How clustering was taken into account | p. 9 |
| 12b | Methods for additional analyses, such as subgroup analyses and adjusted analyses | n.a. |
| **Results** – Participant flow (a diagram is strongly recommended) | | |
| 13a | For each group, the numbers of clusters that were randomly assigned, received intended treatment, and were analysed for the primary outcome | p. 10 |
| 13b | For each group, losses and exclusions for both clusters and individual cluster members | p. 10 |
| Recruitment | | |
| 14a | Dates defining the periods of recruitment and follow-up | p. 9 |
| 14b | Why the trial ended or was stopped | p. 15 |
| Baseline data | | |
| 15 | Baseline characteristics for the individual and cluster levels as applicable for each group | p. 10, 11 |
| Numbers analysed | | |
| 16 | For each group, number of clusters included in each analysis | p. 10 - 12 |
| Outcomes and estimation | | |
| 17a | Results at the individual or cluster level as applicable and a coefficient of intracluster correlation (ICC or k) for each primary outcome | p. 11 - 13 |
| 17b | For binary outcomes, presentation of both absolute and relative effect sizes is recommended | p. 11 - 12 |
| Ancillary analyses | | |
| 18 | Results of any other analyses performed, including subgroup analyses and adjusted analyses, distinguishing prespecified from exploratory | n.a. |
| Harms | | |
| 19 | All important harms or unintended effects in each group (for specific guidance see CONSORT for harms106) | n.a. |
| **Discussion** - Limitations | | |
| 20 | Trial limitations, addressing sources of potential bias, imprecision, and, if relevant, multiplicity of analyses | p. 15, 16 |
| Generalisability | | |
| 21 | Generalisability to clusters and/or individual participants (as relevant) | p. 14, 15 |
| Interpretation |  |  |
| 22 | Interpretation consistent with results, balancing benefits and harms, and considering other relevant evidence | p. 13, 14, 15 |
| **Other information** - Registration | | |
| 23 | Registration number and name of trial registry | p. 2 |
| Protocol | | |
| 24 | Where the full trial protocol can be accessed, if available | n.a. |
| Funding | | |
| 25 | Sources of funding and other support (such as supply of drugs), role of funders | p. 15, 23 |
