# Supplementary material for: Effects of a non-standard information leaflet on patient recruitment in acute care: Embedded cluster-randomised controlled trial
Source: PLoS One. 2025 Aug 1;20(8):e0327634. doi: 10.1371/journal.pone.0327634 (PMC12316219; doi:10.1371/journal.pone.0327634)
Supplement: S2 File — (PDF) [file pone.0327634.s002.pdf]

Sleep Acute

Scientific study to analyse patients' sleep during inpatient hospital care

Responsible according to §4 Abs. 7 DS-GVO:

Institute for Nursing Science, Faculty of Medicine, University of Cologne
Prof. Dr phil. Sascha Köpke
Gleueler Straße 176-178
50935 Cologne
Phone: 0221 478 51658
E-mail: sascha.koepke@uk-koeln.de

Information letter for patients

Dear Patient,

this letter provides information about the objectives and procedures of the study mentioned above. The study is being conducted by the Institute of Nursing Science, University of Cologne. **Your participation in the study is voluntary**.

Please read the following text carefully. If you have any questions, feel free to speak with your ward supervisor or contact us directly. You will find our contact details at the end of this letter.

I. Information about the study

Background and Objectives

Sleep is a state essential for health, well-being and daily functioning. While illness often increases the need for rest and sleep, previous scientific studies suggest that sleep problems are common during hospital stays and are associated with various risks.

The goal of the "Sleep Acute" study is to learn more about patients' sleep and potential sleep issues during their hospital stay. Additionally, the study aims to identify strategies for promoting better sleep.

At the same time, we are analysing the acceptability and effectiveness of the written informational materials used in this study. This will help us to gain valuable insights into designing informational materials for future scientific studies.

Potential Participants

You can participate in the study if you are an adult and have spent at least two nights in the hospital on a general ward (i.e., not a monitoring or intensive care unit) at the time of data collection.

Procedures

If you decide to participate in the study, you will receive two questionnaires along with this written study information. One questionnaire will ask about your sleep in hospital and at home, enabling us to compare your sleep during your hospital stay with your usual sleep patterns. The other questionnaire will ask for your feedback on this information letter. Additionally, you will be asked for some personal details (e.g., age and gender).

Answering the questions will take around 15 to 20 minutes in total. Please place the completed questionnaires in the enclosed envelope and seal it. The sealed envelopes will be collected by staff at your ward the day after they have been distributed and then handed over to the researchers.

Additionally, information about your hospital stay (such as admission diagnosis, medical department, surgeries, admission date and type, number of roommates, and use of sleep medications) will be required. To minimise the time needed for your participation, these details will be gathered by staff from your ward.

Benefits and Risks

Participation in the study does not offer any personal benefits for you. However, it can contribute to a better understanding of sleep during hospital stays and the use of informational materials in studies. The insights gained will help identify needs and areas for improvement. You will not receive individual feedback about the scientific results of the study.

Participation in the study is usually not associated with any significant risks, but a minimal burden cannot be completely ruled out. However, this risk is considered to be very low.

II Data Protection

Study-Specific Information

(1) Data Processing and Usage

In this study, personal data (i.e., personal and health-related information) as defined by Art. 4 para. 1 and Art. 9 of the EU General Data Protection Regulation (DS-GVO) will be collected, processed, and used in paper form. The use of your data is based on legal provisions (pursuant to Art. 6 para. 1(a), Art. 7, and Art. 9 para. 2(a) DS-GVO) and requires your consent before participating in the study.

All information collected about you during the study will initially be pseudonymised (i.e., without mentioning names or identifiable data, using an assigned code of numbers and letters). A coding list, which links full names with pseudonyms, allows the questionnaires to be matched to individuals. However, the use of sealed envelopes ensures that identifying data and questionnaires are always stored separately. Only

one staff member from your ward will have access to the coding list. They will be instructed to permanently destroy the coding list immediately after data collection is complete. From that point onward, the data will be anonymised (i.e., the data can no longer be linked to any specific person).

The sealed envelopes you provide will only be opened by the researchers once the study materials arrive at the study center. All information collected about you during the study will be securely stored and protected from unauthorised access. The anonymised data will be stored and analysed at the Institute of Nursing Science, University of Cologne.

All data will be used solely for scientific research purposes and in accordance with your informed consent. The collected data is intended for use in publications, but you will not be identifiable as a participant. Additionally, the data collected about you in this study may be reused in future research projects (known as "secondary data analyses") and shared in anonymised form with national and international collaborators, both within and outside the University of Cologne.

(2) Duration of Data Storage

The collected data will be stored by the University of Cologne for a period of ten years and will then be deleted.

(3) Right to Withdraw and Data Removal

Participation in the study is voluntary. You can withdraw from the study at any time without providing a reason. This will not result in any disadvantages for you. Upon request, your data will be corrected, anonymised, or deleted. You also have the right to object to further processing of your data or to demand limitations on its use. You can address your objection to the ward supervisor and/or the researchers, either verbally or in writing.

If the data has already been anonymised, it can no longer be linked to you. Therefore, please note that anonymised data and data used in scientific analyses cannot be deleted upon request.

General Information

(1) The responsible parties according to Art. 4 para. 7 DS-GVO are:

University of Cologne
Gesa Diekmann and Alexander May
Albertus-Magnus-Platz
50923 Cologne
Phone: 0221 470 3872
E-mail: dsb@verw.uni-koeln.de
Website: https://verwaltung.uni-koeln.de/stabsstelle02.3/

You can contact our Data Protection Officer using the contact details provided above or our postal address (see above) with the addition "Data Protection Officer."

(2) You have the right to lodge a complaint with a data protection supervisory authority regarding the processing of your personal data within our organisation.

The State Commissioner for Data Protection and Freedom of Information North Rhine-Westphalia:

Helga Block
Kavalleriestraße 2-4
40213 Düsseldorf
Phone: 0211 384240
E-mail: poststelle@ldi.nrw.de
Website: https://www.ldi.nrw.de

(3) If you have any concerns, questions, or complaints regarding data processing and the compliance with data protection regulations, you may of course contact the study director at any time. The contact details can be found at the end of this letter.

(4) The study management will take all reasonable steps to ensure the protection of your data in accordance with the General Data Protection Regulation and other laws. The data will be protected from unauthorised access. Personal data will be anonymised as soon as possible according to the research purpose, unless legitimate interests of the data subject prevent this. Until then, the data will be stored separately with the identifiers that allow personal or material details to be attributed to a specific or identifiable person. These identifiers will only be merged with the individual data when necessary for the research purpose.

(5) The responsible party will only publish personal data if you have expressly consented to such publication.

(6) You have the right to withdraw your data protection consent at any time. The withdrawal of consent will not affect the legality of the processing carried out based on the consent until the withdrawal.

III Contact Study Team

Institute of Nursing Science, Faculty of Medicine, University of Cologne
Prof. Dr phil. Sascha Köpke
Gleueler Straße 176-178
50935 Cologne
Phone: 0221 478 51658
E-mail: sascha.koepke@uk-koeln.de

Sleep Acute

Scientific study to analyse patients' sleep during inpatient hospital care

Responsible according to §4 Abs. 7 DS-GVO:

Institute for Nursing Science, Faculty of Medicine, University of Cologne
Prof. Dr phil. Sascha Köpke
Gleueler Straße 176-178
50935 Cologne
Phone: 0221 478 51658
E-mail: sascha.koepke@uk-koeln.de

Informed Consent for Patients

____ (Name of the person providing information) provided me ____ (Name of the participating person) with information about the nature, scope and significance of the above-mentioned study on _________________ (Date). All my questions were answered satisfactorily.

The study "Sleep Acute" aims to learn more about sleep during inpatient hospital care. Based on this, the study intends to identify needs and derive approaches to improve the current care situation. Additionally, the acceptability and effectiveness of the study's informational materials will be examined. This will help to develop recommendations for recruiting potential study participants in future research projects.

I have been informed that personal data will be processed as part of this study. I am specifically aware of the purpose, scope, legal basis, and duration of data storage. In addition, I am aware of my rights regarding my personal data with respect to the responsible party.
I have received, read, and understood the corresponding written study information.

I have received, read and understood the corresponding written study information.

I am aware that participation is voluntary and that I can revoke it at any time, without providing a reason and without any personal disadvantage, either in writing or verbally. My data will then be completely deleted, and I will receive a notification about this.

I had enough time to think about participating in the study and make a decision.

I agree to participate in the above-mentioned study and consent to the processing of my personal data, as explained to me. Insofar as special personal data, as defined in Art. 9 DS-GVO (such as health data), is collected, my consent also extends to this information.

I have received a copy of this consent declaration.

Place and date	Surname and first name (in block letters) of the participating person	Signature of the participating person

I have informed the person participating in the study both orally and in writing about the objectives, duration, procedure, benefits and all risks of the study. All questions that arose were answered by me clearly and in sufficient detail. The participating person has given their consent voluntarily. I have provided the participating person with the written study information and a copy of this declaration of consent.

Place and date	Surname and first name (in block letters) of the person providing information	Signature of the person providing information
