# Supplementary material for: Effects of a non-standard information leaflet on patient recruitment in acute care: Embedded cluster-randomised controlled trial
Source: PLoS One. 2025 Aug 1;20(8):e0327634. doi: 10.1371/journal.pone.0327634 (PMC12316219; doi:10.1371/journal.pone.0327634)
Supplement: S3 File — (PDF) [file pone.0327634.s003.pdf]

Study Participation

Who can participate in the Study?

You can participate if you are an adult and have spent at least two nights in a regular hospital ward (not a monitoring or intensive care unit) at the time of data collection

What does participation involve?

- If you participate in the study, you will complete a questionnaire about your sleep before and during your hospital stay. You will also evaluate the written study materials and answer questions about yourself.
- The questionnaire takes about 15–20 minutes to complete.
- Once completed, place the questionnaire in the provided envelope. It will be collected one day after distribution.
- Information about your hospital stay will be recorded by a staff member from your ward.

What are the benefits and risks of the Study?

There is no direct benefit to you from participating. However, your contribution is valuable for achieving the study's research goals.

Participation involves minimal burden, but risks cannot be entirely ruled out. These risks are considered to be very low.

Data Protection

- The Data Protection Officers of the University of Cologne[1] are responsible in accordance with Art. 4 Para. 7 DS-GVO.
- If required, if needed, you can contact the study leader[2].
- You also have the right to file a complaint with a data protection authority[3].

Data processing, Usage and storage

- Personal data (including health-related data) will be collected, processed, and used on paper during the study.
- The use of your data requires your written consent.
- Data will initially be pseudonymised (i.e., no identifying information, only a code with letters and numbers). A list linking names to pseudonyms will be maintained and destroyed permanently after data collection. After this, the data will be anonymised (i.e., not traceable to any person).
- Anonymised data will be securely stored at the Institute of Nursing Science at the University of Cologne and protected against unauthorised access. The data will be deleted after ten years.
- During this time, the data will only be used for the purposes of scientific research based on your informed consent. It may be used for publications, future studies, or shared with research partners if necessary.

Right to withdraw and request removal

- Participation in the study is voluntary and can be ended at any time without giving a reason and without any disadvantage to you. You can notify your ward manager or the researchers verbally or in writing to withdraw.
- Fully anonymised data cannot be linked to any person and, therefore, cannot be deleted upon request.

Information about a Research Study by the Institute of Nursing Science at the University of Cologne

Sleep Acute

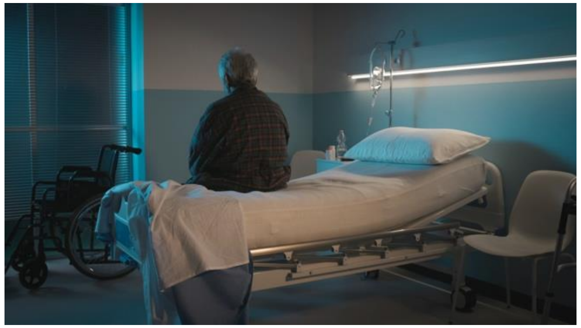

Survey of patients sleep during hospital inpatient care

Dear Patient,

The **Sleep Acute** study aims to learn more about sleep during a hospital stay. You are currently a patient and can support our research by participating **voluntarily**.

Information video

Scan the QR code or use the link to watch a video. The video explains the study's goals and procedures.

https://www.youtube.com/watch?v=TyRctoDIQ_o

In addition to the video, you can find all necessary information for study participation in this flyer.

If you have any unanswered questions, feel free to speak with your ward manager or contact us directly. Our contact details are at the end of the flyer.

Your Study Team

Background and Study Objectives

Sleep is a dynamic state that is vital for health, well-being, and daily functioning. Although illness often increases the need for rest and sleep, previous research show that sleep problems are common during hospital stays.

Our goal is to learn more about sleep and sleep problems in hospitalised patients. This will help identify needs and develop ways to improve care.

We are also analysing the acceptability and effectiveness of our written information materials. This will provide insights for designing materials for future studies.

Contact

[2]Study management

Prof. Dr phil. Sascha Köpke
Institute of Nursing Science, University of Cologne
Gleueler Straße 176-178
50935 Cologne
0221 478 51658
sascha.koepke@uk-koeln.de
https://pflegewissenschaft.uni-koeln.de

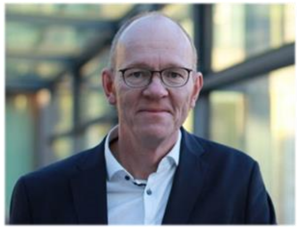

[1]Data Protection Officer, University of Cologne

Gesa Diekmann and Alexander May
Albertus-Magnus-Platz
50923 Cologne
0221 470 3872
dsb@verw.uni-koeln.de
https://verwaltung.uni-koeln.de/stabsstelle02.3/

[3]State Commissioner for Data Protection and Freedom of Information NRW

Helga Block
Kavalleriestraße 2-4
40213 Düsseldorf
0211 384240
poststelle@ldi.nrw.de
https://www.ldi.nrw.de

Consent for a research study by the Institute of Nursing Science at the University of Cologne

Sleep Acute

Survey of patients sleep during hospital inpatient care

Name of the participant:

____

Name of the person providing information:

____

I have received information about the nature, scope, and significance of the study. All my questions were answered satisfactorily.

The **Sleep Acute** study aims to learn more about sleep during inpatient care in hospitals. Based on this, needs are identified and approaches to improving the care situation are derived.

In addition, the acceptability and effectiveness of the study information materials used will be analysed. This serves to develop recommendations for recruiting potential study participants in future research projects.

I have been informed that personal data will be processed in the study. I understand the purpose, scope, legal basis, and duration of data storage. Furthermore, I am aware of my rights regarding the responsible entity.

I have received, read, and understood the corresponding written study information.

I am aware that participation in the study is voluntary and that I can withdraw at any time without providing reasons and without any personal disadvantage.

I agree to participate in the study and consent to the associated processing of my personal data. My consent also extends to special categories of personal data as defined in Art. 9 DS-GVO.

____

Place and date, signature of the participant
