# Supplementary material for: Effects of a non-standard information leaflet on patient recruitment in acute care: Embedded cluster-randomised controlled trial
Source: PLoS One. 2025 Aug 1;20(8):e0327634. doi: 10.1371/journal.pone.0327634 (PMC12316219; doi:10.1371/journal.pone.0327634)
Supplement: S4 File — (PDF) [file pone.0327634.s004.pdf]

Study ID*: _____ _____ - _____ _____ - _____ _____ **Date**: _____._____.__________

*The study ID will be added by members of the study team after the questionnaire has been completed

Questionnaire for Evaluating the Written Study Information

Dear patient,

Thank you for participating in this survey. In this questionnaire, we kindly ask you to evaluate the information letter you received about the study. Providing as complete answers as possible will help us better understand your impressions.

Please answer the following questions by selecting the value on the response scales that applies to you.

1. How do you evaluate the fact that you were provided with written study information?

0 1 2 3 4 5

Very negative — Very positive

2. Did the written study information encourage you to participate in the study?

0 1 2 3 4 5

Not at all — Very much

3. How do you rate the written study information overall?

0 1 2 3 4 5

Very negative — Very positive

4. How do you evaluate the format and design of the written study information?

0 1 2 3 4 5

Very negative — Very positive

5. How do you rate the written study information in terms of its clarity and understandability?

0	1	2	3	4	5
Not understandable at all					Very understandable

6. How do you evaluate the written study information in terms of its completeness?

0	1	2	3	4	5
Not complete					Complete

7. How do you evaluate the written study information in terms of its scope?

0	1	2	3	4	5
Not appropriate at all					Completely appropriate

8. How do you evaluate the content of the written study information in terms of its relevance?

0	1	2	3	4	5
Not relevant at all					Very relevant

Please write you're answers below each question.

9. What do you particularly like about the written study information?

10. What do you like less about the written study information?

11. What should be changed in the written study information?

At the end of the questionnaire, we kindly ask you to provide the following information about yourself:

12. Please indicate your highest level of education.

☐ No school leaving certificate
☐ Lower secondary school certificate
☐ Intermediate school certificate
☐ University entrance qualification
☐ Completed vocational training
☐ (Specialised) university degree
☐ Doctorate

13. Please indicate your employment status.

☐ School student
☐ University student
☐ Apprentice
☐ Employed
☐ Self-employed
☐ Job-seeking/ unemployed
☐ Housewife or househusband
☐ Retired
☐ Other: ____

14. Please specify your nationality.

____

15. Please specify your country of birth.

____

16. Please specify your native language.

____

Thank you for participating in the survey!
