# Supplementary material for: Effects of a non-standard information leaflet on patient recruitment in acute care: Embedded cluster-randomised controlled trial
Source: PLoS One. 2025 Aug 1;20(8):e0327634. doi: 10.1371/journal.pone.0327634 (PMC12316219; doi:10.1371/journal.pone.0327634)
Supplement: S5 Table — (DOCX) [file pone.0327634.s005.docx]

|  | Small hospitals  (<250 beds) | Medium hospitals  (250-399 beds) | Large hospitals  (400-599 beds) | Very large hospitals    (≥600 beds) |
| --- | --- | --- | --- | --- |
| Number of hospitals | 14 | 9 | 5 | 3 |
| Number of wards per hospital | 2 | 3 | 5 | 8 |

**Planned sample size (stratification by hospital size)**

S5a Table. Planned sample size (stratification by hospital size)

|  | Small hospitals  (<250 beds) | Medium hospitals  (205-399 beds) | Large hospitals  (400-599 beds) | Very large hospitals    (≥600 beds) |
| --- | --- | --- | --- | --- |
| Number of hospitals | 7 | 4-5 | 2-3 | 1-2 |
| Number of wards per hospital | 2 | 3 | 5 | 8 |

**Planned sample size after adjustment (stratification by hospital size)**

S5b Table. Planned sample size after adjustment (stratification by hospital size)
