# Supplementary material for: Effects of a non-standard information leaflet on patient recruitment in acute care: Embedded cluster-randomised controlled trial
Source: PLoS One. 2025 Aug 1;20(8):e0327634. doi: 10.1371/journal.pone.0327634 (PMC12316219; doi:10.1371/journal.pone.0327634)
Supplement: S7 File — (PDF) [file pone.0327634.s007.pdf]

Studienprotokoll

Sleep Acute

Teil I: Schlaf von Patient*innen während der stationären Versorgung im Krankenhaus

Teil II: Akzeptanz und Wirksamkeit verschiedener Informationsschreiben zur Rekrutierung von Patient*innen im Krankenhaus

MaSoLa – MagenSondenLage

Teil III: Entscheidungsfindung von Pflegefachpersonen zur Verifikation der korrekten Lage blind applizierter Magensonden im Krankenhaus

Marcelina Roos, B.Sc.

Wissenschaftliche Mitarbeiterin

Markus Grebe, M.Sc., M.Sc. PH

Wissenschaftlicher Mitarbeiter

Martin N. Dichter, Ph.D.

PostDoc, Wissenschaftlicher Mitarbeiter

Prof. Dr. phil. Sascha Köpke

Institutsleitung

Institut für Pflegewissenschaft

Medizinische Fakultät der Universität zu Köln

Gleueler Straße 176-178

D-50935 Köln

Abstract – Teil I

Hintergrund

Schlaf ist ein komplexer und dynamischer Zustand, der für die Gesundheit, das körperliche und geistige Wohlbefinden sowie das tägliche Funktionieren von entscheidender Bedeutung ist. Obgleich Krankheit und Verletzungen mit einem gesteigerten Bedürfnis nach Ruhe und Schlaf einhergehen, lassen bisherige wissenschaftliche Untersuchungen vermuten, dass Schlafprobleme während eines Krankenhausaufenthaltes regelhaft auftreten und von Patient*innen als Stressor erlebt werden. Die Gründe hierfür sind vielfältig. Zeitgleich fehlen derzeit groß angelegte, multizentrische Studien und damit aussagekräftige Forschungsergebnisse zur Abbildung und Bewertung der derzeitigen Schlafsituation von Krankenhauspatient*innen.

Ziele

Im Rahmen der Studie „Sleep Acute“ werden die nachfolgenden Forschungsziele verfolgt:

1. Beschreibung der Prävalenz von Schlafproblemen bei Krankenhauspatient*innen
2. Beschreibung der subjektiven Schlafqualität von Krankenhauspatient*innen
3. Identifikation von krankenhaus- und personenbezogenen Faktoren, die mit Schlafproblemen bei Krankenhauspatient*innen in Beziehung stehen
4. Identifikation von aktuell angewendeten Strategien und Maßnahmen zur Schlafförderung bei Krankenhauspatient*innen

Methodik

Die Verfolgung der Forschungsziele erfolgt anhand einer multizentrischen Beobachtungsstudie. Zwischen November und Dezember 2020 wird in zufällig ausgewählten Krankenhäusern im Umkreis von 50 km um das Studienzentrum, eine querschnittliche Befragung von rund 1.000 Patient*innen und rund 100 Pflegefachpersonen durchgeführt. Ausgehend von Erkenntnisinteressen und Vorwissen, erfolgen Datenerhebung und -auswertung mittels qualitativer (Forschungsziel 4) und quantitativer (Forschungsziele 1 bis 4) Verfahren.

Erwartete Ergebnisse

Es wird erwartet, dass die Durchführung der Studie ermöglicht, die derzeitige Situation zum Thema Schlaf im Krankenhaus valide abzubilden. Die generierten Erkenntnisse dienen zum einen der Prüfung bisheriger Annahmen und zum anderen dem Erhalt neuer Einblicke in die aktuelle Versorgungspraxis. Beides bietet wiederum die Möglichkeit, Bedarfe aufzudecken und zukünftige Forschungsfragen abzuleiten.

Abstract – Teil II

Hintergrund

Eine wesentliche Komponente im empirischen Forschungsprozess ist die Rekrutierung von Teilnehmenden. Forschenden stehen hierfür eine Vielzahl unterschiedlicher Rekrutierungsstrategien, -techniken und -verfahren zur Verfügung. Während Studien erste Hinweise dafür liefern, dass der Einsatz gebräuchlicher Studieninformationsschreiben eine potentielle Barriere darstellt, um Studienteilnehmende zu gewinnen, fehlen zum aktuellen Zeitpunkt aussagekräftige Forschungserkenntnisse zur Akzeptanz und Wirksamkeit klassisch sowie alternativ gestalteter schriftlicher Studieninformationsmaterialien.

Ziel

Forschungsziel ist die Untersuchung der Akzeptanz und Wirksamkeit eines ansprechend gestalteten Studieninformationsflyers im Vergleich zu einem formalen Studieninformationsschreiben zur Rekrutierung von Teilnehmenden in einer Fragebogenstudie zum Schlaf im Krankenhaus.

Methodik

Als Bestandteil der in Teil I des vorliegenden Studienprotokolls abgebildeten querschnittlichen Beobachtungsstudie zum Schlaf hospitalisierter Patient*innen wird eine eingebettete Cluster-randomisierte Studie (SWAT) zum Vergleich zweier schriftlicher Studieninformationen durchgeführt. Hierfür werden die rund 1.000 Krankenhauspatient*innen Cluster-randomisiert zwei Studiengruppen zugeteilt, die sich hinsichtlich des verwendeten schriftlichen Studieninformationsmaterials unterscheiden. Zur Wirksamkeitsprüfung beider Ansätze werden der Rücklauf sowie die soziodemographische Vielfalt der Stichprobe anhand quantitativer Verfahren analysiert. Eine Bewertung der Akzeptanz erfolgt mittels Fragebogen. Quantitative Fragebogenanteile werden deskriptiv, qualitative Anteile inhaltsanalytisch ausgewertet.

Erwartete Ergebnisse

Es wird erwartet, dass die Durchführung der Studie die Ermittlung der Akzeptanz und Wirksamkeit zweier schriftlicher Studieninformationen ermöglicht. Die Erkenntnisse dienen der Ableitung konkreter Empfehlungen für die Gestaltung einer Komponente des empirischen Forschungsprozesses und damit der erfolgreichen Generierung sowie Verbreitung wissenschaftlicher Forschungsergebnisse.

Abstract – Teil III

Hintergrund

Die Anlage von nasogastralen Sonden erfolgt in der klinischen Praxis oftmals ärztlich delegiert durch Pflegefachpersonen. Zu den in der Literatur dokumentierten Komplikationen zählen neben anderen die fehlerhafte tracheale Platzierung, Blutungen durch traumatisierte Mucosa, die Perforation des Ösophagus sowie die Entstehung von Pneumothoraces. Die meisten US-amerikanischen Leitlinien empfehlen die radiologische Überprüfung der Magensondenlage als „first-line“- Methode. Wiederum verweisen Empfehlungen aus Europa und Australien auf die Durchführung einer Röntgenkontrolle, wenn die Methode mittels Messung des pH-Werts versagt oder gewisse Risiken bestehen. Von den nichtradiologischen Methoden wird die pH-Wert-Messung am meisten, die auskultatorische Methode hingegen am wenigsten empfohlen. Für Deutschland liegt derzeit keine evidenzbasierte Empfehlung vor. Betrachtet man das beschriebene Vorgehen zur Verifikation der Lage nasogastraler Sonden in einem gebräuchlichen Pflegelehrbuch, so werden die Aspiration von Magensekret inkl. der Messung des pH-Werts sowie die Methode der Auskultation empfohlen. Zu einer Röntgenkontrolle wird erst bei Zweifelsfällen geraten. Internationale nicht repräsentative Befragungsergebnisse hinsichtlich der Methoden zur Verifikation der korrekten Lage von Magensonden durch Pflegefachpersonen lassen eine Diskrepanz zwischen Empfehlungen von Fachgesellschaften und der Handhabung in der Praxis erahnen. Bisher liegen für Deutschland keine Studien vor, welche das Vorgehen hinsichtlich der Verifikation der Lagekontrolle systematisch untersuchen. Auch gibt es diesbezüglich derzeit keine evidenzbasierten Empfehlungen deutscher Fachgesellschaften.

Ziele

Mit den folgenden Forschungsfragen soll im Rahmen des Projektes „MaSoLa - MagenSondenLage“ die Entscheidungsfindung hinsichtlich der Bestimmung der korrekten Lage blind applizierter Magensonden auf Intensivstationen in deutschen Krankenhäusern exploriert werden:

1. Welche diagnostischen Methoden setzen die Pflegefachpersonen zur Verifikation der korrekten Lage einer Magensonde ein?
2. Entspricht das Vorgehen zur Verifikation den verfügbaren best-practice bzw. evidenzbasierten Kriterien?
3. Wie schätzen die Pflegefachpersonen die Eignung bekannter Methoden zur Verifikation ein?

Methodik

Zur Beantwortung der Forschungsfragen ist eine multizentrische Beobachtungsstudie in einer repräsentativen Auswahl von Krankenhäusern im Umkreis von 50 km um das Studienzentrum geplant.

Der Feldzugang erfolgt über das in Teil I des vorliegenden Studienprotokolls beschriebene Forschungsvorhaben. Befragt werden Stationsleitungen und Praxisanleiter*innen aller Intensivstationen der rund 30 eingeschlossenen Einrichtungen. Datenerhebung und -auswertung folgen qualitativen und quantitativen Methoden.

Erwartete Ergebnisse

Die zu erwartenden Ergebnisse sollen einen ersten Hinweis dazu liefern, wie klinisch tätige Intensivpflegefachpersonen in Deutschland den Einsatz und die Eignung unterschiedlicher Methoden zur Überprüfung der korrekten Magensondenlage einschätzen. Außerdem ermöglichen die Ergebnisse eine Kontrastierung mit den Empfehlungen internationaler Fachgesellschaften sowie den in Deutschland im Rahmen der Berufsausbildung vermittelten Methoden.

INHALTSVERZEICHNIS

TEIL I: SCHLAF VON PATIENT*INNEN WÄHREND DER STATIONÄREN VERSORGUNG IM AKUTKRANKENHAUS ... 1

Hintergrund ... 1

Ziele ... 2

Methodik ... 2

Design ... 2

Stichprobe und Rekrutierung ... 3

Datenerhebung ... 5

Datenmanagement ... 8

Datenanalyse ... 9

TEIL II: AKZEPTANZ UND WIRKSAMKEIT VERSCHIEDENER INFORMATIONSSCHREIBEN ZUR REKRUTIERUNG VON PATIENT*INNEN IM KRANKENHAUS ... 10

Hintergrund ... 10

Ziele ... 11

Methodik ... 11

Design ... 11

Stichprobe ... 12

Rekrutierung ... 12

Datenerhebung ... 13

Datenanalyse ... 13

TEIL III: ENTSCHEIDUNGSFINDUNG VON PFLEGEFACHPERSONEN ZUR VERIFIKATION DER KORREKTEN LAGE BLIND APPLIZIERTER MAGENSONDEN IM KRANKENHAUS ... 15

Hintergrund ... 15

Ziele ... 16

Methodik ... 16

Design ... 16

Stichprobe und Rekrutierung ... 17

Datenerhebung ... 18

Datenmanagement ... 19

Datenanalyse ... 19

ETHISCHE ÜBERLEGUNGEN ... 20

Aufklärung und Einwilligung ... 20

Mögliche Risiken ... 20

Datenschutz ... 23

LITERATURVERZEICHNIS ... 25

ANHANG ... X

Anhang 1: Consensus Sleep Diary (CSD) – Kernversion X

Anhang 2: Consensus Sleep Diary (CSD) – Zusätzliche Items X

Anhang 3: Sleep Disturbance Item Bank – Short Form 8a X

Anhang 4: Richards-Campbell Sleep Questionnaire (RCSQ) X

Anhang 5: Fragen zu krankenhausspezifischen Faktoren X

Anhang 6: Fragebogen zur Identifikation derzeit angewendeter Strategien und Maßnahmen zur Schlafförderung X

Anhang 7: Informations- und Einwilligungsschreiben für Patient*innen (Teil I und II) XI

Anhang 8a: Informations- und Einwilligungsflyer für Patient*innen (Teil I und II) XXIX

Anhang 8b: Skript für das Informationsvideo für Patient*innen (Teil I und II) XXXII

Anhang 9: Fragebogen zur Bewertung der schriftlichen Studieninformation XXXIV

Anhang 10: Informations- und Einwilligungsschreiben für Pflegefachpersonen (Teil I) XXXVII

Anhang 11: Informationsschreiben (Teil III) und Fragebogen zur Entscheidungsfindung hinsichtlich der Verifikation der korrekten Magensondenlage XLIII

TEIL I: SCHLAF VON PATIENT*INNEN WÄHREND DER STATIONÄREN VERSORGUNG IM AKUTKRANKENHAUS

Hintergrund

Schlaf ist ein komplexer und dynamischer Zustand, der für die Gesundheit, das körperliche und geistige Wohlbefinden sowie das tägliche Funktionieren von entscheidender Bedeutung ist (Redeker, Hedges & Booker, 2011). Obgleich Krankheit und Verletzungen mit einem gesteigerten Bedürfnis nach Ruhe und Schlaf einhergehen (Friese, 2008), lassen bisherige wissenschaftliche Untersuchungen vermuten, dass Schlaf während eines Krankenhausaufenthaltes ein ebenso komplexes Problem(-feld) darstellt.

Bisher vorliegende Studien in ausgewählten Patient*innenpopulationen liefern erste Anhaltspunkt dafür, dass Schlafprobleme im Krankenhaus regelhaft auftreten und von Patient*innen als Stressor erlebt werden (Gellerstedt, Medin & Rydell Karlsson, 2014; Lane & East, 2008; Wesselius et al., 2018). Beeinträchtigungen des Schlafes gehen dabei mit verschiedenen Risiken einher. Beispiele hierfür sind zum einen negative Auswirkungen auf Immun- und Stoffwechselprozesse (Ganz, 2012; Hoevenaar-Blom, Spijkerman, Kromhout & Verschuren, 2014; Lange, Dimitrov & Born, 2010), ein verstärktes Schmerzempfinden (Raymond, Nielsen, Lavigne, Manzini & Choiniere, 2001) sowie ein vermehrtes Auftreten von Stürzen (Stone, Ensrud & Ancoli-Israel, 2008) und Deliren (Weinhouse, Schwab, Watson, Patil, Vaccaro, Pandharipande & Ely, 2009). Zum anderen können Schlafprobleme kognitive und emotionale Funktionen, wie etwa die Verarbeitung neuer Informationen, die Entscheidungsfindung sowie die Bewältigung herausfordernder Situationen, beeinträchtigen (John, Edet, Mgbekem, Robinson-Bassey, Duke, Esienumoh & Ndebbio, 2007; Pilkington, 2013; Rasch & Born, 2013). Folglich bergen Schlafprobleme in vielerlei Hinsicht die Gefahr, nachteilig Einfluss auf die Gesundheit, die Genesung von Krankheit und das (Wohl-)Befinden von Patient*innen während eines Krankenhausaufenthaltes zu nehmen (Raymond et al., 2001).

Angesichts der gravierenden Risiken von Schlafproblemen, stellt sich die Frage nach Maßnahmen und Strategien zur Schlafförderung im Krankenhaus. Grundlegend hierbei ist die Identifizierung relevanter und vor allem potentiell modifizierbarer Faktoren, die mit Schlafproblemen verbunden sind (Wesselius et al., 2018). Mittels qualitativer Forschungsansätze wurde bereits eine Vielzahl dieser Faktoren aufgedeckt (Pilkington et al., 2013). Bisher fehlen jedoch groß angelegte, multizentrische Studien, welche, auf den bisherigen Erkenntnissen aufbauend, die Zusammenhänge zu Schlafproblemen untersuchen und gezielt Ansätze zur Schlafförderung ableiten. Wie aktuell im Versorgungsalltag in Krankenhäusern mit Schlafproblemen von Patient*innen umgegangen wird und welche Maßnahmen und Strategien zur Schlafförderung dabei Verwendung finden, ist zum jetzigen Zeitpunkt weitestgehend unbekannt. In der wissenschaftlichen Literatur findet sich jedoch ein Konsens darüber,

dass Pflegefachpersonen durch ihre Nähe zu Patient*innen für die Initiierung und Durchführung von Maßnahmen und Strategien zur Schlafförderung in einer einzigartigen Position sind (Pellatt, 2007; Radtke, Obermann & Teymer, 2014, Redeker et al., 2011).

Ziele

Schlaf im Krankenhaus stellt ein bedeutsames, bisher jedoch nicht umfassend untersuchtes Forschungsfeld dar. In Anbetracht dessen, ist die Durchführung einer multizentrischen Studie in einer repräsentativen Auswahl von Krankenhäusern im Umkreis von 50 km um das Studienzentrum geplant. Hierbei werden die nachfolgenden Forschungsziele verfolgt:

1. Beschreibung der Prävalenz von Schlafproblemen bei Krankenhauspatient*innen
2. Beschreibung der subjektiven Schlafqualität von Krankenhauspatient*innen
3. Identifikation von krankenhausbezogenen Faktoren, die mit Schlafproblemen bei Krankenhauspatient*innen in Beziehung stehen
4. Identifikation von aktuell angewendeten Strategien und Maßnahmen zur Schlafförderung bei Krankenhauspatient*innen

Die Verfolgung der Forschungsziele soll ermöglichen, die derzeitige Schlafsituation in deutschen Krankenhäusern valide abzubilden. Die generierten wissenschaftlichen Erkenntnisse dienen zum einen der Prüfung bisheriger Annahmen und zum anderen der Exploration der aktuellen Versorgungspraxis. Beides bietet wiederum die Möglichkeit, Bedarfe aufzudecken und zukünftige Forschungsfragen abzuleiten. Das Vorgehen zielt langfristig darauf, eine Verbesserung der Versorgungspraxis zu bewirken.

Methodik

Design

Die Verfolgung der Forschungsziele erfolgt anhand einer multizentrischen Beobachtungsstudie, deren methodisches Vorgehen an ein in den Niederlanden durchgeführtes Forschungsvorhaben (Wesselius et al., 2018) angelehnt ist. Zwischen November und Dezember 2020 wird in zufällig ausgewählten Krankenhäusern rund um das Studienzentrum, eine querschnittliche Befragung von Patient*innen und Pflegefachpersonen durchgeführt. Ausgehend von Erkenntnisinteressen und Vorwissen, erfolgen Datenerhebung und -auswertung mittels qualitativer (Forschungsziel 4) und quantitativer (Forschungsziele 1 bis 4) Verfahren.

Stichprobe und Rekrutierung

Da die Studie explorativ angelegt ist, gibt es derzeit weder valide Anhaltspunkte für eine Stichprobenkalkulation noch für eine Werteverteilung in der Zielpopulation. Gebildet wird eine Stichprobe, die trotz dessen groß genug erscheint, um hinreichend aussagekräftige Ergebnisse zu erzielen. Angestrebt wird eine Teilnahme von mindestens 1.000 Patient*innen aus rund 30 Krankenhäusern. Hierfür erfolgt ein Einschluss von ca. 100 Stationen. Neben den Patient*innen soll von jeder teilnehmenden Station eine Pflegefachperson für eine Studienteilnahme gewonnen werden.

Die jeweiligen Ein- und Ausschlusskriterien für Krankenhäuser, Stationen, Patient*innen sowie Pflegefachpersonen sind Tabelle 1 zu entnehmen.

Tabelle 1: Ein- und Ausschlusskriterien

Ebene	Einschlusskriterien	Ausschlusskriterien
**Krankenhäuser**	▪ Allgemeine Krankenhäuser (vollstationäre Fachabteilungen)<br>▪ Krankenhäuser, die mindestens eine Grundversorgung anbieten (Innere Medizin und Chirurgie)<br>▪ Krankenhäuser im Umkreis von 50 km um das Studienzentrum	▪ Sonstige Krankenhäuser (ausschließlich psychiatrische oder neurologische Betten oder ausschließlich teilstationäre Patient*innenversorgung)<br>▪ Bundeswehrkrankenhäuser<br>▪ Belegkrankenhäuser<br>▪ Vorsorge- und Rehabilitationseinrichtungen
**Stationen**	▪ Bettenführende Stationen<br>▪ Normalstationen	▪ Palliativstationen<br>▪ Psychiatrische Stationen<br>▪ Pädiatrische Stationen<br>▪ Notaufnahmen<br>▪ Funktionsbereiche<br>▪ Überwachungs- und Intensivstationen
**Patient*innen**	▪ Alter ≥ 18 Jahre<br>▪ Einwilligungsfähigkeit<br>▪ Aufenthalt von ≥ zwei Nächten auf einer Normalstation im Krankenhaus zum Zeitpunkt der Datenerhebung<br>▪ Vorliegen der Informierten Einwilligung für eine Studienteilnahme	▪ Kognitive und/oder körperliche Beeinträchtigungen, die das Ausfüllen des Fragebogens nicht zulassen (klinische Entscheidung der Stationsleitung)<br>▪ Unzureichende Kenntnisse der deutschen Sprache, die das Ausfüllen des Fragebogens nicht zulassen (klinische Entscheidung der Stationsleitung)
**Pflegefachpersonen**	▪ Stationsleitung oder stellvertretende Stationsleitung einer der teilnehmenden Stationen	

	▪ Stellenumfang von ≥ 50 Prozent der regelmäßigen wöchentlichen Arbeitszeit<br>▪ Vorliegen der Informierten Einwilligung für eine Studienteilnahme	

Auf Basis vorliegender Klinikverzeichnisse wurden im Juli 2020 94 Krankenhäuser identifiziert, welche den zuvor aufgeführten Ein- und Ausschlusskriterien entsprechen. Ausgehend von dem Ziel einer möglichst repräsentativen Einrichtungsauswahl, wird hieraus eine stratifizierte Zufallsstichprobe gebildet. Dabei werden vier Strata nach vorher festgelegten Einrichtungsgrößen, gemessen an der Anzahl der Betten, erstellt (kleine Einrichtungen ≙ < 250 Betten; mittelgroße Einrichtungen ≙ 250-399 Betten; große Einrichtungen ≙ 400-599 Betten; sehr große Einrichtungen ≙ > 600 Betten). Die randomisierte Auswahl der Häuser erfolgt mithilfe einer online erhältlichen Software (https://www.random.org/sequences). Davon ausgehend, dass nicht alle Einrichtungen an dem Forschungsvorhaben teilnehmen, werden von vornherein mehr als die geplanten 30 Krankenhäuser bestimmt. Im weiteren Verlauf werden daher Pflegedirektionen von zunächst 60 Einrichtungen angeschrieben und in der Folge telefonisch kontaktiert. Auf Wunsch wird das Forschungsvorhaben zusätzlich in einem persönlichen Gespräch vor Ort präsentiert.

Entsprechend der Einrichtungsgrößen, ist jeweils ein Einschluss von zwei bis acht Stationen vorgesehen (kleine Einrichtungen: n = ca. 2 Stationen; mittelgroße Einrichtungen: n = ca. 3 Stationen; große Einrichtungen: n = ca. 5 Stationen; sehr große Einrichtungen: n = 8 Stationen). Die Auswahl der Stationen erfolgt durch die Pflegedirektionen der teilnehmenden Krankenhäuser. Diese werden zur Vermeidung eines Selektionsbias gebeten, dabei möglichst zufällig vorzugehen.

Auf den teilnehmenden Stationen selbst, werden grundsätzlich alle Patient*innen eingeschlossen, die sich im Datenerhebungszeitraum vor Ort befinden und den zuvor aufgeführten Ein- und Ausschlusskriterien entsprechen. Die Rekrutierung erfolgt durch zuvor festgelegte Kontaktpersonen auf den Stationen (i. d. R. die Stationsleitungen). Diese werden im Vorfeld durch das Forschungsteam über das Forschungsvorhaben informiert. Zusätzlich werden alle auf den teilnehmenden Stationen tätigen Pflegefachpersonen, welche den Ein- und Ausschlusskriterien entsprechen, um eine Studienteilnahme gebeten. Hierfür werden die Kontaktpersonen aufgefordert, bei eigenem Interesse bzw. dem von Kolleg*innen, Kontakt zum Forschungsteam aufzunehmen.

Um sowohl auf Einrichtungs- als auch auf Stationsebene die Bereitschaft zur Studienteilnahme sowie die Rücklaufquote zu erhöhen, werden verschiedene Strategien angewendet (Edwards et al., 2009). Hierzu gehören ein enger persönlicher Kontakt zu den Kontaktpersonen in den teilnehmenden

Krankenhäusern, die Ankündigung einer strukturierten Rückmeldung der Forschungsergebnisse für alle Häuser sowie eine Verlosung von fünf Geldpreisen im Gesamtwert von 1.000 Euro unter den teilnehmenden Stationen, unabhängig von der jeweiligen Rücklaufrate. Zur Auslosung der Geldpreise wird die bereits für die Stichprobenbildung herangezogene Randomisierungssoftware (https://www.random.org/sequences) von dem Forscher*innenteam verwendet.

Datenerhebung

Die Datenerhebung setzt sich aus einer papier-basierten Befragung (Fragebögen) von Patient*innen und Pflegefachpersonen zusammen.

Instrumente

Zur Erfassung von **Schlafproblemen** und **subjektiver Schlafqualität** von Patient*innen werden das „Consensus Sleep Diary“ (CSD), Items aus der „Sleep Disturbance Item Bank“ des Patient Reported Outcomes Measurement Information System (PROMIS) sowie der „Richards-Campbell Sleep Questionnaire“ (RCSQ) verwendet. Entsprechend der Definition von „Schlafqualität“ von Buysse, Reynolds, Monk, Berman und Kupfer (1989), werden hierbei sowohl Informationen zu qualitativen als auch zu quantitativen Aspekten von Schlaf gesammelt. Die drei Instrumente sind in den Anhängen 1 bis 4 abgebildet.

Das „Consensus Sleep Diary“ (CSD) (Carney, Buysse, Ancoli-Israel, Edinger, Krystal, Lichstein & Morin, 2012) ist ein konsentierter, standardisierter Fragebogen in Form eines Schlaftagebuchs. Dieser eignet sich sowohl für den kurz- als auch den langfristigen Gebrauch. Ziel des CSD ist die Erfassung der quantitativen Dimensionen der Schlafqualität. Die Kernversion enthält acht Items. Erfragt werden die Zeitpunkte des Zubettgehens und des Einschlafens, die Dauer bis zum Einschlafen, Häufigkeit, Dauer und letzter Zeitpunkt des Aufwachens, die Aufstehzeit sowie die insgesamt wahrgenommene Schlafqualität. Zusätzlich bietet ein Freitextfeld Platz für Kommentare. Neben der CSD-Kernversion liegen Erweiterungen des Instruments mit zusätzlichen optionalen Items vor. Zwei dieser Items, die sich auf das Abhalten eines Mittagsschlafs und den Gebrauch von Schlafmedikamenten beziehen, werden aufgrund ihrer Relevanz für das Forschungsvorhaben in der Datenerhebung ergänzend verwendet. Das CSD liegt nicht in deutscher Sprache vor. Eine Instrumentenübersetzung, orientiert an Beaton, Bombardier, Guillemin und Ferraz (2000), befindet sich daher in Vorbereitung.

Die „Sleep Disturbance Item Bank“ des Patient Reported Outcomes Measurement Information System (PROMIS) (Buysse et al., 2010) zielt auf die Erfassung qualitativer Aspekte von Schlaf. Die Items werden hierbei in Form von Aussagen präsentiert und anhand einer fünfstufigen Likert-Skala („überhaupt nicht“ bis „sehr“) dahingehend bewertet, wie gut sie der aktuellen Situation entsprechen. Die in der „Sleep Disturbance Item Bank“ enthaltenen Fragen wurden bereits in verschiedenen

Patient*innengruppen angewendet und überprüft. Hierbei zeigten sich ausgezeichnete Messeigenschaften (ebd.). Neben einer 27 Items-langen Originalversion, existieren vier gekürzte Instrumentenversionen. Ausgehend von Aspekten der Praktikabilität sowie der Relevanz der enthaltenen Items für das eigene Forschungsvorhaben, wird die „Sleep Disturbance Item Bank – Short Form 8a“ mit acht Items eingesetzt. Hierin sind die Bereiche Schlafqualität, Schlafprobleme sowie schlafbezogene Erholung enthalten. Die „Sleep Disturbance Item Bank“ wurde bereits vollständig ins Deutsche übersetzt. Die deutschsprachige Version wird derzeit über die Webseite des PROMIS (http://promis-germany.de/instrumente/) erworben.

Bei dem „Richards-Campbell Sleep Questionnaire“ (RCSQ) (Richards, 1987) handelt es sich um einen weit verbreiteten und häufig eingesetzten Fragebogen, um Schlaf und Schlafqualität von kritisch kranken Patient*innen zu erfassen. Anhand von fünf Items werden Informationen zu Schlaftiefe, Einschlafen, Aufwachen, erneutem Einschlafen und Schlafqualität gesammelt. Die Bewertung erfolgt jeweils anhand einer visuellen Analogskala. Der RCSQ liegt bereits als deutschsprachige Version vor. Eine an den Übersetzungsprozess anschließende Überprüfung der Internen Konsistenz zeigte zufriedenstellende Testergebnisse (Krotsetis, Richards, Behncke & Köpke, 2017).

Mit dem Ziel, Schlafprobleme und subjektive Schlafqualität von Patient*innen im Krankenhaus mit dem gewöhnlichen Schlaf in Bezug zu setzen, wird jedes Item der zuvor beschriebenen Instrumente zweimal abgefragt. Hierbei werden jeweils der durchschnittliche Schlaf zu Hause während des Monats vor dem Krankenhausaufenthalt und der Schlaf im Krankenhaus in der Nacht vor der Datenerhebung bewertet. In Anbetracht der Abstraktheit der Frage nach einer durchschnittlichen Einschätzung des Schlafs für den Zeitraum von einem Monat, werden die Teilnehmenden zusätzlich darum gebeten jeweils anzugeben, wie sicher sie sich bei der Beantwortung fühlen. Antwortoptionen befinden sich auf einer vierstufigen Likert-Skala („überhaupt nicht sicher“ bis „sehr sicher“). Diese zusätzlichen Informationen dienen der Einschätzung der Datenqualität.

Die Items zur Erfassung von Schlafproblemen und subjektiver Schlafqualität werden durch drei weitere Fragen zu **krankenhausspezifischen Faktoren**, von denen angenommen wird, im Zusammenhang mit Schlafproblemen zu stehen, ergänzt. Hierbei wird um eine Auswahl der Faktoren gebeten, die mit dem Einschlafen, dem nächtlichen Aufwachen sowie dem finalen Aufwachen während der vorherigen Nacht im Krankenhaus in Verbindung stehen. Mögliche Faktoren werden in Form einer Liste präsentiert. Zudem erlaubt ein zusätzliches Freitextfeld die Ergänzung weiterer relevanter Faktoren. Bei allen drei Fragen ist eine Mehrfachauswahl möglich. Die Items zu den krankenhausspezifischen Faktoren wurden von Wesselius et al. (2018) bereits erfolgreich eingesetzt. Die Originalfragen sind in Anhang 5 abgebildet.

Zur Identifikation derzeit angewendeter **Strategien und Maßnahmen zur Schlafförderung** wird ein Fragebogen (Anhang 6) verwendet, der gleichermaßen quantitative und qualitative Anteile enthält. Der Fragebogen wurde gemeinsam im Forschungsteam, basierend auf dem derzeitigen Stand der Forschung (u. a. Eliassen & Hopstock, 2011; Kauffmann, Heinemann, Himmel, Hußmann, Schlott & Weiß, 2018; Redeker et al., 2011; Salzmann-Erikson, Lagerqvist & Pousette, 2015), entwickelt.

Im ersten Teil des Fragebogens werden Listen mit möglichen Strategien und Maßnahmen zur Schlafförderung präsentiert. Die befragten Personen werden gebeten, hieraus die ihnen bekannten, die aktuell umgesetzten sowie die wünschenswerten Strategien und Maßnahmen auszuwählen. Freitextfelder erlauben die Erweiterung der Listen mit zusätzlichen Antwortoptionen. Im zweiten Teil des Fragebogens werden den befragten Personen offen formulierte Fragen zur derzeitigen Umsetzung von Strategien und Maßnahmen zur Schlafförderung im Versorgungsalltag gestellt. Zuletzt folgt eine Aufzählung mit Aussagen, welche auf den Stellenwert von Schlafförderung und die Rolle der Pflegefachpersonen bei der Initiierung und Durchführung der Strategien und Maßnahmen zielen. Diese werden anhand einer vierstufigen Likert-Skala („stimme überhaupt nicht zu“ bis „stimme voll und ganz zu“) bewertet.

Im Vorfeld der Datenerhebung erfolgt ein kognitives Pretesting des entwickelten Fragebogens. Hierfür werden fünf Pflegefachpersonen, die den festgelegten Ein- und Ausschlusskriterien entsprechen, über das berufliche Netzwerk der Forschenden rekrutiert. Bei Bedarf werden notwendige Anpassungen des Fragebogens abgeleitet.

Neben den bisher aufgeführten Instrumenten werden eine Reihe von Einzelitems in die Datenerhebung integriert, die der **Stichprobenbeschreibung** sowie der späteren Datenanalyse dienen. Von Patient*innen werden soziodemographische (Alter und Geschlecht) sowie klinische Angaben (Fachbereich, Aufnahmediagnose, Operation[en], Aufnahmetag und -art, Anzahl der Mitpatient*innen im Zimmer sowie Schlafmedikamente) erfasst. Von den teilnehmenden Pflegefachpersonen werden Daten zu Alter, Geschlecht, Fachbereich sowie Berufsqualifikation und -erfahrung gesammelt.

Vorgehensweise

In einem ersten Schritt werden die gesamten Studiendokumente von den Forschenden an festgelegte Kontaktpersonen (i. d. R. Stationsleitungen) übergeben. Diese koordinieren die Datenerhebung auf den teilnehmenden Stationen und verantworten das Verteilen sowie das Einsammeln der Studienmaterialien.

Allen Patient*innen, die den Ein- und Ausschlusskriterien entsprechen, werden Fragebogenpakete, bestehend aus den Instrumenten zur Erfassung von Schlafproblemen, der subjektiven Schlafqualität und den krankenhausspezifischen Faktoren sowie den Einzelitems zu Alter und Geschlecht

ausgehändigt. Den an der Befragung teilnehmenden (stellvertretenden) Stationsleitungen wird ein Fragebogenpaket übergeben, welches aus dem Instrument zur Identifikation von Maßnahmen und Strategien zur Schlafförderung sowie den Einzelitems zu den soziodemographischen Merkmalen der eigenen Person besteht. Daneben erhalten alle Teilnehmenden von den Kontaktpersonen jeweils eine schriftliche Studieninformation, eine Einwilligungserklärung sowie einen Umschlag. Die Teilnehmenden werden dazu aufgefordert, die ausgefüllten Studienmaterialien in den Umschlag zu legen und diesen zu verschließen. Alle Patient*innen, denen zuvor die Studienunterlagen ausgehändigt wurden, werden im weiteren Verlauf einmalig durch die Kontaktpersonen mündlich an die Studienteilnahme erinnert. Die verschlossenen Umschläge werden den Kontaktpersonen noch am selben oder am folgenden Tag ausgehändigt und anschließend den Forschenden übergeben.

Auf den Umschlägen befinden sich Codenummern, die eine Zuordnung des Studienmaterials zum Krankenhaus (Stelle 1 und 2) sowie zur Station (Stelle 3 und 4) erlauben. Eine fünfte und sechste Stelle des Codes auf den Umschlägen der Patient*innen dienen zudem der temporären Identifikation der befragten Personen. Hierfür wird auf den teilnehmenden Stationen eine Codeliste geführt, anhand derer Codenummern und befragte Personen einander zugeordnet werden können. Die Codeliste dient zum einen dem Zweck, dass ein separates und ebenfalls mit einem Code versehenes Fragebogenblatt zu den klinischen Merkmalen der teilnehmenden Patient*innen von den Kontaktpersonen unter Durchsicht der Krankenakte ausgefüllt und im Anschluss vom Forschungsteam dem übrigen Studienmaterial zugeordnet werden kann. Zum anderen bietet die Codeliste für die Kontaktpersonen die Möglichkeit, die Verteilung und Einsammlung der Fragebögen anhand eines Protokolls zu dokumentieren. Dem Studienteam ist die Codeliste zu keinem Zeitpunkt bekannt. Den Kontaktpersonen vor Ort sind die ausgefüllten Fragebögen, welche in den verschlossenen Umschlägen verwahrt sind, nicht zugänglich. Die Kontaktpersonen werden zudem dazu aufgefordert, die Codeliste unmittelbar nach Abschluss der Datenerhebung zu vernichten. Ab diesem Zeitpunkt ist eine Zuordnung des Studienmaterials lediglich zu Einrichtung und Station möglich. Die entsprechende Codeliste ist ausschließlich dem Studienteam zugänglich.

Datenmanagement

Nach Eintreffen der Fragebogenpakete im Studienzentrum werden die Umschläge vernichtet. Die Codenummern werden zur nachfolgenden Datenauswertung sowie zur stationsspezifischen Ergebnisrückmeldung auf dem Fragebogenpaket notiert. Es folgt eine Überführung der Studiendaten in das Softwareprogramm SPSS durch die Forschenden. Währenddessen werden erste Plausibilitätskontrollen vorgenommen.

Die erhobenen Daten werden im Studienzentrum in anonymisierter (Patient*innen) bzw. pseudonymisierter Form (Stationsleitungen) gesichert. Identifizierende Angaben zu den beteiligten

Krankenhäusern und Stationen, welche einen Personenbezug zu den teilnehmenden Stationsleitungen ermöglichen, werden dabei getrennt von dem übrigen Studienmaterial aufbewahrt. Die weitere Datenverarbeitung erfolgt in der geschützten räumlichen bzw. IT-gestützten Umgebung des Studienzentrums.

Datenanalyse

Die Daten aus den Befragungen werden zunächst deskriptiv analysiert. Kategoriale Variablen werden in absoluten und relativen Häufigkeiten dargestellt. Die Angabe der übrigen Variablen erfolgt anhand der relevanten Lage- und Streuungsparameter, entsprechend des jeweiligen Skalenniveaus. Darüber hinaus werden die Daten mittels unterschiedlicher inferenzstatistischer Verfahren analysiert (z. B. gepaarter t-Test bzw. McNemar-Test für einen Vergleich des Schlafs vor und während des Krankenhausaufenthalts sowie Regressionsanalysen für eine Untersuchung der Zusammenhänge). Hierbei werden mögliche Cluster-Effekte berücksichtigt. Die gesamte Datenanalyse erfolgt in der Statistiksoftware SPSS.

Offen formulierte Fragen sowie Freitextfelder in den Fragebögen werden mittels qualitativer Inhaltsanalyse (Mayring, 2010) ausgewertet. Die Kategorienbildung erfolgt durch Kombination einer deduktiven und induktiven Vorgehensweise.

TEIL II: AKZEPTANZ UND WIRKSAMKEIT VERSCHIEDENER INFORMATIONSSCHREIBEN ZUR REKRUTIERUNG VON PATIENT*INNEN IM KRANKENHAUS

Hintergrund

Eine wesentliche Komponente im empirischen Forschungsprozess ist die Rekrutierung von Teilnehmenden. Der Begriff *Rekrutierung* meint in diesem Zusammenhang die Identifizierung potentieller Forschungsteilnehmender, die Sicherstellung einer angemessenen und/oder repräsentativen Stichprobe sowie die Bindung der Teilnehmenden bis zum Studienende unter zeitgleicher Beachtung ökonomischer und ethischer Gesichtspunkte (Blanton, Morris, Prettyman, McCulloch, Redmond, Light & Wolf, 2006). Forschenden stehen hierfür eine Vielzahl unterschiedlicher Rekrutierungsstrategien, -techniken und -verfahren zur Verfügung (Heerman et al., 2017; Ngune, Jiwa, Dadich, Lotriet & Sriram, 2012).

Die Rekrutierung von Teilnehmenden wird häufig als Hürde für die Durchführung wissenschaftlicher Studien beschrieben. Hierzu veröffentlichte Übersichtsarbeiten zeigen, dass in einer Mehrheit der empirischen Studien, die ursprünglich angestrebte Stichprobengröße nicht oder nicht im vorgesehenen Zeitfenster erreicht werden konnte (Bower, Wilson & Mathers, 2007; McDonald et al., 2006). Bleibt die Sicherstellung einer angemessenen Stichprobe aus, nimmt dies in vielerlei Hinsicht Einfluss auf das Gelingen einer Studie, wie etwa auf die Einhaltung des zeitlichen und finanziellen Rahmens, die interne und externe Validität sowie die Dissemination der Forschungsergebnisse (Bower, Brueton, Gamble, Treweek, Smith, Young & Williamson, 2014; Ngune et al., 2012; Treweek et al., 2013). Zeitgleich sind empirische Daten, die den Prozess der Rekrutierung in Studien leiten könnten, zum aktuellen Zeitpunkt sehr begrenzt. Bisherige Einblicke stammen vornehmlich aus qualitativen Fallstudien. Diese liefern erste Hinweise dafür, dass eine erfolgreiche Gewinnung von Teilnehmenden von vielerlei Faktoren in unterschiedlichen Rekrutierungselementen beeinflusst wird (Bower et al., 2014).

Ein Element in der Phase der Rekrutierung ist die Vermittlung von Studieninformationen (Bower et al., 2014). Während es für die Einholung der informierten Einwilligung üblich ist, auf mündliche und schriftliche Studieninformationen zurückzugreifen, um potentielle Studienteilnehmende angemessen über Ziele, methodisches Vorgehen, erwartete Ergebnisse sowie potentielle Risiken zu informieren, wurde der Einsatz gebräuchlicher Studieninformationsschreiben als eine potentielle Barriere für deren Rekrutierung identifiziert. Schriftliche Studieninformationen werden aufgrund ihres Umfangs und der Komplexität ihrer Inhalte häufig als Belastung beschrieben (Antoniou, Draper, Reed, Burls, Southwood & Zeegers, 2011; Bower et al., 2014). Aus diesen Erkenntnissen wurden erste Empfehlungen

hinsichtlich einer zielgruppenspezifischen Gestaltung und eines für das jeweilige Forschungsvorhaben angemessenen Umfangs der Studieninformationen abgeleitet (Bower et al., 2014). Empirische Untersuchungen, die auf diesen ersten Empfehlungen aufbauen und konkret unterschiedliche Rekrutierungsansätze hinsichtlich ihrer Akzeptanz und Wirksamkeit untersuchen, fehlen allerdings bisher. Ausgehend von der Annahme, dass Personengruppen verschieden auf Rekrutierungsansätze reagieren, fehlen ferner Orientierungshilfen hinsichtlich der von einem konkreten Ansatz zu erwartenden soziodemographischen Vielfalt der Stichprobe. Diese Informationen sind besonders relevant, um in einer Stichprobe, ein breites Spektrum von Forschungsteilnehmenden abbilden oder gezielt bestimmte Personengruppen in die Forschung einbinden zu können (Gaertner, Seitz, Fuchs, Busch, Holzhausen, Martus & Scheidt-Nave, 2016; Galea & Tracy, 2007). Inwiefern die Gestaltung der Studieninformation mit einer Merkmalsvarianz der Teilnehmenden einhergeht, ist bisher jedoch ebenfalls unbekannt.

Ziele

Obgleich die Sicherstellung einer angemessenen Stichprobe eine bedeutsame Rolle im Forschungsprozess einnimmt, sind die bisherigen Erkenntnisse zur Rekrutierung von Teilnehmenden begrenzt. Ausgehend von dem bisherigen Stand der Forschung sollen daher zwei Rekrutierungsansätze, die sich in der Gestaltung der schriftlichen Studieninformation unterscheiden, untersucht werden. Hierfür wird in dem vorliegenden Forschungsvorhaben die Zielsetzung verfolgt, Akzeptanz und Wirksamkeit eines ansprechend gestalteten Studieninformationsflyers im Vergleich zu einem formalen Studieninformationsschreiben zur Rekrutierung von Teilnehmenden in einer Fragebogenstudie zum Schlaf im Krankenhaus zu untersuchen.

Es wird erwartet, dass die Durchführung der Studie die Ermittlung der Akzeptanz und Wirksamkeit zweier schriftlicher Studieninformationen ermöglicht. Die Erkenntnisse dienen der Ableitung konkreter Empfehlungen für die Gestaltung einer Komponente des empirischen Forschungsprozesses und damit der erfolgreichen Generierung sowie Verbreitung wissenschaftlicher Erkenntnisse dar.

Methodik

Design

Zum Vergleich zweier schriftlicher Studieninformationen wird eine eingebettete Cluster-randomisierte Studie (SWAT) als Bestandteil der in Teil I des vorliegenden Studienprotokolls abgebildeten querschnittlichen Beobachtungsstudie zum Schlaf hospitalisierter Patient*innen durchgeführt. Hierfür

werden Krankenhauspatient*innen aus zufällig ausgewählten Krankenhäusern rund um das Studienzentrum anhand einer Cluster-Randomisierung zwei Studiengruppen zugeteilt, die sich hinsichtlich des verwendeten schriftlichen Studieninformationsmaterials unterscheiden. Sammlung und Auswertung der Daten zur Akzeptanz und Wirksamkeit beider Rekrutierungsansätze erfolgen anhand qualitativer und quantitativer Methoden.

Stichprobe

Die Stichprobe besteht aus den Einrichtungen, Stationen und Patient*innen, die sich zu einer Teilnahme an dem in Teil I beschriebenen Forschungsvorhaben bereiterklären.

Für den zweiten Teil des Forschungsvorhabens wird eine Cluster-Randomisierung vorgenommen, anhand derer zwei Studiengruppen gebildet werden. Ein Cluster ist definiert als einzelne teilnehmende Station innerhalb der Krankenhäuser mit allen dort im Datenerhebungszeitraum stationär aufgenommen sowie die Ein- und Ausschlusskriterien erfüllenden Patient*innen. Die Randomisierung erfolgt durch eine externe Person anhand computergenerierter Randomisierungslisten, stratifiziert nach Krankenhaus, sodass für jede Einrichtung eine separate Randomisierungsliste erstellt wird.

Rekrutierung

Informationen zur Rekrutierung der Einrichtungen und Stationen sind dem ersten Teil des vorliegenden Studienprotokolls (S. 3ff.) zu entnehmen.

Für die Rekrutierung der Patient*innen agieren die Forschenden über vermittelnde Kontaktpersonen (i. d. R. Stationsleitungen) auf den teilnehmenden Stationen. Diese sprechen alle Patient*innen, die den Ein- und Ausschlusskriterien entsprechen, persönlich an und übermitteln in einem Gespräch erste Studieninformationen. Hierauf werden die Kontaktpersonen im Vorfeld durch die Forschenden vorbereitet. Bei Bekundung des Interesses an einer Studienteilnahme, werden den Patient*innen die Studienunterlagen ausgehändigt. Je nach Gruppenzugehörigkeit unterscheidet sich die Gestaltung der darin enthaltenen schriftlichen Studieninformation. Während die Patient*innen in der einen Gruppe ein klassisches, mehrseitiges Informationsschreiben (Anhang 7) erhalten, wird den Patient*innen in der anderen Gruppe ein Informationsflyer zur Studie (Anhang 8) ausgehändigt.

Die Gestaltung der schriftlichen Studieninformationen orientiert sich an verfügbaren Empfehlungen. In dem klassischen Informationsschreiben werden in einem ersten Teil zunächst allgemeine Informationen über die wesentlichen Elemente der Studie präsentiert (Hintergrund und Ziele, Abläufe sowie Nutzen und Risiken), während ein zweiter Teil der Darstellung ausführlicher Informationen zum Datenschutz dient (National Research Ethics Service, 2009). In dem Flyer werden die Studieninformationen in ihrer Länge und Detailliertheit reduziert und ansprechend dargestellt

(Antoniou et al., 2011). Zur Integration eines weiteren Kommunikationskanals (Huang, Bull, Johnston McKee, Mahon, Harper, Roberts & Team, 2018), enthält der Flyer neben den schriftlichen Informationen zudem einen QR-Code bzw. einen Link, welche zu einer Videoaufnahme führen. In dieser stellen die Forschenden sich und das geplante Forschungsvorhaben vor. Für beide Studieninformationen wird außerdem auf eine einfache, leicht verständliche Sprache zurückgegriffen (Bower et al., 2014).

Datenerhebung

Zur Beurteilung der Akzeptanz der ausgehändigten schriftlichen Studieninformation werden die teilnehmenden Patient*innen um das Ausfüllen eines eigens entwickelten Fragebogens (Anhang 9) gebeten. Dieser enthält zum einen geschlossene Fragen, anhand derer vorab festgelegte Bereiche, wie Verständlichkeit, Vollständigkeit oder Angemessenheit des Umfangs der Studieninformation, mittels numerischer Ratingskala bewertet werden. Zum anderen umfasst der Fragebogen offen gestellte Frage, welche auf die Identifizierung gelungener bzw. weniger gelungener Elemente sowie das Erkennen von Verbesserungspotentialen zielen.

Der Fragebogen wurde literaturgestützt im Forschungsteam entwickelt. Vor dessen Einsatz in der geplanten Datenerhebung erfolgt ein Pretest. Hierfür werden fünf Personen, die den Ein- und Ausschlusskriterien entsprechen, sowie fünf Fachexpert*innen rekrutiert. Bei Bedarf werden Anpassungen des entwickelten Fragebogens vorgenommen.

Für die Untersuchung der Rekrutierungsansätze werden zudem, abseits der für Teil I des Studienprotokolls zu erhebenden Merkmale Alter und Geschlecht, weitere soziodemographische Daten der Teilnehmenden gesammelt. Erfasst werden der sozioökonomische Status (Bildungsniveau in Form des höchsten Schulabschlusses und Berufsstatus) sowie der kulturelle Hintergrund (Staatsangehörigkeit, Geburtsland und Muttersprache).

Der selbst entwickelte Fragebogen und entsprechende Items zu den Personenmerkmalen der Teilnehmenden sind Teil des ausgehändigten Fragebogenpakets.

Daneben werden in dem während der Datenerhebung von den Kontaktpersonen auf den Stationen geführten Protokoll, Gründe für eine Nicht-Teilnahme der Patient*innen an der Studie erfasst und von den Forschenden Informationen zur Häufigkeit der Videoaufrufe gesammelt.

Datenanalyse

Für beide Rekrutierungsansätze wird die Rücklaufquote (Anzahl der Patient*innen, die an dem Forschungsvorhaben teilnehmen/Anzahl der Patient*innen, die für eine Forschungsteilnahme angefragt wurden) berechnet. Darüber hinaus erfolgt, unter Angabe von Häufigkeiten sowie Lage- und

Streuungsparametern, eine deskriptive Analyse der soziodemographischen Merkmale der Teilnehmenden in beiden Studiengruppen. Zusätzlich werden die Daten mittels unterschiedlicher inferenzstatistischer Analysen ausgewertet (z. B. Vergleich der soziodemographischen Merkmalsverteilung durch Chi-Quadrat-Test bzw. ANOVA). Die Datenauswertung erfolgt in der Statistiksoftware SPSS. Offene Fragen und Freitextfelder in dem Fragebogen werden zudem anhand der qualitativen Inhaltsanalyse (Mayring, 2010) ausgewertet. An der Datenauswertung beteiligte Personen werden in Bezug auf die Cluster-Gruppenzugehörigkeit verblindet.

TEIL III: ENTSCHEIDUNGSFINDUNG VON PFLEGEFACHPERSONEN ZUR VERIFIKATION DER KORREKTEN LAGE BLIND APPLIZIERTER MAGENSONDEN IM KRANKENHAUS

Hintergrund

Die Anlage von Magensonden wird vielfach nach ärztlicher Anordnung an dafür qualifizierte Pflegefachpersonen delegiert. Ausgewählte Case Reports verweisen auf aufgetretene Komplikationen durch unsachgemäße Anwendung bei der Anlage. So werden Fehllagen wie die tracheale anstatt der ösophagialen Intubation (Xu & Li, 2011), massive Blutungen durch traumatisierte nasale Mucosa (Smith, Santa Ana, Fordtran & Guileyardo, 2018), die Perforation des Ösophagus (Isik, Firat, Peker, Sayar, Idiz & Soytürk, 2014) oder die Entstehung eines Pneumothorax (Al Saif, Hammodi, Al-Azem & Al-Hubail, 2015) berichtet. Ausgehend von den gravierenden Risiken, die mit der Anlage nasogastraler Sonden einhergehen, erhält die Verifikation der korrekten Sondenlage eine große Bedeutung.

Im Rahmen einer Literaturübersicht hinsichtlich pulmonaler Komplikationen nach Anlagen von 9931 blind platzierten nasoenteralen Sonden wurden 187 fehlplatziert im Tracheobronchialbaum (1,9%). Von diesen 187 Fehlplatzierungen waren 35 berichtete Pneumothoraces (18,7%) (Sparks, Chase, Coughlin & Perry, 2011). International durch Fachgesellschaften publizierte Leitlinien und Empfehlungen zur Verifikation der korrekten Lage nasogastral applizierter Magensonden verweisen auf die Güte unterschiedlicher Methoden. So empfehlen gemäß einer Literaturübersicht die Autor*innen in 11 von 14 internationalen Leitlinien die radiologische Überprüfung (Range von „immer erforderlich“ bis „erst wenn andere Methoden versagen“), wobei hierbei geographische Divergenzen zu erkennen sind (Metheny, Krieger, Healey & Meert, 2019). Zum Beispiel favorisieren eine Mehrheit US-amerikanischer Guidelines die radiologische Bestätigung der Sondenlokalisierung als „first-line“ Methode. Leitlinien aus Europa und Australien verweisen hingegen auf die radiologische Überprüfung, wenn die Methode mittels Messung des pH-Werts versagt oder gewisse Risiken bestehen. Von den nichtradiologischen Methoden wird die pH-Wert-Messung am meisten, die auskultatorische Methode hingegen am wenigsten favorisiert (Metheny et al., 2019). Für Deutschland liegt derzeit keine evidenzbasierte Empfehlung vor. Betrachtet man das beschriebene Vorgehen zur Verifikation der Lage nasogastraler Sonden in einem gebräuchlichen Pflegelehrbuch, so werden die Aspiration von Magensekret inkl. der Messung des pH-Werts sowie die Methode der Auskultation empfohlen. Zu einer Röntgenkontrolle wird erst bei Zweifelsfällen geraten (Pflege heute, 2014).

Ausgewählte internationale Befragungsstudien verweisen hinsichtlich der Verifikation der Lage nasogastraler Sonden auf Differenzen zwischen den Empfehlungen von Fachgesellschaften und der eigentlichen Durchführung in der Praxis. Eine Befragung von 2298 Pflegefachpersonen US-

amerikanischer Intensivstationen macht deutlich, dass die dortige Empfehlung der radiologischen Kontrolle vor der ersten Nutzung von Magensonden, nicht adäquat implementiert zu sein scheint. Weniger als 60% der Befragten berichteten, dass eine radiologische Kontrolle vor der ersten Nutzung blind applizierter Magensonden ohne Führungsdraht durchgeführt wird. Auch findet die auskultatorische Methode weitverbreitete Anwendung, obwohl Empfehlungen von Fachgesellschaften davon abraten (Metheny, Stewart & Mills, 2012). Auch eine kürzlich publizierte Befragung von 464 klinisch tätigen Pflegefachpersonen in China kommt zu dem Ergebnis, dass das praktische Vorgehen vieler der Befragten nicht konsistent ist mit internationalen Leitlinien. So werden traditionelle Methoden zur Bestätigung der Magensondenlage, wie die Auskultation injizierter Luft, der Luftblasentest oder die Beobachtung des Aspirats, als Goldstandard angesehen (Xu, Huang, Lin, Zheng & Zhu, 2020).

Die Diskrepanz zwischen internationalen Leitlinienempfehlungen und der eigentlichen Durchführung in der Praxis verdeutlicht die Notwendigkeit der Auseinandersetzung mit dem Thema auch aus Sicht der pflegerischen Praxis in deutschen Intensivstationen.

Ziele

Die Anlage nasogastraler Sonden gilt in Deutschland als ärztlich delegierte Tätigkeit von Pflegefachpersonen in unterschiedlichen Krankenhaussettings. Um die Entscheidungsfindung von deutschen Intensivpflegefachpersonen bzgl. der Verifikation der korrekten Magensondenlage zu untersuchen, ist eine multizentrische Befragungsstudie mit explorativem Charakter vor dem Hintergrund folgender Forschungsfragen geplant:

1. Welche diagnostischen Methoden setzen die Pflegefachpersonen zur Verifikation der korrekten Lage einer Magensonde ein?
2. Entspricht das Vorgehen zur Verifikation der in Deutschland sowie international verfügbaren best-practice oder evidenzbasierten Kriterien?
3. Wie schätzen die Pflegefachpersonen die Eignung bekannter Methoden zur Verifikation ein?

Methodik

Design

Zur Beantwortung der Forschungsfragen ist eine querschnittliche multizentrische Beobachtungsstudie in einer repräsentativen Auswahl von Krankenhäusern im Umkreis von 50 km um das Studienzentrum

geplant. Der Feldzugang erfolgt über das in Teil I des vorliegenden Studienprotokolls beschriebene Forschungsvorhaben. Befragt werden Stationsleitungen, Praxisanleiter*innen und/oder Pflegefachpersonen mit schichtleitender Funktion aller Intensivstationen der rund 30 eingeschlossenen Einrichtungen. Datenerhebung und -auswertung folgen einem Ansatz aus qualitativen und quantitativen Methoden.

Stichprobe und Rekrutierung

Innerhalb der durch stratifizierte Zufallsauswahl bestimmten rund 30 Einrichtungen, die sich zu einer Teilnahme an dem in Teil I des vorliegenden Studienprotokolls beschriebenen Forschungsvorhaben bereiterklären, ist ein Einschluss aller Intensivstationen vorgesehen. Derzeit ist von einer Populationsgröße von etwa 100 Stationen auszugehen. Auf jeder der Intensivstationen soll jeweils eine Pflegefachperson für eine Studienteilnahme gewonnen werden.

Die Ein- und Ausschlusskriterien für Stationen und Pflegefachpersonen sind Tabelle 2 zu entnehmen.

Tabelle 2: Ein- und Ausschlusskriterien

Ebene	Einschlusskriterien	Ausschlusskriterien
**Stationen**	▪ Interdisziplinäre oder fachspezifische Überwachungs- oder Intensivstationen	▪ Kinderintensivstationen
**Pflegefachpersonen**	▪ (Stellvertretende) Stationsleitung, Praxisanleiter*in mit entsprechender Fachweiterbildung oder Pflegefachperson mit zum Befragungszeitpunkt schichtleitender Funktion<br>▪ Stellenumfang von ≥ 50 Prozent der regelmäßigen wöchentlichen Arbeitszeit<br>▪ Vorliegen der Informierten Einwilligung für eine Studienteilnahme	

Der Zugang zu den Intensivstationen erfolgt über die Pflegedirektion des jeweiligen Krankenhauses. Diese übermittelt den Stationsleitungen bzw. Praxisanleiter*innen erste Informationen über die Studie und stellt bei Interesse einen Kontakt zu den Forschenden her. Im weiteren Verlauf händigen die Forschenden den potentiellen Studienteilnehmenden weiterführende Studieninformationen aus und holen, bei Fortbestehen des Interesses an einer Studienteilnahme, eine mündliche Einwilligung ein.

Datenerhebung

Die Befragung von Stationsleitungen, Praxisanleiter*innen und/oder Pflegefachpersonen mit schichtleitender Funktion erfolgt papier-basiert mittels Fragebogen.

Instrument

Zur Untersuchung der Entscheidungsfindung hinsichtlich der Verifikation der korrekten Magensondenlage wurde literaturgestützt ein Fragebogen (Anhang 11) entwickelt. Hierzu wurde eine Literaturrecherche hinsichtlich international vorhandener Leitlinien oder Empfehlungen sowie Befragungen zum Vorgehen bei der Verifikation nasogastraler Sonden durchgeführt. Eine erste Konzeptualisierung wurde auf dieser Basis vorgenommen und im Forschungsteam diskutiert. Die abgeleitete und innerhalb des Forschungsteams mehrfach überarbeitete Fragebogenversion wird im Vorfeld der Datenerhebung in Form eines Pretests überprüft. Hierfür werden fünf Pflegefachpersonen, welche praktische Erfahrungen hinsichtlich des Untersuchungsgegenstands aufweisen, aus dem beruflichen Netzwerk der Forschenden rekrutiert. Bei Bedarf erfolgt eine Überarbeitung des entwickelten Fragebogens.

Die aktuelle Version des Fragebogens besteht aus 15 Fragen, wovon fünf den beruflichen Werdegang, die Fachdisziplin sowie den beruflichen Status auf der jeweiligen Intensivstation erfragen (Fragen 1 bis 5). Die Häufigkeit (Frage 6) und Zuständigkeit (Frage 7) der Applikation von Magensonden sowie die Eignung von unterschiedlichen Methoden zur Verifikation wird in jeweils zwei Fragen thematisiert (Fragen 11, 12). Drei Fragen mit offenem Antwortformat sollen die Teilnehmenden auffordern, Methoden zur Bestimmung der internen Länge sowie der Verifikation der korrekten Lage, die auf der Station eingesetzt werden, zu beschreiben. Außerdem wird ein Fallbeispiel dargestellt, wozu die Teilnehmenden ihre Entscheidungsfindung stichwortartig darstellen müssen (Fragen 8 bis 10). Jeweils eine Frage thematisiert das Intervall (Frage 13) sowie den Zeitpunkt der Überprüfung der Magensonde (Frage 14). Welche Informationen nach der Anlage dokumentiert werden, wird in einer Frage angesprochen (Frage 15).

Vorgehensweise

An dem Tag, an dem die Forschenden in den Einrichtungen für die Datenerhebung der Studie „Sleep Acute“ (siehe Teil I des vorliegenden Studienprotokolls) vor Ort sind, werden die Studiendokumente den teilnehmenden Pflegefachpersonen entweder persönlich ausgehändigt oder auf den Stationen für sie hinterlegt. Übergeben werden der entwickelte Fragebogen sowie ein frankierter Rückumschlag. Vorgesehen ist, dass die zum Zeitpunkt diensthabende Stationsleitung (oder deren Vertretung) oder der/die Praxisanleiter*in teilnimmt. Alternativ wird die zum Befragungszeitpunkt diensthabende Schichtleitung zur Teilnahme gebeten.

Nach Möglichkeit werden die Fragebögen noch am selben Tag von den Pflegefachpersonen ausgefüllt und den Forschenden persönlich übergeben. Ist dies nicht realisierbar, besteht die Möglichkeit einer späteren postalischen Übersendung der Studiendokumente an die Forschenden durch Nutzung des frankierten Rückumschlags.

Datenmanagement

Nach Eintreffen der Studiendokumente im Studienzentrum werden die Daten durch die Forschenden in SPSS überführt. Hierbei erfolgen erste Plausibilitätskontrollen.

Die Sicherung und weitere Verarbeitung der anonymisiert erhobenen Daten findet in der geschützten räumlichen bzw. IT-gestützten Umgebung des Studienzentrums statt.

Datenanalyse

Die im Rahmen der Befragung erhobenen Daten werden deskriptiv analysiert. Offene Fragen werden anhand der qualitativen Inhaltsanalyse nach Mayring (Mayring, 2010) ausgewertet.

ETHISCHE ÜBERLEGUNGEN

Aufklärung und Einwilligung

Ziele und Inhalte der Forschungsvorhaben werden zunächst den Pflegedirektionen der ausgewählten Krankenhäuser vorgestellt. Diese stellen einen Zugang zu den beteiligten Stationen und den jeweiligen Kontaktpersonen (Teil I und II) bzw. teilnehmenden Pflegefachpersonen (Teil III) her. Letztere beiden werden durch das Forschungsteam ebenfalls über das Forschungsvorhaben informiert. Für Teil I und II des vorliegenden Studienprotokolls übernehmen im weiteren Verlauf die Kontaktpersonen auf den Stationen Auswahl und Aufklärung der Patient*innen. Darüber hinaus werden die Kontaktpersonen darum gebeten, bei eigenem Interesse an einer Studienteilnahme bzw. dem von Kolleg*innen, Kontakt zum Forschungsteam aufzunehmen.

Allen Beteiligten werden mündliche und schriftliche Informationen zur Studie ausgehändigt. Im Rahmen der informierten Einwilligung wird darauf hingewiesen, dass eine Teilnahme an dem Forschungsvorhaben freiwillig erfolgt und diese jederzeit und ohne die Angabe von Gründen abgelehnt bzw. widerrufen werden kann, ohne dass hieraus Nachteile entstehen. Nach der Informationsgabe wird den Patient*innen und Pflegefachpersonen eine angemessene Bedenkzeit eingeräumt. Die auf den Informationsschreiben vermerkten Kontaktdaten des Forschungsteams stellen zudem sicher, dass die Möglichkeit einer Beratung bei offenen Fragen gegeben ist.

Bei bestehendem Interesse an einer Studienteilnahme, wird für die in Teil I und II des vorliegenden Studienprotokolls vorgestellten Forschungsvorhaben eine schriftliche Einwilligung eingeholt. Ein Exemplar der Einwilligungserklärung verbleibt bei den Teilnehmenden. Im Rahmen der in Teil III dargelegten anonymisierten Befragung der Pflegefachpersonen, bekunden die Teilnehmenden ihre informierte Einwilligung durch das Ausfüllen des Fragebogens.

Mögliche Risiken

Für eine Einschätzung möglicher Risiken, die mit einer Studienteilnahme verbunden sind, wird im Folgenden zwischen den Befragungen der Pflegefachpersonen und der Patient*innen unterschieden.

Pflegefachpersonen

Teil I und Teil III

Pflegefachpersonen stellen in ihrer Rolle als Studienteilnehmende grundsätzlich keine vulnerable Personengruppe dar. Dies begründet sich darin, dass in den geplanten Forschungsvorhaben keine

persönlichen oder sensiblen Informationen, sondern ausschließlich berufsalltägliches Erfahrungswissen erfragt werden. Die Studienteilnahme als solche bedeutet für die Pflegefachpersonen jedoch einen zeitlichen Mehraufwand, was eine potentielle Belastung, etwa in Form von Rollendruck, bedeuten kann. Hierbei ist allerdings zu betonen, dass die Befragungen lediglich begrenzte Zeit (einmalig rund 15 bis 20 Minuten) in Anspruch nehmen. Darüber hinaus werden die Befragungen jeweils in den Krankenhäusern durchgeführt, in denen die Teilnehmenden arbeiten, sodass Mehraufwand (z. B. durch Anfahrtswege) vermieden wird. Die Pflegefachpersonen können zudem individuell über ein für sie günstiges Zeitfenster zum Ausfüllen der Fragebögen entscheiden. Ferner besteht zu jeder Zeit die Möglichkeit, eine Studienteilnahme abzulehnen oder zu beenden. Um hierbei der Gefahr einer bewussten oder unbewussten Einflussnahme der jeweiligen Pflegedirektion vorzubeugen, werden die Pflegefachpersonen im Vorfeld darauf hingewiesen, dass ihre Vorgesetzten nicht personen- oder stationsbezogen über abgelehnte Studienteilnahmen unterrichtet werden.

Es besteht ebenso die Möglichkeit, dass sich die Studienteilnahme positiv auf die Pflegefachpersonen auswirkt. Ihre Einbindung in Forschungsvorhaben kann als Wertschätzung empfunden werden, da ihre Perspektive anerkannt und ihren berufsalltäglichen Erfahrungen Aufmerksamkeit geschenkt wird.

Krankenhauspatient*innen

Teil I

Krankenhauspatient*innen hingegen gelten grundsätzlich als vulnerabel. Ein Krankenhausaufenthalt stellt eine Ausnahmesituation dar und ist häufig mit einer existenziellen Bedrohung sowie einer Beeinträchtigung des Wohlbefindens verbunden. Die Studienteilnahme, in Form einer schriftlichen Selbstauskunft zu Schlafproblemen und subjektiver Schlafqualität, kann hier eine zusätzliche Belastung darstellen. Zum einen ist diese mit einem gewissen (Zeit-)Aufwand verbunden. Zum anderen werden die Patient*innen dazu aufgefordert, sich aktiv mit einem potentiellen Problem auseinanderzusetzen, welches die Gefahr birgt, eine ohnehin bedrohliche Situation weiter zu erschweren. Patient*innen, deren aktueller Zustand eine Studienteilnahme nicht erlaubt, werden jedoch von vornherein aus dem Forschungsvorhaben ausgeschlossen. Daneben haben sich die ausgewählten Instrumente in früheren Forschungsarbeiten bereits als geeignet für einen Einsatz im Akutkrankenhaus erwiesen (Krotsetis et al. 2017; Wesselius et al., 2018). Der strukturierte Aufbau und die Kürze (insgesamt rund 15 bis 20 Minuten) der Fragebögen dienen hierbei der Entlastung der teilnehmenden Patient*innen. Ferner kann der Zeitpunkt des Ausfüllens der Fragebögen selbst gewählt und flexibel gehandhabt werden, sodass in keinem Fall Störungen der Versorgungsstrukturen und -abläufe durch eine Studienteilnahme zu erwarten sind.

Zeitgleich kann eine Studienteilnahme der Entlastung und der Förderung des Wohlbefindens der Patient*innen dienen, da diese die Möglichkeit erhalten, über ihre Situation und mögliche Probleme zu berichten. Das Erfragen ihrer Perspektive kann zudem dazu führen, dass Patient*innen sich anerkannt und ernst genommen fühlen, was sich wiederum positiv auf die Zufriedenheit auswirkt.

Sollte es trotz der bisherigen Ausführungen wider Erwarten zu Belastungen der Patient*innen durch eine Studienteilnahme kommen, haben diese jederzeit die Möglichkeit, einzelne Fragen nicht zu beantworten, die Befragung zu unterbrechen oder abzubrechen. Da sich die Patient*innen in einem Abhängigkeitsverhältnis befinden, wird ihnen gegenüber im Vorfeld betont, dass sich ihre Teilnahme in keiner Weise auf die Versorgung auswirkt. Zur Sicherstellung dieser Tatsache, werden für die Datenerhebung verschlossene Umschläge verwendet. Zum einen ist für die Kontaktpersonen auf den Stationen beim Einsammeln der Fragebögen auf diese Weise nicht ersichtlich, ob das Studienmaterial ausgefüllt wurde. Zum anderen verhindern die Umschläge auch nach dem Einsammeln der Fragebögen, dass die enthaltenen Informationen zugänglich sind und bestimmten Personen zugeordnet werden können. Codeliste und Fragebögen werden auf diese Weise zu jeder Zeit getrennt voneinander aufbewahrt.

Übergeordnetes Ziel der Studie ist, Erkenntnisse zu den Themen Schlaf, Schlafprobleme und Schlafqualität von Patient*innen im Krankenhaus zu generieren. Dies ist unabdingbar, um die aktuelle Situation abbilden und in der Folge mögliche Bedarfe ableiten zu können. Werden in Anbetracht dessen, Nutzen und Schaden des Forschungsvorhabens betrachtet, überwiegt der nachhaltige Nutzen gegenüber der Gefahr, kurzfristigen Schaden bei den Studienteilnehmenden herbeizuführen, dem versucht wird vorzubeugen und auf den im Zweifelsfall adäquat reagiert wird.

Teil II

Es ist nicht davon auszugehen, dass die Studienteilnahme, in Form des Erhalts unterschiedlicher schriftlicher Studieninformationen, mit Risiken für die Teilnehmenden einhergeht. Sowohl das klassisch gestaltete Informationsschreiben als auch der Informationsflyer beinhalten alle relevanten Kerninformationen zum Forschungsvorhaben, sodass bei erteilter Einwilligung in jedem Fall von einer informierten Zustimmung der Teilnehmenden auszugehen ist. Sollten sich die potentiellen Teilnehmenden über das Forschungsvorhaben, trotz mündlicher und schriftlicher Informationsgabe, nicht hinreichend informiert fühlen, besteht ferner die Möglichkeit, sich erneut an die Kontaktpersonen auf den Stationen oder die Forschenden selbst zu wenden. Darüber hinaus werden die potentiellen Teilnehmenden, wie für Teil I bereits beschrieben, darauf hingewiesen, dass die Teilnahme zu jeder Zeit abgelehnt bzw. widerrufen werden kann, ohne, dass ihnen hieraus Nachteile entstehen.

Ausgehend von dem Forschungsziel, Akzeptanz und Wirksamkeit für den Forschungsprozess vielversprechender und für potentielle Studienteilnehmende weniger belastende Studieninformationen zu untersuchen, ist ein nachhaltiger Nutzen des geplanten Forschungsvorhabens anzunehmen.

Datenschutz

Über den gesamten Studienverlauf werden die aktuell geltenden datenschutzrechtlichen Bestimmungen der Europäischen Datenschutz-Grundverordnung (DS-GVO) gemäß Art. 6 Abs. 1 lit. a, 7 und 9 Abs. 2 lit. a sowie des Bundesdatenschutzgesetztes (BDSG) strikt eingehalten.

Entsprechend DS-GVO, Art. 4 Abs. 1 und Art. 9 Abs. 1, werden in den in Teil I und II vorgestellten Forschungsvorhaben personenbezogene Daten bzw. besondere Kategorien personenbezogener Daten erhoben, verarbeitet sowie genutzt. Diese gelten als ein besonderes Schutzgut. Die Teilnahme an den Forschungsvorhaben setzt daher für Patient*innen und Pflegefachpersonen die informierte Einwilligung in Schriftform voraus, welche sich ausdrücklich auf zuvor genannte Datenarten bezieht. Ausschließlich bei Vorliegen dieser, wird die beschriebene Datenerhebung durchgeführt. Sollte die Einwilligung entzogen werden, ist eine Studienteilnahme nicht länger möglich. Bis zur Vernichtung der Codelisten, welche identifizierende Daten und Pseudonyme einander zuordnen, haben die Teilnehmenden die Möglichkeit, ihre zuvor erteilte Einwilligung zu widerrufen. In Teil III des vorliegenden Studienprotokolls werden die Daten hingegen anonymisiert erhoben. Ein Personenbezug ist daher nicht gegeben.

Die Datenerhebung und -auswertung unterliegen dem Prinzip strenger Vertraulichkeit, die den Teilnehmenden vor Studienbeginn zugesichert wird. Alle Daten werden ausschließlich für Zwecke der wissenschaftlichen Forschung und entsprechend der informierten Einwilligung verwendet. Die Beteiligten werden darüber informiert, dass die in der Studie gewonnenen Daten im Rahmen wissenschaftlicher Publikationen veröffentlicht werden sollen. Hierbei werden die gewonnenen Daten so aufbereitet, dass Rückschlüsse auf einzelne Personen und Einrichtungen unmöglich sind. Der Einwilligung entsprechend, ist es den Forschenden zudem vorbehalten, die erhobenen Daten in weiteren Forschungsarbeiten, wie Sekundärdatenanalysen, erneut zu verwenden.

Bis zum Abschluss der Datenerhebung und der persönlichen Übergabe der Studienunterlagen an die Forschenden, werden die Fragebögen und die Codelisten durch die Kontaktperson für andere Personen unzugänglich sowie getrennt voneinander auf den teilnehmenden Stationen in abschließbaren Schränken gelagert. Im Anschluss erfolgt die sichere Aufbewahrung aller in dem

Rahmen des Forschungsvorhabens erhobenen Daten in der geschützten IT-Umgebung (elektronische Daten) bzw. abschließbaren Schränken (papiergestützte Daten) des Instituts für Pflegewissenschaft der Universität zu Köln. Diese sind ausschließlich den Projektmitarbeitenden zugänglich. Die im Rahmen von Teil I und II erhobenen Patient*innendaten sowie die im Rahmen von Teil III erhobenen Daten der Pflegefachpersonen liegen den Forschenden dabei ausschließlich in anonymisierter Form vor. Die Daten aus den Befragungen der Pflegefachpersonen in Teil I sind hingegen pseudonymisiert. Identifizierende Angaben zu den beteiligten Krankenhäusern und Stationen, welche Rückschlüsse auf die teilnehmenden Stationsleitungen ermöglichen, werden dabei getrennt von dem übrigen Studienmaterial aufbewahrt. Nach Abschluss der Datenauswertung werden die Daten, durch Vernichtung der entsprechenden Codeliste, anonymisiert.

Die gesammelten Daten werden über einen Zeitraum von zehn Jahren durch die Universität zu Köln aufbewahrt und anschließend gelöscht.

LITERATURVERZEICHNIS

Al Saif, N., Hammodi, A., Al-Azem, M. A. & Al-Hubail, R. (2015). Tension Pneumothorax and Subcutaneous Emphysema Complicating Insertion of Nasogastric Tube. *Case Rep Crit Care, 2015*, 690742.

Antoniou, E., Draper, H., Reed, K., Burls, A., Southwood, T. & Zeegers, M. (2011). An empirical study on the preferred size of the participant information sheet in research. *J Med Ethics, 37*, 557-562.

Beaton, D. E., Bombardier, C., Guillemin, F. & Ferraz, M. B. (2000). Guidelines for the process of cross-cultural adaptation of self-report measures. *Spine (Phila Pa 1976), 25*(24), 3186-3191.

Blanton, S., Morris, D. M., Prettyman, M. G., McCulloch, K., Redmond, S., Light, K. E. & Wolf, S. L. (2006). Lessons learned in participant recruitment and retention: the EXCITE trial. *Phys Ther, 86*(11), 1520-1533.

Bower, P., Brueton, V., Gamble, C., Treweek, S., Smith, C. T., Young, B. & Williamson, P. (2014). Interventions to improve recruitment and retention in clinical trials: a survey and workshop to assess current practice and future priorities. *Trials, 15*, 399.

Bower, P., Wilson, S. & Mathers, N. (2007). Short report: How often do UK primary care trials face recruitment delays? *Family Practice, 24*(6), 601-603.

Buysse, D. J., Reynolds, C. F., Monk, T. H., Berman, S. R. & Kupfer, D. J. (1989). The Pittsburgh Sleep Quality Index: a new instrument for psychiatric practice and research. *Psychiatry Res, 28*(2), 193-213.

Buysse, D. J., Yu, L., Moul, D. E., Germain, A., Stover, A., Dodds, N. E., . . . Pilkonis, P. A. (2010). Development and validation of patient-reported outcome measures for sleep disturbance and sleep-related impairments. *Sleep, 33*(6), 781-792.

Carney, C. E., Buysse, D. J., Ancoli-Israel, S., Edinger, J. D., Krystal, A. D., Lichstein, K. L. & Morin, C. M. (2012). The consensus sleep diary: standardizing prospective sleep self-monitoring. *Sleep, 35*(2), 287-302.

Edwards, P. J., Roberts, I., Clarke, M. J., Diguiseppi, C., Wentz, R., Kwan, I., . . . Pratap, S. (2009). Methods to increase response to postal and electronic questionnaires. *Cochrane Database Syst Rev*(3), MR000008.

Eliassen, K. M. & Hopstock, L. A. (2011). Sleep promotion in the intensive care unit-a survey of nurses' interventions. *Intensive Crit Care Nurs, 27*(3), 138-142.

Friese, R. S. (2008). Sleep and recovery from critical illness and injury: a review of theory, current practice, and future directions. *Crit Care Med, 36*(3), 697-705.

Gaertner, B., Seitz, I., Fuchs, J., Busch, M. A., Holzhausen, M., Martus, P. & Scheidt-Nave, C. (2016). Baseline participation in a health examination survey of the population 65 years and older: who is missed and why? *BMC Geriatr, 16*, 21.

Galea, S. & Tracy, M. (2007). Participation rates in epidemiologic studies. *Ann Epidemiol, 17*(9), 643-653.

Ganz, F. D. (2012). Sleep and immune function. *Crit Care Nurse, 32*(2), e19-25.

Gellerstedt, L., Medin, J. & Rydell Karlsson, M. (2014). Patients’ experiences of sleep in hospital: A qualitative interview study. *J Res Nurs, 19*(3), 176-188.

Heerman, W. J., Jackson, N., Roumie, C. L., Harris, P. A., Rosenbloom, S. T., Pulley, J., . . . Kripalani, S. (2017). Recruitment methods for survey research: Findings from the Mid-South Clinical Data Research Network. *Contemp Clin Trials, 62*, 50-55.

Hoevenaar-Blom, M. P., Spijkerman, A. M., Kromhout, D. & Verschuren, W. M. (2014). Sufficient sleep duration contributes to lower cardiovascular disease risk in addition to four traditional lifestyle factors: the MORGEN study. *Eur J Prev Cardiol, 21*(11), 1367-1375.

Huang, G. D., Bull, J., Johnston McKee, K., Mahon, E., Harper, B., Roberts, J. N. & Team, C. R. P. (2018). Clinical trials recruitment planning: A proposed framework from the Clinical Trials Transformation Initiative. *Contemp Clin Trials, 66*, 74-79.

Isik, A., Firat, D., Peker, K., Sayar, I., Idiz, O. & Soytürk, M. (2014). A case report of esophageal perforation: Complication of nasogastric tube placement. *Am J Case Rep, 15*, 168-171.

John, M. E., Edet, O., Mgbekem, M., Robinson-Bassey, Duke, Esienumoh, E. & Ndebbio. (2007). Sleep disturbance among patients in hospital: implications for nursing care. *West African Journal of Nursing, 18*, 42-48.

Kauffmann, L., Heinemann, S., Himmel, W., Hußmann, O., Schlott, T. & Weiß, V. (2018). Nicht-medikamentöse Maßnahmen bei Ein- und Durchschlafproblemen von älteren Patienten im Krankenhaus – Qualitative Interviews mit Pflegenden. *Pflege, 31*, 1-10.

Krotsetis, S., Richards, K. C., Behncke, A. & Köpke, S. (2017). The reliability of the German version of the Richards Campbell Sleep Questionnaire. *Nurs Crit Care, 22*(4), 247-252.

Lane, T. & East, L. A. (2008). Sleep disruption experienced by surgical patients in an acute hospital. *Br J Nurs, 17*(12), 766-771.

Lange, T., Dimitrov, S. & Born, J. (2010). Effects of sleep and circadian rhythm on the human immune system. *Ann N Y Acad Sci, 1193*, 48-59.

Mayring, P. (2010). *Qualitative Inhaltsanalyse: Grundlagen und Techniken* (11 ed.). Weinheim: Beltz.

McDonald, A. M., Knight, R. C., Campbell, M. K., Entwistle, V. A., Grant, A. M., Cook, J. A., . . . Snowdon, C. (2006). What influences recruitment to randomised controlled trials? A review of trials funded by two UK funding agencies. *Trials, 7*, 9.

Metheny, N. A., Krieger, M. M., Healey, F. & Meert, K. L. (2019). A review of guidelines to distinguish between gastric and pulmonary placement of nasogastric tubes. *Heart Lung, 48*(3), 226-235.

Metheny, N. A., Stewart, B. J. & Mills, A. C. (2012). Blind insertion of feeding tubes in intensive care units: a national survey. *Am J Crit Care, 21*(5), 352-360.

National Research Ethics Service. (2009). *Information sheets and consent forms: guidance for researchers and reviewers*. Retrieved from http://www.nres.npsa.nhs.uk

Ngune, I., Jiwa, M., Dadich, A., Lotriet, J. & Sriram, D. (2012). Effective recruitment strategies in primary care research: a systematic review. *Qual Prim Care, 20*(2), 115-123.

Pellatt, G. C. (2007). The nurse's role in promoting a good night's sleep for patients. *Br J Nurs, 16*(10), 602-605.

Pflege heute: Lehrbuch für Pflegeberufe. (2014). (6., vollst. überarb. Aufl. ed.). München: Elsevier, Urban & Fischer.

Pilkington, S. (2013). Causes and consequences of sleep deprivation in hospitalised patients. *Nurs Stand, 27*(49), 35-42.

Radtke, K., Obermann, K. & Teymer, L. (2014). Nursing knowledge of physiological and psychological outcomes related to patient sleep deprivation in the acute care setting. *Medsurg Nurs, 23*(3), 178-184.

Rasch, B. & Born, J. (2013). About Sleep's Role in Memory. *Physiol Rev, 93*, 681-766.

Raymond, I., Nielsen, T. A., Lavigne, G., Manzini, C. & Choiniere, M. (2001). Quality of sleep and its daily relationship to pain intensity in hospitalized adult burn patients. *Pain, 92*(3), 381-388.

Redeker, N. S., Hedges, C. & Booker, K. J. (2011). Sleep in Adult Acute and Critical Care Settings. In N. S. Redeker & G. Phillips McEnany (Eds.), *Sleep Disorders and Sleep Promotion in Nursing Practice* (pp. 321-338). New York: Springer Publishing Company.

Richards K. (1987). Techniques for measurement of sleep in critical care. *Focus Crit Care*, *14*(4), 34-40.

Salzmann-Erikson, M., Lagerqvist, L. & Pousette, S. (2015). Keep calm and have a good night: nurses' strategies to promote inpatients' sleep in the hospital environment. *Scand J Caring Sci, 30*(2), 356-364.

Smith, A. L., Santa Ana, C. A., Fordtran, J. S. & Guileyardo, J. M. (2018). Deaths associated with insertion of nasogastric tubes for enteral nutrition in the medical intensive care unit: Clinical and autopsy findings. *Baylor University Medical Center Proceedings, 31*(3), 310-316.

Sparks, D. A., Chase, D. M., Coughlin, L. M. & Perry, E. (2011). Pulmonary complications of 9931 narrow-bore nasoenteric tubes during blind placement: a critical review. *JPEN J Parenter Enteral Nutr, 35*(5), 625-629.

Stone, K. L., Ensrud, K. E. & Ancoli-Israel, S. (2008). Sleep, insomnia and falls in elderly patients. *Sleep Med, 9*, 18-22.

Treweek, S., Lockhart, P., Pitkethly, M., Cook, J. A., Kjeldstrom, M., Johansen, M., . . . Mitchell, E. D. (2013). Methods to improve recruitment to randomised controlled trials: Cochrane systematic review and meta-analysis. *BMJ Open, 3*(2).

Weinhouse, G. L., Schwab, R. J., Watson, P. L., Patil, N., Vaccaro, B., Pandharipande, P. & Ely, E. W. (2009). Bench-to-bedside review: delirium in ICU patients - importance of sleep deprivation. *Crit Care, 13*(6), 234.

Wesselius, H. M., van den Ende, E. S., Alsma, J., Ter Maaten, J. C., Schuit, S. C. E., Stassen, P. M., . . . Onderzoeks Consortium Acute Geneeskunde" Acute Medicine Research, C. (2018). Quality and Quantity of Sleep and Factors Associated With Sleep Disturbance in Hospitalized Patients. *JAMA Intern Med, 178*(9), 1201-1208.

Xu, L. C., Huang, X. J., Lin, B. X., Zheng, J. Y. & Zhu, H. H. (2020). Clinical nurses' nasogastric feeding practices in adults: a multicenter cross-sectional survey in China. *J Int Med Res, 48*(4), 300060520920051.

Xu, Z. & Li, W. (2011). Aspiration pneumonia caused by inadvertent insertion of gastric tube in an obtunded patient postoperatively. *BMJ Case Rep, 2011*.

ANHANG

Anhang 1: Consensus Sleep Diary (CSD) – Kernversion

(Dieser Anhang enthält zusätzliche Details zur Host-Study – Teil 1)

Anhang 2: Consensus Sleep Diary (CSD) – Zusätzliche Items

(Dieser Anhang enthält zusätzliche Details zur Host-Study – Teil 1)

Anhang 3: Sleep Disturbance Item Bank – Short Form 8a

(Dieser Anhang enthält zusätzliche Details zur Host-Study – Teil 1)

Anhang 4: Richards-Campbell Sleep Questionnaire (RCSQ)

(Dieser Anhang enthält zusätzliche Details zur Host-Study – Teil 1)

Anhang 5: Fragen zu krankenhausspezifischen Faktoren

(Dieser Anhang enthält zusätzliche Details zur Host-Study – Teil 1)

Anhang 6: Fragebogen zur Identifikation derzeit angewendeter Strategien und Maßnahmen zur Schlafförderung

(Dieser Anhang enthält zusätzliche Details zur Host-Study – Teil 1)

Anhang 7: Informations- und Einwilligungsschreiben für Patient*innen (Teil I und II)

Sleep Acute

Wissenschaftliche Studie zur Untersuchung des Schlafs von Patientinnen und Patienten während der stationären Versorgung im Krankenhaus

Verantwortlich gemäß §4 Abs. 7 DS-GVO:

Institut für Pflegewissenschaft, Medizinische Fakultät, Universität zu Köln
Prof. Dr. phil. Sascha Köpke
Gleueler Straße 176-178
50935 Köln
Telefon: 0221 478 51658
E-Mail: sascha.koepke@uk-koeln.de

Informationsschreiben für Patientinnen und Patienten

Sehr geehrte Damen und Herren,

das vorliegende Schreiben dient dazu, Sie über Ziele und Vorgehen der oben genannten Studie zu informieren. Diese wird vom Institut für Pflegewissenschaft der Universität zu Köln durchgeführt. **Ihre Studienteilnahme ist freiwillig.**

Bitte lesen Sie sich den nachfolgenden Text aufmerksam durch. Wenn Sie noch Fragen haben, sprechen Sie gerne Ihre Stationsleitung an oder kontaktieren Sie uns. Die Kontaktdaten finden Sie am Ende des Schreibens.

I. Information über die Studie

Hintergrund und Ziele

Schlaf ist ein Zustand, der für die Gesundheit, das Wohlbefinden und das tägliche Funktionieren bedeutsam ist. Obwohl Krankheiten mit einem gesteigerten Bedürfnis nach Ruhe und Schlaf einhergehen, legen bisherige wissenschaftliche Untersuchungen nahe, dass Schlafprobleme während eines Krankenhausaufenthaltes häufig auftreten und mit verschiedenen Risiken verbunden sind.

Im Rahmen der Studie „Sleep Acute“ wird das Ziel verfolgt, mehr über den Schlaf und mögliche Schlafprobleme von Patientinnen und Patienten während eines stationären Aufenthalts im Krankenhaus zu erfahren. Darüber hinaus sollen Ansätze zur Schlafförderung erfasst werden.

Parallel untersuchen wir Akzeptanz und Wirksamkeit der von uns in der Schlafstudie eingesetzten schriftlichen Informationsmaterialen. Dies ermöglicht uns, wichtige Erkenntnisse zur Gestaltung von Informationsmaterialien in zukünftigen wissenschaftlichen Studien zu gewinnen.

Potentielle Teilnehmerinnen und Teilnehmer

An der Studie können Sie teilnehmen, wenn Sie volljährig sind und zum Zeitpunkt der Datenerhebung mindestens zwei Nächte im Krankenhaus auf einer sogenannten Normalstation (d. h. keine Überwachungs- oder Intensivstation) verbracht haben.

Abläufe

Bei einer Studienteilnahme werden Ihnen, neben der vorliegenden schriftlichen Studieninformation, zwei Fragebögen übergeben. In einem der Fragebögen werden Sie um Angaben zu Ihrem Schlaf im Krankenhaus und Ihrem Schlaf zu Hause gebeten. Dies ermöglicht uns, Ihren Schlaf während der stationären Versorgung mit Ihrem gewöhnlichen Schlaf zu vergleichen. In dem anderen Fragebogen werden. Sie nach Ihrer Meinung zu dem vorliegenden Informationsschreiben gefragt. Zudem werden Angaben zu Ihrer Person (wie Alter und Geschlecht) erfragt.

Die Beantwortung der Fragen wird insgesamt rund 15 bis 20 Minuten Zeit in Anspruch nehmen. Legen Sie die ausgefüllten Fragebögen anschließend bitte in den beiliegenden Umschlag und verschließen diesen. Die verschlossenen Umschläge werden am Tag, nachdem sie verteilt wurden, von Mitarbeiterinnen bzw. Mitarbeitern Ihrer Station eingesammelt und im Anschluss den Forschenden übergeben.

Zusätzlich werden Daten zu Ihrem Krankenhausaufenthalt (Aufnahmediagnose, Fachbereich, Operationen, Aufnahmetag und -art, Anzahl der Mitpatientinnen bzw. -patienten im Zimmer und Schlafmedikamente) benötigt. Um die Dauer der Befragung für Sie möglichst gering zu halten, werden diese Angaben durch Mitarbeiterinnen bzw. Mitarbeiter Ihrer Station erfasst.

Nutzen und Risiken

Die Teilnahme an der Studie ergibt keinen persönlichen Vorteil für Sie. Diese kann allerdings helfen, mehr über den Schlaf während eines Krankenhausaufenthalts und den Einsatz von Studieninformationsschreiben zu erfahren. Die gewonnenen Erkenntnisse dienen der Aufdeckung von Bedarfen und der Ableitung von Verbesserungspotentialen. Eine persönliche Information über die wissenschaftlichen Ergebnisse der Studie erfolgt nicht.

Eine Belastung durch die Teilnahme an der Studie kann grundsätzlich nicht ausgeschlossen werden. Dieses Risiko ist jedoch als gering einzuschätzen.

II. Datenschutz

Studienspezifische Informationen

(1) Datenverarbeitung und -nutzung

Im Rahmen der Studie werden personenbezogene Daten (d. h. persönliche und gesundheitsbezogene Informationen) im Sinne der Art. 4 Abs. 1 und Art. 9 der Datenschutz-Grundverordnung (DS-GVO) in Papierform über Sie erhoben, verarbeitet sowie genutzt. Die Verwendung Ihrer Daten erfolgt nach gesetzlichen Bestimmungen (gemäß Art. 6 Abs. 1 lit. a,

7 und 9 Abs. 2 lit. a der DS-GVO) und setzt vor der Teilnahme an der Studie Ihre Einwilligung voraus.

Sämtliche Informationen, die wir im Rahmen der Studie über Sie erheben, liegen zunächst in pseudonymisierter Form (d. h. ohne Nennung eines Namens oder identifizierender Daten, sondern mit einem zugeordneten Nummern- und Buchstabencode) vor. Anhand einer sogenannten Codeliste, die sowohl die vollständigen Namen als auch die Pseudonyme enthält, können die Fragebögen Personen zugeordnet werden. Die Verwendung der verschlossenen Umschläge gewährleistet hierbei jedoch, dass identifizierende Daten und Fragebögen zu jeder Zeit getrennt voneinander aufbewahrt werden. Einsicht in die Codeliste hat ausschließlich eine Mitarbeiterin oder ein -mitarbeiter Ihrer Station. Diese oder dieser wird dazu aufgefordert, die Codeliste unmittelbar nach Abschluss der Datenerhebung unwiderruflich zu vernichten. Ab diesem Zeitpunkt liegen die Daten in anonymisierter Form (d. h. die Daten können keiner bestimmten oder bestimmbaren Person zugeordnet werden) vor.

Erst nach dem Eintreffen der Studienmaterialien im Studienzentrum werden die von Ihnen verschlossenen Umschläge durch die Forschenden geöffnet. Sämtliche Informationen, die wir im Rahmen der Studie über Sie erheben, werden im Sinne des Datenschutzes sicher aufbewahrt und gegen unbefugten Zugriff gesichert. Die anonymisierten Daten werden am Institut für Pflegewissenschaft der Universität zu Köln gespeichert und ausgewertet.

Alle Daten werden ausschließlich für Zwecke der wissenschaftlichen Forschung und entsprechend der informierten Einwilligung verwendet. Es ist vorgesehen, dass die gesammelten Daten für Veröffentlichungen genutzt werden. Hierin werden Sie als Teilnehmerin oder Teilnehmer nicht erkennbar sein. Zudem besteht die Möglichkeit, dass die in der Studie über Sie erhobenen Daten in zukünftigen Forschungsarbeiten (sogenannten „Sekundärdatenanalysen“) erneut verwendet sowie anonymisiert an nationale und internationale Kooperationspartnerinnen und -partner innerhalb und außerhalb der Universität zu Köln übermittelt werden.

(2) Dauer der Speicherung

Die gesammelten Daten werden über einen Zeitraum von zehn Jahren durch die Universität zu Köln aufbewahrt und anschließend gelöscht.

(3) Widerspruchs- und Beseitigungsmöglichkeit

Die Teilnahme an der Studie ist freiwillig. Sie können Ihre Teilnahme jederzeit, ohne die Angabe von Gründen, beenden. Hieraus entsteht kein Nachteil für Sie. Auf Ihren Wunsch hin, werden Ihre Daten korrigiert, anonymisiert bzw. gelöscht. Ebenso können Sie der weiteren Verarbeitung Ihrer Daten widersprechen oder diese einschränken. Widerspruch kann Ihrer Stationsleitung und/oder den Forschenden mündlich oder schriftlich mitgeteilt werden.

Wurden die Daten bereits anonymisiert, können die Daten nicht mehr mit Ihnen in Verbindung gebracht werden. Wir weisen daher darauf hin, dass bereits anonymisierte Daten und Daten,

die in wissenschaftliche Auswertungen eingeflossen sind, nicht mehr auf Wunsch gelöscht werden können.

Allgemeine Informationen

(1) Verantwortliche gem. Art. 4 Abs. 7 EU-Datenschutz-Grundverordnung (DS-GVO) sind:

Universität zu Köln
Gesa Diekmann und Alexander May
Albertus-Magnus-Platz
50923 Köln
Telefon: 0221 470 3872
E-Mail: dsb@verw.uni-koeln.de
Webseite: https://verwaltung.uni-koeln.de/stabsstelle02.3/

Unsere Datenschutzbeauftragten erreichen Sie unter zuvor genannten Kontaktdaten oder unserer Postadresse (siehe oben) mit dem Zusatz „Datenschutzbeauftragte".

(2) Sie haben das Recht, sich bei einer Datenschutz-Aufsichtsbehörde über die Verarbeitung Ihrer personenbezogenen Daten in unserem Unternehmen zu beschweren.

Landesbeauftragte für Datenschutz und Informationsfreiheit Nordrhein-Westfalen:

Helga Block
Kavalleriestraße 2-4
40213 Düsseldorf
Telefon: 0211 384240
E-Mail: poststelle@ldi.nrw.de
Webseite: https://www.ldi.nrw.de

(3) Bei Anliegen, Fragen oder Beschwerden zur Datenverarbeitung und zur Einhaltung der datenschutzrechtlichen Anforderungen können Sie sich selbstverständlich auch jederzeit an den Studienleiter wenden. Die Kontaktdaten finden Sie am Ende dieses Schreibens.

(4) Die Studienleitung wird alle angemessenen Schritte unternehmen, um den Schutz Ihrer Daten gemäß Datenschutz-Grundverordnung und anderen Gesetzen zu gewährleisten. Die Daten sind gegen unbefugten Zugriff gesichert. Die personenbezogenen Daten werden anonymisiert, sobald dies nach dem Forschungszweck möglich ist, es sei denn berechtigte Interessen der betroffenen Person stehen dem entgegen. Bis dahin werden die Merkmale gesondert gespeichert, mit denen Einzelangaben über persönliche oder sachliche Verhältnisse einer bestimmten oder bestimmbaren Person zugeordnet werden können. Sie werden mit den Einzelangaben nur zusammengeführt, soweit der Forschungszweck dies erfordert.

(5) Der Verantwortliche wird personenbezogene Daten nur veröffentlichen, wenn Sie in die Veröffentlichung ausdrücklich eingewilligt haben.

(6) Sie haben das Recht, Ihre datenschutzrechtliche Einwilligungserklärung jederzeit zu widerrufen. Durch den Widerruf der Einwilligung wird die Rechtmäßigkeit der aufgrund der Einwilligung bis zum Widerruf erfolgten Verarbeitung nicht berührt.

III. Kontakt zum Studienteam

Institut für Pflegewissenschaft, Medizinische Fakultät, Universität zu Köln
Prof. Dr. phil. Sascha Köpke
Gleueler Straße 176-178
50935 Köln
Telefon: 0221 478 51658
E-Mail: sascha.koepke@uk-koeln.de

Sleep Acute

Wissenschaftliche Studie zur Untersuchung des Schlafs von Patientinnen und Patienten während der stationären Versorgung im Krankenhaus

Verantwortlich gemäß §4 Abs. 7 DS-GVO:

Institut für Pflegewissenschaft, Medizinische Fakultät, Universität zu Köln
Prof. Dr. phil. Sascha Köpke
Gleueler Straße 176-178
50935 Köln
Telefon: 0221 478 51658
E-Mail: sascha.koepke@uk-koeln.de

Einwilligungserklärung für Patientinnen und Patienten

____ (Name der aufklärenden Person) hat am __________________ mit mir ____ (Name der teilnehmenden Person) ein Informationsgespräch über Art, Umfang und Bedeutung der oben genannten Studie geführt. Alle meine Fragen wurden zufriedenstellend beantwortet.

Die Studie „Sleep Acute" zielt darauf, mehr über den Schlaf während der stationären Versorgung im Krankenhaus zu erfahren. Hiervon ausgehend sollen Bedarfe aufgedeckt und Ansätze zur Verbesserung der aktuellen Versorgungssituation abgeleitet werden. Zusätzlich werden Akzeptanz und Wirksamkeit der eingesetzten Studieninformationsmaterialien untersucht. Dies dient der Entwicklung von Empfehlungen für die Gewinnung potentieller Studienteilnehmerinnen und -teilnehmer in zukünftigen Forschungsvorhaben.

Mir ist erläutert worden, dass bei dieser Studie personenbezogene Daten verarbeitet werden sollen. Mir ist insbesondere bekannt, zu welchem Zweck, in welchem Umfang, auf welcher Rechtsgrundlage und wie lange meine Daten gespeichert werden sollen. Darüber hinaus kenne ich meine Rechte gegenüber der verantwortlichen Stelle hinsichtlich meiner personenbezogenen Daten.

Eine entsprechende schriftliche Studieninformation habe ich erhalten, gelesen und verstanden.

Mir ist bekannt, dass die Teilnahme freiwillig ist und ich sie jederzeit, ohne Angabe von Gründen und ohne persönlichen Nachteil, schriftlich oder mündlich widerrufen kann. Meine Daten werden dann vollständig gelöscht. Hierüber erhalte ich eine Nachricht.

Ich hatte ausreichend Zeit, um über eine Teilnahme an der Studie nachzudenken und eine Entscheidung zu treffen.

Ich erkläre mich bereit, an der oben genannten Studie teilzunehmen und willige in die hiermit verbundene und mir bekannte Verarbeitung meiner personenbezogenen Daten ein. Soweit besondere personenbezogene Daten im Sinne des Art. 9 DS-GVO, wie etwa Gesundheitsdaten, erhoben werden, bezieht sich meine Einwilligung auch auf diese Angaben.

Ein Exemplar dieser Einwilligungserklärung habe ich erhalten.

____________________________	____________________________	____________________________
Ort und Datum	Name und Vorname (Druckschrift) der an der Studie teilnehmenden Person	Unterschrift der an der Studie teilnehmenden Person

Die an der Studie teilnehmende Person wurde von mir über die Ziele, die Dauer, den Ablauf, den Nutzen und sämtliche Risiken der Untersuchung mündlich und schriftlich aufgeklärt. Aufgetretene Fragen wurden von mir verständlich und ausreichend beantwortet. Die teilnehmende Person hat ohne Zwang eine Einwilligung erteilt. Die schriftliche Studieninformation und eine Kopie der vorliegenden Einwilligungserklärung habe ich der teilnehmenden Person ausgehändigt.

____________________________	____________________________	____________________________
Ort und Datum	Name und Vorname (Druckschrift) der aufklärenden Person	Unterschrift der aufklärenden Person

Version 1 vom 24.10.2020 Seite 2 von 2

Anhang 8a: Informations- und Einwilligungsflyer für Patient*innen (Teil I und II)

Studienteilnahme

Wer kann an der Studie teilnehmen?

An der Studie können Sie teilnehmen, wenn Sie volljährig sind und zum Zeitpunkt der Datenerhebung mindestens zwei Nächte im Krankenhaus auf einer Normalstation (d. h. keine Überwachungs- oder Intensivstation) verbracht haben.

Wie sieht eine Teilnahme an der Studie aus?

- Bei einer Studienteilnahme werden Sie während Ihres Krankenhausaufenthalts mittels Fragebogen um Angaben zu Ihrem Schlaf vor und während der stationären Versorgung sowie um eine Bewertung des schriftlichen Informationsmaterials zur Studie gebeten. Zudem werden Fragen zu Ihrer Person gestellt.
- Die Befragung dauert rund 15 bis 20 Minuten.
- Die ausgefüllten Fragebögen werden in den beiliegenden Umschlag gelegt und einen Tag, nachdem sie verteilt wurden, eingesammelt.
- Angaben zu Ihrem Krankenhausaufenthalt werden durch eine Mitarbeiterin oder einen Mitarbeiter Ihrer Station erfasst.

Was sind Nutzen und Risiken der Studie?

Die Studienteilnahme ergibt keinen persönlichen Vorteil für Sie. Sie leisten hiermit jedoch einen wichtigen Beitrag zum Erreichen der Forschungsziele.

Eine Belastung durch die Studienteilnahme kann nicht gänzlich ausgeschlossen werden. Dieses Risiko ist allerdings als gering einzuschätzen.

Version 2 vom 23.12.2020

Datenschutz

- Verantwortlich gemäß Art. 4 Abs. 7 DS-GVO sind die Datenschutzbeauftragten der Universität zu Köln[1].
- Bei Bedarf können Sie sich an den Studienleiter[2] wenden.
- Sie haben zudem das Recht, sich bei einer Datenschutz-Aufsichtsbehörde[3] zu beschweren.

Datenverarbeitung, -nutzung und -speicherung

- In der Studie werden personenbezogene (d. h. persönliche und gesundheitsbezogene) Daten in Papierform erhoben, verarbeitet und genutzt.
- Die Verwendung der Daten setzt eine schriftliche Einwilligung von Ihnen voraus.
- Die Daten liegen zuerst in pseudonymisierter Form (d. h. ohne Nennung identifizierender Daten, sondern mit Nummern- und Buchstabencode) vor. Anhand einer Liste, die Namen und Pseudonyme enthält, können die Daten Personen zugeordnet werden. Die Liste wird nach Abschluss der Datenerhebung unwiderruflich vernichtet, sodass die Daten anonymisiert (d. h. keiner bestimmten oder bestimmbaren Person zuordenbar) werden.
- Die anonymisierten Daten werden am Institut für Pflegewissenschaft der Universität zu Köln im Sinne des Datenschutzes sicher aufbewahrt und gegen unbefugten Zugriff gesichert. Die Löschung erfolgt nach zehn Jahren.
- In dieser Zeit werden die Daten ausschließlich für Zwecke der wissenschaftlichen Forschung und gemäß der informierten Einwilligung verwendet. Es ist vorgesehen, die Daten für Veröffentlichungen und ggf. erneut in Untersuchungen zu nutzen. Zudem besteht die Möglichkeit des Datentransfers an Kooperationspartnerinnen und -partner.

Widerspruchs- und Beseitigungsmöglichkeit

- Die Teilnahme an der Studie ist freiwillig und kann jederzeit, ohne die Angabe von Gründen und ohne persönlichen Nachteil, beendet werden. Widerspruch kann Ihrer Stationsleitung und/oder den Forschenden mündlich oder schriftlich mitgeteilt werden.
- Anonymisierte Daten können einer Person nicht länger zugeordnet und daher auf Wunsch nicht gelöscht werden.

Information zu einer wissenschaftlichen Studie des Instituts für Pflegewissenschaft der Universität zu Köln

Sleep Acute

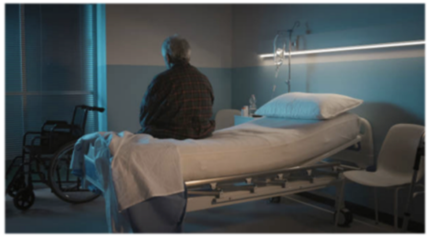

Befragung von Patientinnen und Patienten zum Schlaf während der stationären Versorgung im Krankenhaus

Liebe Patientin, lieber Patient,

die Studie **Sleep Acute** zielt darauf, mehr über den Schlaf während eines stationären Aufenthalts im Krankenhaus zu erfahren. Sie selbst sind aktuell Patientin oder Patient im Krankenhaus und können uns daher mit einer **freiwilligen Studienteilnahme** bei unserem Forschungsvorhaben unterstützen.

Informationsvideo

Durch Aufrufen des folgenden Links oder QR-Codes gelangen Sie zu einem Video, in dem wir Sie über Ziele und Vorgehen der Studie informieren.

QR-Code

Link

Unabhängig von dem Video, finden Sie die für eine Studienteilnahme benötigten Informationen ebenfalls in dem vorliegenden Flyer.

Sollten Fragen unbeantwortet bleiben, sprechen Sie gerne Ihre Stationsleitung an oder kontaktieren Sie uns. Die Kontaktdaten finden Sie am Ende des Flyers.

Ihr Studienteam

Version 2 vom 23.12.2020

Hintergrund und Studienziele

Schlaf ist ein dynamischer Zustand, der für die Gesundheit, das Wohlbefinden und das tägliche Funktionieren von großer Bedeutung ist. Obwohl Krankheiten mit einem gesteigerten Bedürfnis nach Ruhe und Schlaf einhergehen, legen bisherige wissenschaftliche Untersuchungen nahe, dass Schlafprobleme während eines Aufenthalts im Krankenhaus häufig auftreten.

Wir verfolgen das Ziel, mehr über den Schlaf und mögliche Schlafprobleme von Patientinnen und Patienten während eines stationären Aufenthalts im Krankenhaus zu erfahren. Hiervon ausgehend sollen Bedarfe aufgedeckt und Ansätze zur Verbesserung der Versorgungssituation abgeleitet werden.

Parallel untersuchen wir Akzeptanz und Wirksamkeit der von uns in der Schlafstudie eingesetzten schriftlichen Informationsmaterialien. Dies dient dem Zweck, Erkenntnisse zur Gestaltung von Informationsmaterialien für zukünftige Studien zu gewinnen.

Kontakt

[2]Studienleitung

Prof. Dr. phil. Sascha Köpke
Institut für Pflegewissenschaft der Universität zu Köln
Gleueler Straße 176-178
50935 Köln
0221 478 51658
sascha.koepke@uk-koeln.de
https://pflegewissenschaft.uni-koeln.de

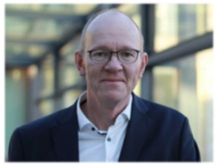

[1]Datenschutzbeauftragte der Universität zu Köln

Gesa Diekmann und Alexander May
Albertus-Magnus-Platz
50923 Köln
0221 470 3872
dsb@verw.uni-koeln.de
https://verwaltung.uni-koeln.de/stabsstelle02.3/

[3]Landesbeauftragte für Datenschutz und Informationsfreiheit NRW

Helga Block
Kavalleriestraße 2-4
40213 Düsseldorf
0211 384240
poststelle@ldi.nrw.de
https://www.ldi.nrw.de

Einwilligung zu einer wissenschaftlichen Studie des Instituts für Pflegewissenschaft der Universität zu Köln

Sleep Acute

Befragung von Patientinnen und Patienten zum Schlaf während der stationären Versorgung im Krankenhaus

Name der teilnehmenden Person:

____

Name der aufklärenden Person:

____

Version 1 vom 24.10.2020

(Vorderseite)

Mit mir wurde ein Informationsgespräch über Art, Umfang und Bedeutung der Studie geführt. Alle meine Fragen wurden zufriedenstellend beantwortet.

Die Studie **Sleep Acute** zielt darauf, mehr über den Schlaf während der stationären Versorgung im Krankenhaus zu erfahren. Hiervon ausgehend sollen Bedarfe aufgedeckt und Ansätze zur Verbesserung der Versorgungssituation abgeleitet werden. Zusätzlich werden Akzeptanz und Wirksamkeit der eingesetzten Studieninformationsmaterialien untersucht. Dies dient der Entwicklung von Empfehlungen für die Gewinnung potentieller Studienteilnehmerinnen und -teilnehmer in zukünftigen Forschungsvorhaben.

Mir ist erläutert worden, dass bei der Studie personenbezogene Daten verarbeitet werden. Mir ist bekannt, zu welchem Zweck, in welchem Umfang, auf welcher Rechtsgrundlage und wie lange meine Daten gespeichert werden. Darüber hinaus kenne ich meine Rechte gegenüber der verantwortlichen Stelle.

Eine entsprechende schriftliche Studieninformation habe ich erhalten, gelesen und verstanden.

Mir ist bekannt, dass die Studienteilnahme freiwillig ist und ich sie jederzeit, ohne Angabe von Gründen und ohne persönlichen Nachteil, widerrufen kann.

Ich erkläre mich bereit, an der Studie teilzunehmen und willige in die hiermit verbundene Verarbeitung meiner personenbezogenen Daten ein. Meine erteilte Einwilligung bezieht sich auch auf besondere personenbezogene Daten im Sinne des Art. 9 DS-GVO.

____

Ort und Datum, Unterschrift der teilnehmenden Person

Version 1 vom 24.10.2020

(Rückseite)

Anhang 8b: Skript für das Informationsvideo für Patient*innen (Teil I und II)

Informationsvideo für Patient*innen – Skript

Rahmenbedingungen:

- Gesamtdauer: Ca. 2-5 Minuten
- Sprecher*innen: Sascha Köpke und Marcelina Roos
- Aufnahmeort: Institut für Pflegewissenschaft der Universität zu Köln

Inhalte:

Was steckt hinter der Studie „Sleep Acute"?

„Sleep Acute" ist eine pflegewissenschaftliche Studie, die vom Institut für Pflegewissenschaft der Universität zu Köln durchgeführt wird. Die Studie zielt primär darauf, mehr über den Schlaf von Patientinnen und Patienten während eines stationären Aufenthalts im Krankenhaus zu erfahren. Wir erhoffen uns, aus den gewonnenen Erkenntnissen Bedarfe aufdecken und Ansätze zur Verbesserung der Schlafsituation ableiten zu können.

Parallel gehen wir einer weiteren Forschungsfrage nach, indem wir die Akzeptanz und Wirksamkeit der von uns in der Schlafstudie eingesetzten schriftlichen Informationsmaterialien untersuchen. Ziel ist es, Informationen darüber zu erhalten, wie Studienteilnehmerinnen und -teilnehmer auch zukünftig erfolgreich für Forschungsvorhaben gewonnen werden können."

Wer kann an der Studie teilnehmen und wie sieht die Studienteilnahme konkret aus?

„An der Studie können Patientinnen und Patienten teilnehmen, die volljährig sind und zum Zeitpunkt der Datenerhebung bereits mindestens zwei Nächte im Krankenhaus auf einer sogenannten Normalstation, das heißt keiner Überwachungs- oder Intensivstation, verbracht haben.

Die Studienteilnahme beinhaltet das Ausfüllen zweier Fragebögen. In einem der Fragebögen werden Fragen zum Schlaf der Patientinnen und Patienten vor und während des Krankenhausaufenthalts gestellt. Dies ermöglicht uns, den Schlaf während der stationären Versorgung mit dem gewöhnlichen Schlaf zu vergleichen. In dem anderen Fragebogen werden Patientinnen und Patienten um eine Bewertung der ausgehändigten Informationsmaterialien zur Schlafstudie gebeten. Einzelne Fragen beziehen sich außerdem auf persönliche Angaben, wie etwa das Alter oder das Geschlecht, der teilnehmenden Personen. Das Ausfüllen der Fragebögen dauert voraussichtlich 15 bis 20 Minuten.

Um die Dauer der Befragung für teilnehmende Patientinnen und Patienten möglichst gering zu halten, werden weitere Daten, die sich auf den Krankenhausaufenthalt beziehen, über Mitarbeiterinnen oder Mitarbeitern der Station gesammelt. Hierzu gehören zum Beispiel Informationen zum Aufnahmetag, der Aufnahmediagnose oder der Einnahme von Schlafmedikamenten.

Warum sollten Patientinnen und Patienten an der Studie teilnehmen?

„Eine erfolgreiche Umsetzung unserer Studie lässt sich nur mit der Unterstützung der Patientinnen und Patienten realisieren. Mit einer Teilnahme wird also ein wichtiger Beitrag geleistet, erstmals für Deutschland, die Schlafsituation von Patientinnen und Patienten im Krankenhaus angemessen zu beschreiben, Bedarfe aufzudecken und Ansätze zur Schlafförderung abzuleiten. Die parallele Untersuchung der Informationsmaterialien ermöglicht uns außerdem wichtige Erkenntnisse zur Gestaltung der Informationsmaterialien in zukünftigen wissenschaftlichen Studien zu gewinnen."

Abschluss

„Selbstverständlich ist Ihre Teilnahme an der Studie freiwillig. Weitere Informationen zur Studie finden Sie in dem Ihnen ausgehändigten Flyer. Sollten Sie Fragen haben, sprechen Sie gerne Ihre Stationsleitung an oder nehmen Sie Kontakt zu uns auf. Unsere Kontaktdaten finden Sie auf der Rückseite des Flyers."

Anhang 9: Fragebogen zur Bewertung der schriftlichen Studieninformation

Studien-ID: _____ _____ – _____ _____ – _____ _____ **Datum:** _____._____.__________

Fragebogen zur Bewertung der schriftlichen Studieninformation

Bitte beantworten Sie die folgenden Fragen zu der Ihnen ausgehändigten schriftlichen Studieninformation, indem Sie jeweils <u>einen</u> für Sie passenden Wert auf den Antwortskalen auswählen.

1. Wie bewerten Sie es, dass Ihnen eine schriftliche Studieninformation ausgehändigt wurde?

0 1 2 3 4 5

Sehr negativ … Sehr positiv

2. Hat die schriftliche Studieninformation Sie angeregt, an der Studie teilzunehmen?

0 1 2 3 4 5

Überhaupt nicht … Sehr

3. Wie bewerten Sie die schriftliche Studieninformation insgesamt?

0 1 2 3 4 5

Sehr negativ … Sehr positiv

4. Wie bewerten Sie Format und Gestaltung der schriftlichen Studieninformation?

0 1 2 3 4 5

Sehr negativ … Sehr positiv

Seite 1 von 3

5. Wie bewerten Sie die schriftliche Studieninformation in Bezug auf ihre Verständlichkeit?

0 — 1 — 2 — 3 — 4 — 5

Überhaupt nicht verständlich … Sehr verständlich

6. Wie bewerten Sie die schriftliche Studieninformation in Bezug auf ihre Vollständigkeit?

0 — 1 — 2 — 3 — 4 — 5

Nicht vollständig … Vollständig

7. Wie bewerten Sie die schriftliche Studieninformation in Bezug auf ihren Umfang?

0 — 1 — 2 — 3 — 4 — 5

Überhaupt nicht angemessen … Vollkommen angemessen

8. Wie bewerten Sie die Inhalte der schriftlichen Studieninformation in Bezug auf ihre Relevanz?

0 — 1 — 2 — 3 — 4 — 5

Überhaupt nicht relevant … Sehr relevant

Bitte notieren Sie Ihre Antworten im Folgenden jeweils unterhalb der Fragen.

9. Was gefällt Ihnen an der schriftlichen Studieninformation besonders gut?

____

____

____

____

____

10. Was gefällt Ihnen an der schriftlichen Studieninformation weniger gut?

____

____

____

____

____

11. Was sollte an der schriftlichen Studieninformation verändert werden?

____

____

____

____

____

Anhang 10: Informations- und Einwilligungsschreiben für Pflegefachpersonen (Teil I)

Sleep Acute

Wissenschaftliche Studie zur Untersuchung des Schlafs von Patientinnen und Patienten während der stationären Versorgung im Krankenhaus

Verantwortlich gemäß §4 Abs. 7 DS-GVO:

Institut für Pflegewissenschaft, Medizinische Fakultät, Universität zu Köln
Prof. Dr. phil. Sascha Köpke
Gleueler Straße 176-178
50935 Köln
Telefon: 0221 478 51658
E-Mail: sascha.koepke@uk-koeln.de

Informationsschreiben für Pflegefachpersonen

Sehr geehrte Damen und Herren,

das vorliegende Schreiben dient dazu, Sie über Ziele und Vorgehen der oben genannten wissenschaftlichen Studie zu informieren. Diese wird vom Institut für Pflegewissenschaft der Universität zu Köln durchgeführt. Ihre Studienteilnahme ist freiwillig.

Bitte lesen Sie sich den nachfolgenden Text aufmerksam durch. Wenn Sie noch Fragen haben, kontaktieren Sie uns gerne. Die Kontaktdaten finden Sie am Ende des Schreibens.

I. Information über die Studie

Hintergrund und Ziele

Schlaf ist ein dynamischer Zustand, der für die Gesundheit, das körperliche und geistige Wohlbefinden sowie das tägliche Funktionieren von großer Bedeutung ist. Obwohl Krankheiten mit einem gesteigerten Bedürfnis nach Ruhe und Schlaf einhergehen, legen bisherige wissenschaftliche Untersuchungen nahe, dass Schlafprobleme während eines Krankenhausaufenthaltes regelhaft auftreten und als Stress erlebt werden.

Im Rahmen der Studie „Sleep Acute“ werden die Ziele verfolgt, Schlafprobleme und Schlafqualität von Patientinnen und Patienten im Krankenhaus zu beschreiben sowie Faktoren zu identifizieren, die mit Schlafproblemen in Beziehung stehen. Darüber hinaus sollen Strategien und Maßnahmen zur Schlafförderung im Krankenhaus aufgedeckt werden.

Potentielle Teilnehmerinnen und Teilnehmer

An der Studie können Sie teilnehmen, wenn Sie Stationsleitung oder stellvertretende Stationsleitung einer der an der Studie teilnehmenden Stationen sind. Darüber hinaus muss

Ihr Stellenumfang mindestens 50 Prozent der regelmäßigen wöchentlichen Arbeitszeit betragen.

Abläufe

Bei einer Studienteilnahme wird Ihnen ein Fragebogen übergeben. Hierin werden Ihnen Fragen zu aktuell angewendeten Strategien und Maßnahmen zur Schlafförderung bei Patientinnen und Patienten im Krankenhaus gestellt. Zudem werden Informationen zu Ihrer Person (Alter, Geschlecht, Fachbereich sowie Berufsqualifikation und -erfahrung) erfragt.

Die Beantwortung der Fragen wird rund 15 bis 20 Minuten Zeit in Anspruch nehmen. Legen Sie den ausgefüllten Fragebogen anschließend bitte in den beiliegenden Umschlag und verschließen diesen. Die verschlossenen Umschläge werden noch am selben Tag, an dem sie verteilt wurden, an die Forschenden übergeben.

Nutzen und Risiken

Die Teilnahme an der Studie ergibt keinen persönlichen Vorteil für Sie. Diese kann allerdings helfen, mehr über den Schlaf während der stationären Versorgung im Krankenhaus zu erfahren. Hiervon ausgehend sollen Bedarfe aufgedeckt und Ansätze zur Verbesserung der Versorgungssituation abgeleitet werden.

Eine Belastung durch die Teilnahme an der Studie kann grundsätzlich nicht ausgeschlossen werden. Dieses Risiko ist jedoch als gering einzuschätzen.

II. Datenschutz

Allgemeine Informationen

(1) Verantwortliche gem. Art. 4 Abs. 7 EU-Datenschutz-Grundverordnung (DS-GVO) sind:

Universität zu Köln
Gesa Diekmann und Alexander May
Albertus-Magnus-Platz
50923 Köln
Telefon: 0221 470 3872
E-Mail: dsb@verw.uni-koeln.de
Webseite: https://verwaltung.uni-koeln.de/stabsstelle02.3/

Unsere Datenschutzbeauftragten erreichen Sie unter zuvor genannten Kontaktdaten oder unserer Postadresse (siehe oben) mit dem Zusatz „Datenschutzbeauftragte".

(2) Sie haben das Recht, sich bei einer Datenschutz-Aufsichtsbehörde über die Verarbeitung Ihrer personenbezogenen Daten in unserem Unternehmen zu beschweren.

Landesbeauftragte für Datenschutz und Informationsfreiheit Nordrhein-Westfalen:

Helga Block
Kavalleriestraße 2-4
40213 Düsseldorf

Telefon: 0211 384240
E-Mail: poststelle@ldi.nrw.de
Webseite: https://www.ldi.nrw.de

(3) Bei Anliegen, Fragen oder Beschwerden zur Datenverarbeitung und zur Einhaltung der datenschutzrechtlichen Anforderungen können Sie sich selbstverständlich auch jederzeit an den Studienleiter wenden. Die Kontaktdaten finden Sie am Ende dieses Schreibens.

(4) Die Studienleitung wird alle angemessenen Schritte unternehmen, um den Schutz Ihrer Daten gemäß Datenschutz-Grundverordnung (DS-GVO) und anderen Gesetzen zu gewährleisten. Die Daten sind gegen unbefugten Zugriff gesichert. Die personenbezogenen Daten werden anonymisiert, sobald dies nach dem Forschungszweck möglich ist, es sei denn berechtigte Interessen der betroffenen Person stehen dem entgegen. Bis dahin werden die Merkmale gesondert gespeichert, mit denen Einzelangaben über persönliche oder sachliche Verhältnisse einer bestimmten oder bestimmbaren Person zugeordnet werden können. Sie werden mit den Einzelangaben nur zusammengeführt, soweit der Forschungszweck dies erfordert.

(5) Der Verantwortliche wird personenbezogene Daten nur veröffentlichen, wenn Sie in die Veröffentlichung ausdrücklich eingewilligt haben.

(6) Sie haben das Recht, Ihre datenschutzrechtliche Einwilligungserklärung jederzeit zu widerrufen. Durch den Widerruf der Einwilligung wird die Rechtmäßigkeit der aufgrund der Einwilligung bis zum Widerruf erfolgten Verarbeitung nicht berührt.

Studienspezifische Informationen

(1) Datenverarbeitung und -nutzung

Im Rahmen der Studie werden personenbezogene Daten (d. h. persönliche Informationen) im Sinne der Art. 4 Abs. 1 in Papierform über Sie erhoben, verarbeitet sowie genutzt. Die Verwendung Ihrer Daten erfolgt nach gesetzlichen Bestimmungen (gemäß Art. 6 Abs. 1 lit. a, 7 und 9 Abs. 2 lit. a der DS-GVO) und setzt vor der Teilnahme an der Studie Ihre freiwillig abgegebene Einwilligungserklärung voraus.

Sämtliche Informationen, die wir im Rahmen der Studie über Sie erheben, werden im Sinne des Datenschutzes sicher aufbewahrt und gegen unbefugten Zugriff gesichert. Die Daten werden in pseudonymisierter Form (d. h. ohne Nennung Ihres Namens oder Ihrer identifizierenden Daten, sondern nur mit einem zugeordneten Nummern- und Buchstabencode) am Institut für Pflegewissenschaft der Medizinischen Fakultät der Universität zu Köln gespeichert und ausgewertet. Ihre identifizierenden Daten (d. h. die Standortdaten) werden getrennt von den übrigen Studiendaten gelagert. Anhand einer sogenannten Codeliste, die vollständige Angaben zu Ihrer Einrichtung und Station sowie Pseudonyme enthält, können die pseudonymisierten Studienmaterialien durch Ihre Position als Stationsleitung indirekt Ihrer Person zugeordnet werden. Nach Abschluss der Datenanalyse und Rückmeldung der Ergebnisse wird diese Liste unwiderruflich vernichtet, sodass die Daten

vollständig anonymisiert (d. h. die Daten können keiner bestimmten oder bestimmbaren Person zugeordnet werden) vorliegen.

Alle Daten werden ausschließlich für Zwecke der wissenschaftlichen Forschung und entsprechend der informierten Einwilligung verwendet. Es ist vorgesehen, dass die gesammelten Daten für Veröffentlichungen genutzt werden. Hierin werden Sie als Teilnehmerin oder Teilnehmer nicht erkennbar sein. Zudem besteht die Möglichkeit, dass die in der Studie über Sie erhobenen Daten in zukünftigen Forschungsarbeiten (sogenannten „Sekundärdatenanalysen“) erneut verwendet sowie anonymisiert an nationale und internationale Kooperationspartnerinnen und -partner innerhalb und außerhalb der Universität zu Köln übermittelt werden.

(2) Dauer der Speicherung

Die gesammelten Daten werden über einen Zeitraum von zehn Jahren durch die Universität zu Köln aufbewahrt und anschließend gelöscht.

(3) Widerspruchs- und Beseitigungsmöglichkeit

Die Teilnahme an der Studie ist freiwillig. Sie können Ihre Teilnahme jederzeit, ohne die Angabe von Gründen, beenden. Hieraus entsteht kein Nachteil für Sie. Auf Ihren Wunsch hin, korrigieren, anonymisieren bzw. löschen wir Ihre Daten. Ebenso können Sie der weiteren Verarbeitung Ihrer Daten widersprechen oder diese einschränken.

Wurden die Daten bereits anonymisiert, können die Daten nicht mehr mit Ihnen in Verbindung gebracht werden. Wir weisen daher darauf hin, dass bereits anonymisierte Daten und Daten, die in wissenschaftliche Auswertungen eingeflossen sind, nicht mehr auf Wunsch gelöscht werden können.

III. Kontakt zum Studienteam

Institut für Pflegewissenschaft, Medizinische Fakultät, Universität zu Köln
Prof. Dr. phil. Sascha Köpke
Gleueler Straße 176-178
50935 Köln
Telefon: 0221 478 51658
E-Mail: sascha.koepke@uk-koeln.de

Sleep Acute

Wissenschaftliche Studie zur Untersuchung des Schlafs von Patientinnen und Patienten während der stationären Versorgung im Krankenhaus

Verantwortlich gemäß §4 Abs. 7 DS-GVO:

Institut für Pflegewissenschaft, Medizinische Fakultät, Universität zu Köln
Prof. Dr. phil. Sascha Köpke
Gleueler Straße 176-178
50935 Köln
Telefon: 0221 478 51658
E-Mail: sascha.koepke@uk-koeln.de

Einwilligungserklärung für Pflegefachpersonen

____ (Name der aufklärenden Person) hat am __________________ mit mir ____ (Name der teilnehmenden Person) ein Informationsgespräch über Art, Umfang und Bedeutung der oben genannten Studie geführt. Alle meine Fragen wurden zufriedenstellend beantwortet.

Die Studie „Sleep Acute" zielt darauf, mehr über den Schlaf während der stationären Versorgung im Akutkrankenhaus zu erfahren. Hiervon ausgehend sollen Bedarfe aufgedeckt und Ansätze zur Verbesserung der aktuellen Versorgungssituation abgeleitet werden.

Mir ist erläutert worden, dass bei dieser Studie personenbezogene Daten verarbeitet werden sollen. Mir ist insbesondere bekannt, zu welchem Zweck, in welchem Umfang, auf welcher Rechtsgrundlage und wie lange meine Daten gespeichert werden sollen. Darüber hinaus kenne ich meine Rechte gegenüber der verantwortlichen Stelle hinsichtlich meiner personenbezogenen Daten.

Ein entsprechendes Informationsschreiben habe ich erhalten, gelesen und verstanden.

Mir ist bekannt, dass die Teilnahme freiwillig ist und ich sie jederzeit, ohne Angabe von Gründen und ohne persönlichen Nachteil, schriftlich oder mündlich widerrufen kann. Meine Daten werden dann vollständig gelöscht. Hierüber erhalte ich eine Nachricht.

Ich hatte ausreichend Zeit, um über eine Teilnahme an der Studie nachzudenken und eine Entscheidung zu treffen.

Ich erkläre mich bereit, an der oben genannten Studie teilzunehmen und willige in die hiermit verbundene und mir bekannte Verarbeitung meiner personenbezogenen Daten ein.

Ein Exemplar dieser Einwilligungserklärung habe ich erhalten.

____________________________	____________________________	____________________________
Ort und Datum	Name und Vorname (Druckschrift) der an der Studie teilnehmenden Person	Unterschrift der an der Studie teilnehmenden Person

Die an der Studie teilnehmende Person wurde von mir über die Ziele, die Dauer, den Ablauf, den Nutzen und sämtliche Risiken der Untersuchung mündlich und schriftlich aufgeklärt. Aufgetretene Fragen wurden von mir verständlich und ausreichend beantwortet. Die teilnehmende Person hat ohne Zwang eine Einwilligung erteilt. Das Informationsschreiben und eine Kopie der vorliegenden Einwilligungserklärung habe ich der teilnehmenden Person ausgehändigt.

____________________________	____________________________	____________________________
Ort und Datum	Name und Vorname (Druckschrift) der aufklärenden Person	Unterschrift der aufklärenden Person

Version 1 vom 24.10.2020 Seite 2 von 2

Anhang 11: Informationsschreiben (Teil III) und Fragebogen zur Entscheidungsfindung hinsichtlich der Verifikation der korrekten Magensondenlage

(Dieser Anhang enthält zusätzliche Details zur MaSoLa-Study)

Study Protocol

Sleep Acute

Part I: Sleep of patients during inpatient hospital care

Part II: Acceptance and Effectiveness of different information letters for recruiting hospital patients

Marcelina Roos, B.Sc.

Research Fellow

Martin N. Dichter, Ph.D.

PostDoc, Research Fellow

Prof. Dr. phil. Sascha Köpke

Head of Institute

Institute of Nursing Science

University of Cologne, Faculty of Medicine and University Hospital Cologne

Gleueler Str. 176-178

50935 Cologne, Germany

Abstract – Part I

Background

Sleep is a complex and dynamic state that is crucial for health, physical and mental well-being, and daily functioning. Although illness and injury are associated with an increased need for rest and sleep, previous scientific studies suggest that sleep disturbances frequently occur during hospital stays and are perceived by patients as a stressor. The reasons for this are diverse. At the same time, there is a lack of large-scale, multicentre studies, and thus of meaningful research findings, to map and assess the current sleep situation of hospital patients.

Objectives

The *Sleep Acute* study aims to pursue the following research objectives:

1. To describe the prevalence of sleep disturbances among hospital patients
2. To describe the subjective sleep quality of hospital patients
3. To identify hospital- and patient-related factors associated with sleep disturbances among hospital patients
4. To identify currently applied strategies and measures for promoting sleep among hospital patients

Methodology

The research objectives are pursued through a multicentre observational study. Between November and December 2020, a cross-sectional survey will be conducted in randomly selected hospitals within a 50 km radius of the study centre, involving approximately 1,000 patients and around 100 nursing professionals. Based on research interests and prior knowledge, data collection and analysis will be carried out using qualitative (research objective 4) and quantitative (research objectives 1 to 4) methods.

Expected Results

The study is expected to provide a valid representation of the current situation regarding sleep in hospitals. The findings will serve both to examine existing assumptions and to gain new insights into current care practices. This, in turn, offers the opportunity to identify needs and derive future research questions.

Abstract – Part II

Background

A key component of the empirical research process is the recruitment of participants. Researchers have access to a wide range of recruitment strategies, techniques, and procedures. While studies suggest that commonly used study information letters may act as a potential barrier to participant recruitment, there is currently a lack of meaningful research findings on the acceptance and effectiveness of both conventional and alternatively designed written study information materials.

Objective

The study aims to examine the acceptance and effectiveness of an engagingly designed study information flyer compared to a formal study information letter for recruiting participants in a questionnaire-based study on sleep in hospitals.

Methodology

As part of the cross-sectional observational study on the sleep of hospitalised patients described in Part I of this study protocol, an embedded cluster-randomised study (SWAT) will be conducted to compare two written study information materials. Approximately 1,000 hospital patients will be cluster-randomised into two study groups, which will differ in the type of written study information provided. To assess the effectiveness of both approaches, response rates and the sociodemographic diversity of the sample will be analysed using quantitative methods. Acceptance will be evaluated using a questionnaire, with quantitative components analysed descriptively and qualitative components examined using content analysis.

Expected Results

The study is expected to determine the acceptance and effectiveness of two different written study information formats. The findings will contribute to the development of concrete recommendations for designing a key component of the empirical research process, thereby facilitating the successful generation and dissemination of scientific research findings.

Table of Contents

PART I: SLEEP OF PATIENTS DURING INPATIENT ACUTE HOSPITAL CARE **1**

Background **1**

Objectives **2**

Methodology **2**

Study Design 2

Sample and Recruitment 2

Data Collection 4

Data Management 7

Data Analysis 8

PART II: ACCEPTANCE AND EFFECTIVENESS OF DIFFERENT INFORMATION LETTERS FOR PATIENT RECRUITMENT IN HOSPITALS **15**

Background **15**

Objectives **16**

Methodology **16**

Study Design 16

Sample 16

Recruitment 17

Data Collection 17

Data Analysis 18

ETHICAL CONSIDERATIONS **20**

Enlightenment and Consent **20**

Potential Risks **20**

Data Protection **22**

REFERENCES **25**

Appendix .... **VIII**

Appendix 1: Consensus Sleep Diary (CSD) – Core Version .... **VIII**

Appendix 2: Consensus Sleep Diary (CSD) – Additional Items .... **IX**

Appendix 3: Sleep Disturbance Item Bank – Short Form 8a .... **X**

Appendix 4: Richards-Campbell Sleep Questionnaire (RCSQ) .... **XI**

Appendix 5: Questions on Hospital-Specific Factors .... **XII**

Appendix 7: Information and Consent Letter for Patients (Part I and II) .... **XIV**

Appendix 8a: Information and Consent Flyer for Patients (Part I and II) .... **XXIX**

Appendix 8b: Script for the Information Video for Patients (Part I and II) .... **XXXII**

Appendix 9: Questionnaire for Evaluating the Written Study Information .... **XXXIV**

Appendix 10: Information and Consent Letter for Healthcare Professionals (Part I) .... **XXXVIII**

PART I: SLEEP OF PATIENTS DURING INPATIENT ACUTE HOSPITAL CARE

Background

Sleep is a complex and dynamic state that is essential for health, physical and mental well-being, and daily functioning (Redeker, Hedges & Booker, 2011). Although illness and injury are associated with an increased need for rest and sleep (Friese, 2008), previous scientific studies suggest that sleep during hospital stays presents a similarly complex challenge.

Existing studies in selected patient populations provide initial indications that sleep disturbances are a common occurrence in hospitals and are perceived by patients as a stressor (Gellerstedt, Medin & Rydell Karlsson, 2014; Lane & East, 2008; Wesselius et al., 2018). Impairments in sleep are associated with various risks, including negative effects on immune and metabolic processes (Ganz, 2012; Hoevenaar-Blom, Spijkerman, Kromhout & Verschuren, 2014; Lange, Dimitrov & Born, 2010), increased pain perception (Raymond, Nielsen, Lavigne, Manzini & Choiniere, 2001), as well as a higher incidence of falls (Stone, Ensrud & Ancoli-Israel, 2008) and delirium (Weinhouse et al., 2009). Additionally, sleep disturbances can negatively impact cognitive and emotional functions, such as processing new information, decision-making, and coping with challenging situations (John et al., 2007; Pilkington, 2013; Rasch & Born, 2013). Consequently, sleep disturbances pose significant risks to patients' health, recovery, and well-being during hospital stays (Raymond et al., 2001).

Given the severe risks associated with sleep disturbances, the question arises as to which measures and strategies can be implemented to promote sleep in hospitals. A fundamental step in this process is the identification of relevant and, most importantly, potentially modifiable factors associated with sleep disturbances (Wesselius et al., 2018). Qualitative research approaches have already identified numerous such factors (Pilkington et al., 2013). However, large-scale, multicentre studies are still lacking. Building on existing knowledge, such studies could further investigate the links between hospital conditions and sleep disturbances while identifying targeted strategies for sleep promotion. How sleep disturbances are currently addressed in everyday hospital care and what measures and strategies are used for sleep promotion remain largely unknown. However, the scientific literature consistently highlights that nursing professionals, due to their close contact with patients, are in a unique position to initiate and implement sleep-promoting interventions (Pellatt, 2007; Radtke, Obermann & Teymer, 2014; Redeker et al., 2011).

Objectives

Sleep in hospitals is an important yet insufficiently researched field. In light of this, a multicentre study is planned within a representative selection of hospitals within a 50 km radius of the study centre. The study aims to address the following research objectives:

1. To describe the prevalence of sleep disturbances among hospital patients
2. To describe the subjective sleep quality of hospital patients
3. To identify hospital-related factors associated with sleep disturbances in hospital patients
4. To identify currently applied strategies and measures for promoting sleep among hospital patients

The study aims to provide a valid representation of the current sleep situation in German hospitals. The generated scientific findings will serve both to examine existing assumptions and to explore current clinical practice. This will enable the identification of needs and the derivation of future research questions. Ultimately, the study aims to contribute to the improvement of patient care practices.

Methodology

Study Design

The study follows a multicentre observational design, methodologically based on a research project conducted in the Netherlands (Wesselius et al., 2018). Between November and December 2020, a cross-sectional survey of patients and nursing professionals will be conducted in randomly selected hospitals near the study centre. Based on research interests and prior knowledge, data collection and analysis will employ both qualitative (research objective 4) and quantitative (research objectives 1 to 4) methods.

Sample and Recruitment

As the study is exploratory in nature, there are currently no valid reference points for sample size calculation or value distribution within the target population. Nevertheless, the sample will be designed to be sufficiently large to yield meaningful and reliable results. The study aims to recruit at least 1,000 patients from approximately 30 hospitals, with around 100 hospital wards included. In addition to patient participation, one nursing professional from each participating ward will also be recruited for the study.

The specific inclusion and exclusion criteria for hospitals, wards, patients, and nursing professionals can be found in Table 1.

Tabelle 1: Ein- und Ausschlusskriterien

Level	Inclusion Criteria	Exclusion Criteria
**Hospitals**	▪ General hospitals (fully inpatient specialist departments)<br>▪ Hospitals offering at least basic care (internal medicine and surgery)<br>▪ Hospitals within a 50 km radius of the study centre	▪ Other hospitals (exclusively psychiatric or neurological beds, or exclusively partial inpatient care)<br>▪ Military hospitals<br>▪ Affiliated hospitals<br>▪ Preventive and rehabilitation facilities
**Wards**	▪ Inpatient wards with beds<br>▪ General wards	▪ Palliative care units<br>▪ Psychiatric wards<br>▪ Paediatric wards<br>▪ Emergency departments<br>▪ Functional departments<br>▪ Monitoring and intensive care units
**Patients**	▪ Age ≥ 18 years<br>▪ Capacity to consent<br>▪ Hospital stay of ≥ two nights on a general ward at the time of data collection<br>▪ Provision of informed consent for study participation	▪ Cognitive and/or physical impairments preventing completion of the questionnaire (clinical decision by ward management)<br>▪ Insufficient German language proficiency preventing completion of the questionnaire (clinical decision by ward management)
**Nursing Professionals**	▪ Ward manager or deputy ward manager of a participating ward<br>▪ Employment of ≥ 50% of regular weekly working hours<br>▪ Provision of informed consent for study participation	▪ /

Based on available hospital directories, 94 hospitals were identified in July 2020 that met the inclusion and exclusion criteria outlined above. A stratified random sample will be created from these hospitals with the goal of achieving a representative selection of facilities. Four strata will be formed based on predetermined hospital sizes, measured by the number of beds (small facilities ≙ < 250 beds; medium-sized facilities ≙ 250–399 beds; large facilities ≙ 400–599 beds; very large facilities ≙ > 600 beds). The random selection of hospitals will be conducted using an online software tool (https://www.random.org/sequences). Given that not all facilities will participate in the study, more than the planned 30 hospitals will be initially selected. Subsequently, nursing directors from 60 institutions will be contacted via email and followed up with a phone call. If requested, the study will be presented in person at the hospital.

According to hospital sizes, two to eight wards will be included from each facility (small facilities: n = approximately 2 wards; medium-sized facilities: n = approximately 3 wards; large facilities: n = approximately 5 wards; very large facilities: n = 8 wards). The selection of wards will be made by the nursing directors of the participating hospitals, who will be asked to make their selections as randomly as possible in order to avoid selection bias.

On the participating wards, all patients who meet the inclusion and exclusion criteria and are present at the time of data collection will be included. Recruitment will be carried out by pre-designated contact persons on the wards (usually the ward managers). These contact persons will be informed about the study in advance by the research team. Additionally, all nursing professionals working on the participating wards who meet the inclusion and exclusion criteria will be invited to participate in the study. Contact persons will be asked to encourage colleagues who are interested to get in touch with the research team.

To increase both the willingness to participate at the hospital and ward levels, as well as to enhance the response rate, various strategies will be applied (Edwards et al., 2009). These include maintaining close personal contact with the contact persons at the participating hospitals, announcing the provision of structured feedback on the study results for all participating hospitals, and holding a lottery for five cash prizes with a total value of 1,000 euros among the participating wards, regardless of the individual response rates. The random selection of the cash prize winners will be conducted using the same randomisation software (https://www.random.org/sequences) that was used for the sample selection

Data Collection

The data collection consists of a paper-based survey (questionnaires) from patients and nursing professionals.

Instruments

To assess sleep problems and subjective sleep quality in patients, the "Consensus Sleep Diary" (CSD), items from the "Sleep Disturbance Item Bank" of the Patient Reported Outcomes Measurement Information System (PROMIS), and the "Richards-Campbell Sleep Questionnaire" (RCSQ) are used. According to Buysse, Reynolds, Monk, Berman, and Kupfer's (1989) definition of "sleep quality," both qualitative and quantitative aspects of sleep are collected. The three instruments are shown in Appendices 1 to 4.

The "Consensus Sleep Diary" (CSD) (Carney, Buysse, Ancoli-Israel, Edinger, Krystal, Lichstein, & Morin, 2012) is a consensus-based, standardised questionnaire in the form of a sleep diary. It is suitable for both short-term and long-term use. The goal of the CSD is to capture the quantitative dimensions of

sleep quality. The core version contains eight items. These include questions about the time of going to bed and falling asleep, the duration to fall asleep, the frequency, duration, and last time of waking up, the wake-up time, and the overall perceived sleep quality. Additionally, there is a free-text field for comments. In addition to the core CSD, there are extended versions of the instrument with optional additional items. Two of these items, which address napping and the use of sleep medications, will be included in the data collection due to their relevance to the study. The CSD is not available in German. A translation of the instrument, following Beaton, Bombardier, Guillemin, and Ferraz (2000), is in preparation.

The "Sleep Disturbance Item Bank" from the Patient Reported Outcomes Measurement Information System (PROMIS) (Buysse et al., 2010) aims to capture qualitative aspects of sleep. The items are presented as statements and are evaluated on a five-point Likert scale ("not at all" to "very") according to how well they match the current situation. The questions in the "Sleep Disturbance Item Bank" have already been used and validated in various patient groups, showing excellent measurement properties (ibid.). In addition to the original 27-item version, there are four shortened versions of the instrument. Based on aspects of practicality and the relevance of the items for this study, the "Sleep Disturbance Item Bank – Short Form 8a" with eight items will be used. This includes areas such as sleep quality, sleep problems, and sleep-related recovery. The "Sleep Disturbance Item Bank" has already been fully translated into German. The German version is currently being obtained through the PROMIS website (http://promis-germany.de/instrumente/).

The "Richards-Campbell Sleep Questionnaire" (RCSQ) (Richards, 1987) is a widely used and frequently employed questionnaire to assess sleep and sleep quality in critically ill patients. The five items collect information about sleep depth, falling asleep, waking up, re-sleeping, and sleep quality. The assessment is based on a visual analog scale. The RCSQ is already available in a German version. A subsequent review of internal consistency following the translation process showed satisfactory test results (Krotsetis, Richards, Behncke, & Köpke, 2017).

To compare hospital sleep problems and subjective sleep quality with usual sleep, each item of the previously described instruments will be asked twice. The average sleep at home during the month prior to the hospital stay and the sleep in the hospital the night before data collection will be evaluated. Considering the abstract nature of asking about average sleep during a one-month period, participants will also be asked to indicate how confident they are in their responses. Response options will be on a four-point Likert scale ("not confident at all" to "very confident"). This additional information will help assess data quality.

The items to assess sleep problems and subjective sleep quality will be supplemented with three additional questions about hospital-specific factors that are believed to be related to sleep problems.

Participants will be asked to select the factors associated with falling asleep, waking up at night, and waking up in the morning during the previous night in the hospital. Possible factors will be presented in a list. Additionally, a free-text field will allow participants to add other relevant factors. For all three questions, multiple selections will be allowed. The items regarding hospital-specific factors were successfully used by Wesselius et al. (2018). The original questions are shown in Appendix 5.

To identify currently used strategies and measures to promote sleep, a questionnaire (Appendix 6) will be used, which contains both quantitative and qualitative elements. The questionnaire was developed by the research team based on the current state of research (e.g., Eliassen & Hopstock, 2011; Kauffmann, Heinemann, Himmel, Hußmann, Schlott, & Weiß, 2018; Redeker et al., 2011; Salzmann-Erikson, Lagerqvist & Pousette, 2015).

In the first part of the questionnaire, lists of possible strategies and measures to promote sleep will be presented. Respondents will be asked to select those they know, those that are currently implemented, and those that they would like to be implemented. Free-text fields allow for additional answer options. In the second part of the questionnaire, open-ended questions will be asked about the current implementation of strategies and measures to promote sleep in clinical practice. Finally, a list of statements will assess the importance of sleep promotion and the role of nursing professionals in initiating and implementing strategies and measures. These will be evaluated using a four-point Likert scale ("strongly disagree" to "strongly agree").

Before the data collection, cognitive pre-testing of the developed questionnaire will be conducted. Five nursing professionals who meet the inclusion and exclusion criteria will be recruited through the professional network of the researchers. Necessary adjustments to the questionnaire will be made based on the feedback.

In addition to the instruments mentioned above, a number of individual items will be integrated into the data collection for the purpose of sample description and later data analysis. Patient data on sociodemographic (age and gender) and clinical information (department, admission diagnosis, surgery, admission date and type, number of roommates, and sleep medications) will be collected. Data on age, gender, department, professional qualification, and experience will be collected from participating nursing professionals.

Procedure

In the first step, the entire study documentation will be handed over by the researchers to the designated contact persons (usually ward managers). These contact persons will coordinate the data

collection on the participating wards and will be responsible for distributing and collecting the study materials.

All patients who meet the inclusion and exclusion criteria will receive a package of questionnaires, which includes the instruments to assess sleep problems, subjective sleep quality, hospital-specific factors, and individual items on age and gender. The ward managers (or their deputies) participating in the survey will receive a questionnaire package that contains the instrument to identify strategies and measures to promote sleep, as well as individual items related to the sociodemographic characteristics of the respondent. In addition, all participants will receive written study information, a consent form, and an envelope from the contact persons. Participants will be asked to place the completed study materials in the envelope and seal it. All patients who have received the study materials will be reminded once by the contact persons to participate in the study. The sealed envelopes will be handed over to the contact persons either on the same day or the following day and then delivered to the researchers.

The envelopes will have code numbers to allow for matching the study material to the hospital (positions 1 and 2) and ward (positions 3 and 4). The fifth and sixth digits of the code on the patient envelopes will also temporarily identify the respondents. A code list will be maintained on the participating wards, enabling the matching of code numbers to respondents. The code list will be used to allow contact persons to complete a separate questionnaire sheet with clinical characteristics of the participating patients, based on the hospital records, and then assign it to the rest of the study material. The code list will also provide contact persons the ability to document the distribution and collection of questionnaires using a protocol. The study team will never have access to the code list. The contact persons will not have access to the completed questionnaires stored in sealed envelopes. The contact persons will be asked to destroy the code list immediately after data collection. From that point on, study materials can only be linked to the facility and ward. The corresponding code list is accessible only to the study team.

Data Management

Once the questionnaire packages arrive at the study center, the envelopes will be destroyed. The code numbers will be noted on the questionnaire packages for subsequent data analysis and ward-specific feedback. The study data will then be transferred into the SPSS software program by the researchers. During this process, initial plausibility checks will be carried out.

The collected data will be securely stored at the study center in anonymised form for patients and pseudonymised form for ward managers. Identifying information related to the participating hospitals and wards, which could link to the participating ward managers, will be kept separate from the rest of

the study material. Further data processing will take place in a protected physical and IT-supported environment at the study center.

Data Analysis

The data from the surveys will initially be analysed descriptively. Categorical variables will be presented in absolute and relative frequencies. The other variables will be described using the relevant location and dispersion parameters according to their respective scale levels. In addition, the data will be analysed using various inferential statistical methods (e.g., paired t-test or McNemar test for comparing sleep before and during the hospital stay, as well as regression analyses to investigate relationships). Possible cluster effects will be taken into account. The entire data analysis will be carried out using the SPSS statistical software.

Open-ended questions and free-text fields in the questionnaires will be evaluated using qualitative content analysis (Mayring, 2010). Category formation will be based on a combination of a deductive and inductive approach.

PART II: ACCEPTANCE AND EFFECTIVENESS OF DIFFERENT INFORMATION LETTERS FOR PATIENT RECRUITMENT IN HOSPITALS

Background

A key component of the empirical research process is the recruitment of participants. In this context, recruitment refers to identifying potential research participants, ensuring an adequate and/or representative sample, and retaining participants until the study's conclusion, while simultaneously considering economic and ethical aspects (Blanton, Morris, Prettyman, McCulloch, Redmond, Light & Wolf, 2006). Researchers have access to a variety of recruitment strategies, techniques, and methods (Heerman et al., 2017; Ngune, Jiwa, Dadich, Lotriet & Sriram, 2012).

Participant recruitment is often described as a barrier to conducting scientific studies. Published reviews show that in the majority of empirical studies, the originally intended sample size was not reached, or not within the proposed time frame (Bower, Wilson & Mathers, 2007; McDonald et al., 2006). Failure to ensure an adequate sample size can affect various aspects of a study, such as meeting the time and financial framework, internal and external validity, and dissemination of research findings (Bower, Brueton, Gamble, Treweek, Smith, Young & Williamson, 2014; Ngune et al., 2012; Treweek et al., 2013). At the same time, empirical data guiding the recruitment process in studies is currently very limited. Existing insights mainly come from qualitative case studies. These provide initial evidence that successful recruitment of participants is influenced by a variety of factors across different recruitment elements (Bower et al., 2014).

One element in the recruitment phase is the communication of study information (Bower et al., 2014). While oral and written study information are typically used to obtain informed consent and adequately inform potential participants about the goals, methods, expected results, and potential risks of the study, the use of traditional study information letters has been identified as a potential barrier to recruitment. Written study information is often described as burdensome due to its length and complexity (Antoniou, Draper, Reed, Burls, Southwood & Zeegers, 2011; Bower et al., 2014). These findings have led to initial recommendations regarding the targeted design and appropriate scope of study information for specific target groups (Bower et al., 2014). However, empirical investigations that build on these recommendations and specifically examine different recruitment approaches in terms of their acceptance and effectiveness are still lacking. Furthermore, it is not yet clear how different groups of people respond to recruitment strategies, and there is a lack of guidance on the sociodemographic diversity expected from a specific approach. This information is especially relevant when aiming to capture a broad spectrum of research participants in a sample or deliberately include certain groups (Gaertner, Seitz, Fuchs, Busch, Holzhausen, Martus & Scheidt-Nave, 2016; Galea &

Tracy, 2007). The extent to which the design of study information corresponds to the participant characteristics has not been investigated yet.

Objectives

Although ensuring an adequate sample plays a significant role in the research process, the current understanding of participant recruitment remains limited. Based on the existing research, this study aims to investigate two recruitment approaches that differ in the design of written study information. The objective of this research is to assess the acceptance and effectiveness of an attractively designed study information flyer compared to a formal study information letter for recruiting participants in a questionnaire study on sleep in hospitals.

It is expected that this study will provide insights into the acceptance and effectiveness of both written study information approaches. These findings will contribute to the development of concrete recommendations for designing components of the empirical research process, thereby facilitating the successful generation and dissemination of scientific knowledge.

Methodology

Study Design

To compare two written study information approaches, an embedded cluster-randomised study (SWAT) will be conducted as part of the cross-sectional observational study on hospitalised patients' sleep described in Part I of this protocol. Patients from randomly selected hospitals near the study center will be assigned to two study groups based on cluster randomisation. These groups will differ in the written study information provided. The data collection and evaluation regarding the acceptance and effectiveness of both recruitment approaches will use both qualitative and quantitative methods.

Sample

The sample will consist of the institutions, wards, and patients that agree to participate in the research described in Part I of this protocol. For the second part of the research, a cluster randomisation will be performed to form two study groups. A cluster is defined as a single participating ward within the hospitals, including all patients admitted during the data collection period who meet the inclusion and exclusion criteria. The randomisation will be done by an external person using computer-generated randomisation lists, stratified by hospital, so that a separate randomisation list is created for each institution.

Recruitment

Information about the recruitment of institutions and wards can be found in Part I of this protocol (p. 3ff). For patient recruitment, researchers will work through intermediary contacts (usually ward managers) at the participating wards. These contact persons will personally approach all patients who meet the inclusion and exclusion criteria and provide them with initial study information. Prior to this, the contact persons will be prepared by the researchers. If a patient expresses interest in participating in the study, they will be given the study materials. Depending on the group assignment, the written study information will differ. One group will receive a classic multi-page information letter (Appendix 7), while the other group will receive a study information flyer (Appendix 8).

The design of the written study information follows available recommendations. In the classic information letter, the first part presents general information about the essential elements of the study (background and goals, procedures, and benefits and risks), while the second part provides detailed information about data protection (National Research Ethics Service, 2009). The flyer presents the study information in a reduced and more attractive format (Antoniou et al., 2011). To integrate another communication channel (Huang, Bull, Johnston McKee, Mahon, Harper, Roberts & Team, 2018), the flyer includes a QR code or link that leads to a video where the researchers introduce themselves and the planned research project. For both types of study information, simple, easy-to-understand language will be used (Bower et al., 2014).

Data Collection

To assess the acceptance of the provided written study information, participating patients will be asked to complete a specially developed questionnaire (Appendix 9). This includes closed questions that rate predefined areas such as clarity, completeness, and appropriateness of the study information using a numerical scale. The questionnaire also includes open-ended questions to identify successful or less successful elements and to pinpoint areas for improvement.

The questionnaire was developed by the research team based on existing literature. A pretest will be conducted before its use in data collection. Five individuals who meet the inclusion and exclusion criteria and five subject matter experts will be recruited for the pretest. Adjustments to the questionnaire will be made if necessary.

In addition to the data collected for Part I of the study protocol (age and gender), further sociodemographic data will be collected. These will include socioeconomic status (highest educational level and employment status) and cultural background (nationality, country of birth, and native language). The developed questionnaire and corresponding items on participant characteristics will be included in the provided questionnaire package.

Furthermore, the contact persons on the wards will record reasons for patient non-participation in the study, and researchers will gather information on the frequency of video views.

Data Analysis

For both recruitment approaches, the response rate (number of patients who participate in the study/number of patients asked to participate) will be calculated. Additionally, a descriptive analysis of the sociodemographic characteristics of participants in both study groups will be performed, using frequencies and location and dispersion parameters. The data will also be analysed using various inferential statistical methods (e.g., comparison of sociodemographic distribution through Chi-square test or ANOVA). Data analysis will be conducted using SPSS statistical software. Open-ended questions and free-text fields in the questionnaire will be analysed using qualitative content analysis (Mayring, 2010). People involved in data analysis will be blinded to the cluster group assignments.

ETHICAL CONSIDERATIONS

Enlightenment and Consent

The objectives and content of the research projects will initially be presented to the nursing management of the selected hospitals. They will facilitate access to the participating wards and respective contact persons (Parts I and II). Both will also be informed about the research project by the research team. For Parts I and II of the present study protocol, the contact persons on the wards will take over the selection and enlightenment of the patients. Additionally, the contact persons will be asked to reach out to the research team if they are personally interested in participating or if their colleagues express interest.

Both verbal and written information about the study will be provided to all participants. In the context of informed consent, it will be emphasised that participation in the research project is voluntary and can be declined or revoked at any time without providing reasons, with no consequences. After receiving the information, patients and nursing staff will be given an adequate amount of time to consider. The contact details of the research team provided in the information letters will also ensure the possibility of consulting in case of open questions.

If there is interest in participating in the study, written consent will be obtained for the research projects presented in Parts I and II of this study protocol. A copy of the consent form will remain with the participants.

Potential Risks

To assess potential risks associated with participation in the study, a distinction is made between the surveys of nursing staff and patients.

Nursing Staff

Part I

Nursing staff, in their role as study participants, do not generally represent a vulnerable group. This is because no personal or sensitive information will be requested in the planned research projects, but rather only professional everyday experience. However, participation in the study means a time commitment, which could be a potential burden, such as role pressure. It is important to emphasise that the surveys will only take a limited time (roughly 15 to 20 minutes once). Furthermore, the surveys will be conducted in the hospitals where the participants work, thus avoiding additional time expenditure (e.g., due to travel). The nursing staff can also individually decide on a convenient time frame for completing the questionnaires. Additionally, they can decline or withdraw from participation

at any time. To prevent any potential conscious or unconscious influence by their supervisors, the nursing staff will be informed in advance that their supervisors will not be notified about declined participation specific to individuals or wards.

There is also the possibility that participation in the study may have a positive effect on the nursing staff. Their involvement in the research project can be perceived as a form of appreciation, as their perspective is acknowledged and their professional experiences are given attention.

Hospital Patients

Part I

Hospital patients, on the other hand, are generally considered a vulnerable group. A hospital stay is an exceptional situation and is often associated with existential threats as well as a decrease in well-being. Participation in the study, in the form of a written self-report on sleep problems and subjective sleep quality, may pose an additional burden. On the one hand, it involves a certain (time) commitment. On the other hand, patients are asked to actively engage with a potential issue, which could worsen an already threatening situation. Patients whose current condition does not permit participation will be excluded from the study from the outset. The selected instruments have already been proven suitable for use in acute hospitals in previous research (Krotsetis et al. 2017; Wesselius et al., 2018). The structured format and brevity (approximately 15 to 20 minutes in total) of the questionnaires are intended to reduce the burden on participating patients. Furthermore, the timing of filling out the questionnaires can be chosen and managed flexibly, ensuring that no disruption to the care structures and processes is expected from participation.

At the same time, participation in the study could contribute to the relief and improvement of the patients' well-being, as it provides them with the opportunity to report on their situation and possible problems. Asking for their perspective can also make patients feel recognised and taken seriously, which in turn can have a positive impact on their satisfaction.

If, despite the above, the participation results in a burden for the patients, they have the right to skip questions, interrupt the survey, or stop it entirely. Given the dependency relationship of the patients, it will be emphasised beforehand that their participation will not affect their care in any way. To ensure this, sealed envelopes will be used for data collection. This way, it will not be evident to the contact persons on the wards whether the study material has been completed. After the questionnaires are collected, the envelopes also prevent the contents from being accessed or attributed to specific individuals. The code list and questionnaires will be stored separately at all times.

The overarching goal of the study is to generate insights on the topics of sleep, sleep problems, and sleep quality of hospital patients. This is essential in order to reflect the current situation and, subsequently, derive potential needs. Considering this, when evaluating the benefit and harm of the research project, the long-term benefit outweighs the risk of causing short-term harm to the participants, which is actively prevented and appropriately addressed in case of doubt.

Part II

It is not anticipated that participation in the study, in the form of receiving different written study information, poses any risks to the participants. Both the traditionally designed information letter and the information flyer contain all relevant core information about the research project, ensuring that informed consent is obtained in any case. If potential participants feel insufficiently informed despite both verbal and written information, they can additionally contact the contact persons on the wards or the researchers themselves. Furthermore, as described for Part I, potential participants will be informed that participation can be declined or revoked at any time without consequences.

Given the research goal of investigating more promising and less burdensome study information for the research process and potential participants, the long-term benefit of the planned research project can be assumed.

Data Protection

Throughout the study, the currently applicable data protection regulations of the European General Data Protection Regulation (GDPR) in accordance with Art. 6 Para. 1 lit. a, 7, and 9 Para. 2 lit. a, as well as the Federal Data Protection Act (BDSG), will be strictly adhered to.

In accordance with the GDPR, Art. 4 Para. 1 and Art. 9 Para. 1, personal data or special categories of personal data will be collected, processed, and used in the research projects presented in Parts I and II. These data are considered to be of special protection. Therefore, participation in the research projects requires the informed consent of patients and nursing staff in writing, which explicitly relates to the aforementioned types of data. Only with this consent will the described data collection take place. If consent is withdrawn, participation in the study will no longer be possible. Until the destruction of the code lists, which link identifying data and pseudonyms, participants have the opportunity to withdraw their previously given consent.

The data collection and analysis are subject to the principle of strict confidentiality, which is assured to participants before the study begins. All data will only be used for scientific purposes and in accordance with informed consent. Participants will be informed that the data obtained in the study

will be published in scientific publications. In doing so, the data will be processed in a way that makes it impossible to trace back to individual people or institutions. According to the consent, the researchers also reserve the right to reuse the collected data in future research projects, such as secondary data analyses.

Until the completion of data collection and the personal handover of study materials to the researchers, the questionnaires and code lists will be stored separately and securely by the contact person for other people, in locked cabinets on the participating wards. Afterward, all data collected in the course of the research project will be securely stored in a protected IT environment (electronic data) or in locked cabinets (paper-based data) at the Institute of Nursing Science at the University of Cologne. These will only be accessible to project staff. The patient data collected in Parts I and II will be anonymised for the researchers. The data from the surveys of nursing staff in Part I, however, will be pseudonymised. Identifying information regarding the participating hospitals and wards, which could lead to conclusions about the participating ward managers, will be stored separately from the rest of the study material. After completion of data analysis, the data will be anonymised by destroying the corresponding code list.

The collected data will be stored by the University of Cologne for a period of ten years and then deleted.

REFERENCES

Antoniou, E., Draper, H., Reed, K., Burls, A., Southwood, T. & Zeegers, M. (2011). An empirical study on the preferred size of the participant information sheet in research. *J Med Ethics, 37*, 557-562.

Beaton, D. E., Bombardier, C., Guillemin, F. & Ferraz, M. B. (2000). Guidelines for the process of cross-cultural adaptation of self-report measures. *Spine (Phila Pa 1976), 25*(24), 3186-3191.

Blanton, S., Morris, D. M., Prettyman, M. G., McCulloch, K., Redmond, S., Light, K. E. & Wolf, S. L. (2006). Lessons learned in participant recruitment and retention: the EXCITE trial. *Phys Ther, 86*(11), 1520-1533.

Bower, P., Brueton, V., Gamble, C., Treweek, S., Smith, C. T., Young, B. & Williamson, P. (2014). Interventions to improve recruitment and retention in clinical trials: a survey and workshop to assess current practice and future priorities. *Trials, 15*, 399.

Bower, P., Wilson, S. & Mathers, N. (2007). Short report: How often do UK primary care trials face recruitment delays? *Family Practice, 24*(6), 601-603.

Buysse, D. J., Reynolds, C. F., Monk, T. H., Berman, S. R. & Kupfer, D. J. (1989). The Pittsburgh Sleep Quality Index: a new instrument for psychiatric practice and research. *Psychiatry Res, 28*(2), 193-213.

Buysse, D. J., Yu, L., Moul, D. E., Germain, A., Stover, A., Dodds, N. E., . . . Pilkonis, P. A. (2010). Development and validation of patient-reported outcome measures for sleep disturbance and sleep-related impairments. *Sleep, 33*(6), 781-792.

Carney, C. E., Buysse, D. J., Ancoli-Israel, S., Edinger, J. D., Krystal, A. D., Lichstein, K. L. & Morin, C. M. (2012). The consensus sleep diary: standardizing prospective sleep self-monitoring. *Sleep, 35*(2), 287-302.

Edwards, P. J., Roberts, I., Clarke, M. J., Diguiseppi, C., Wentz, R., Kwan, I., . . . Pratap, S. (2009). Methods to increase response to postal and electronic questionnaires. *Cochrane Database Syst Rev*(3), MR000008.

Eliassen, K. M. & Hopstock, L. A. (2011). Sleep promotion in the intensive care unit-a survey of nurses' interventions. *Intensive Crit Care Nurs, 27*(3), 138-142.

Friese, R. S. (2008). Sleep and recovery from critical illness and injury: a review of theory, current practice, and future directions. *Crit Care Med, 36*(3), 697-705.

Gaertner, B., Seitz, I., Fuchs, J., Busch, M. A., Holzhausen, M., Martus, P. & Scheidt-Nave, C. (2016). Baseline participation in a health examination survey of the population 65 years and older: who is missed and why? *BMC Geriatr, 16*, 21.

Galea, S. & Tracy, M. (2007). Participation rates in epidemiologic studies. *Ann Epidemiol, 17*(9), 643-653.

Ganz, F. D. (2012). Sleep and immune function. *Crit Care Nurse, 32*(2), e19-25.

Gellerstedt, L., Medin, J. & Rydell Karlsson, M. (2014). Patients' experiences of sleep in hospital: A qualitative interview study. *J Res Nurs, 19*(3), 176-188.

Heerman, W. J., Jackson, N., Roumie, C. L., Harris, P. A., Rosenbloom, S. T., Pulley, J., . . . Kripalani, S. (2017). Recruitment methods for survey research: Findings from the Mid-South Clinical Data Research Network. *Contemp Clin Trials, 62*, 50-55.

Hoevenaar-Blom, M. P., Spijkerman, A. M., Kromhout, D. & Verschuren, W. M. (2014). Sufficient

sleep duration contributes to lower cardiovascular disease risk in addition to four traditional lifestyle factors: the MORGEN study. *Eur J Prev Cardiol, 21*(11), 1367-1375.

Huang, G. D., Bull, J., Johnston McKee, K., Mahon, E., Harper, B., Roberts, J. N. & Team, C. R. P. (2018). Clinical trials recruitment planning: A proposed framework from the Clinical Trials Transformation Initiative. *Contemp Clin Trials, 66*, 74-79.

John, M. E., Edet, O., Mgbekem, M., Robinson-Bassey, Duke, Esienumoh, E. & Ndebbio. (2007). Sleep disturbance among patients in hospital: implications for nursing care. *West African Journal of Nursing, 18*, 42-48.

Kauffmann, L., Heinemann, S., Himmel, W., Hußmann, O., Schlott, T. & Weiß, V. (2018). Nicht-medikamentöse Maßnahmen bei Ein- und Durchschlafproblemen von älteren Patienten im Krankenhaus – Qualitative Interviews mit Pflegenden. *Pflege, 31*, 1-10.

Krotsetis, S., Richards, K. C., Behncke, A. & Köpke, S. (2017). The reliability of the German version of the Richards Campbell Sleep Questionnaire. *Nurs Crit Care, 22*(4), 247-252.

Lane, T. & East, L. A. (2008). Sleep disruption experienced by surgical patients in an acute hospital. *Br J Nurs, 17*(12), 766-771.

Lange, T., Dimitrov, S. & Born, J. (2010). Effects of sleep and circadian rhythm on the human immune system. *Ann N Y Acad Sci, 1193*, 48-59.

Mayring, P. (2010). *Qualitative Inhaltsanalyse: Grundlagen und Techniken* (11 ed.). Weinheim: Beltz.

McDonald, A. M., Knight, R. C., Campbell, M. K., Entwistle, V. A., Grant, A. M., Cook, J. A., . . . Snowdon, C. (2006). What influences recruitment to randomised controlled trials? A review of trials funded by two UK funding agencies. *Trials, 7*, 9.

National Research Ethics Service. (2009). *Information sheets and consent forms: guidance for researchers and reviewers*. Retrieved from http://www.nres.npsa.nhs.uk

Ngune, I., Jiwa, M., Dadich, A., Lotriet, J. & Sriram, D. (2012). Effective recruitment strategies in primary care research: a systematic review. *Qual Prim Care, 20*(2), 115-123.

Pellatt, G. C. (2007). The nurse's role in promoting a good night's sleep for patients. *Br J Nurs, 16*(10), 602-605.

Pilkington, S. (2013). Causes and consequences of sleep deprivation in hospitalised patients. *Nurs Stand, 27*(49), 35-42.

Radtke, K., Obermann, K. & Teymer, L. (2014). Nursing knowledge of physiological and psychological outcomes related to patient sleep deprivation in the acute care setting. *Medsurg Nurs, 23*(3), 178-184.

Rasch, B. & Born, J. (2013). About Sleep’s Role in Memory. *Physiol Rev, 93*, 681-766.

Raymond, I., Nielsen, T. A., Lavigne, G., Manzini, C. & Choiniere, M. (2001). Quality of sleep and its daily relationship to pain intensity in hospitalized adult burn patients. *Pain, 92*(3), 381-388.

Redeker, N. S., Hedges, C. & Booker, K. J. (2011). Sleep in Adult Acute and Critical Care Settings. In N. S. Redeker & G. Phillips McEnany (Eds.), *Sleep Disorders and Sleep Promotion in Nursing Practice* (pp. 321-338). New York: Springer Publishing Company.

Richards K. (1987). Techniques for measurement of sleep in critical care. *Focus Crit Care*, *14*(4), 34-40.

Salzmann-Erikson, M., Lagerqvist, L. & Pousette, S. (2015). Keep calm and have a good night: nurses' strategies to promote inpatients' sleep in the hospital environment. *Scand J Caring Sci, 30*(2), 356-364.

Stone, K. L., Ensrud, K. E. & Ancoli-Israel, S. (2008). Sleep, insomnia and falls in elderly patients. *Sleep Med, 9*, 18-22.

Treweek, S., Lockhart, P., Pitkethly, M., Cook, J. A., Kjeldstrom, M., Johansen, M., . . . Mitchell, E. D. (2013). Methods to improve recruitment to randomised controlled trials: Cochrane systematic review and meta-analysis. *BMJ Open, 3*(2).

Weinhouse, G. L., Schwab, R. J., Watson, P. L., Patil, N., Vaccaro, B., Pandharipande, P. & Ely, E. W. (2009). Bench-to-bedside review: delirium in ICU patients - importance of sleep deprivation. *Crit Care, 13*(6), 234.

Wesselius, H. M., van den Ende, E. S., Alsma, J., Ter Maaten, J. C., Schuit, S. C. E., Stassen, P. M., . . . Onderzoeks Consortium Acute Geneeskunde" Acute Medicine Research, C. (2018). Quality and Quantity of Sleep and Factors Associated With Sleep Disturbance in Hospitalized Patients. *JAMA Intern Med, 178*(9), 1201-1208.

Appendix

Appendix 1: Consensus Sleep Diary (CSD) – Core Version

This appendix provides additional details relevant to the host study (Part I).

Appendix 2: Consensus Sleep Diary (CSD) – Additional Items

This appendix provides additional details relevant to the host study (Part I).

Appendix 3: Sleep Disturbance Item Bank – Short Form 8a

This appendix provides additional details relevant to the host study (Part I).

Appendix 4: Richards-Campbell Sleep Questionnaire (RCSQ)

This appendix provides additional details relevant to the host study (Part I).

Appendix 5: Questions on Hospital-Specific Factors

This appendix provides additional details relevant to the host study (Part I).

Appendix 6: Questionnaire for Identifying Currently Applied Strategies and Measures for Sleep Promotion

This appendix provides additional details relevant to the host study (Part I).

Appendix 7: Information and Consent Letter for Patients (Part I and II)

Sleep Acute

Scientific study to analyse patients' sleep during inpatient hospital care

Responsible according to §4 Abs. 7 DS-GVO:

Institute for Nursing Science, Faculty of Medicine, University of Cologne
Prof. Dr phil. Sascha Köpke
Gleueler Straße 176-178
50935 Cologne
Phone: 0221 478 51658
E-mail: sascha.koepke@uk-koeln.de

Information letter for patients

Dear Patient,

this letter provides information about the objectives and procedures of the study mentioned above. The study is being conducted by the Institute of Nursing Science, University of Cologne. **Your participation in the study is voluntary**.

Please read the following text carefully. If you have any questions, feel free to speak with your ward supervisor or contact us directly. You will find our contact details at the end of this letter.

I. Information about the study

Background and Objectives

Sleep is a state essential for health, well-being and daily functioning. While illness often increases the need for rest and sleep, previous scientific studies suggest that sleep problems are common during hospital stays and are associated with various risks.

The goal of the "Sleep Acute" study is to learn more about patients' sleep and potential sleep issues during their hospital stay. Additionally, the study aims to identify strategies for promoting better sleep.

At the same time, we are analysing the acceptability and effectiveness of the written informational materials used in this study. This will help us to gain valuable insights into designing informational materials for future scientific studies.

Potential Participants

You can participate in the study if you are an adult and have spent at least two nights in the hospital on a general ward (i.e., not a monitoring or intensive care unit) at the time of data collection.

Procedures

If you decide to participate in the study, you will receive two questionnaires along with this written study information. One questionnaire will ask about your sleep in hospital and at home, enabling us to compare your sleep during your hospital stay with your usual sleep patterns. The other questionnaire will ask for your feedback on this information letter. Additionally, you will be asked for some personal details (e.g., age and gender).

Answering the questions will take around 15 to 20 minutes in total. Please place the completed questionnaires in the enclosed envelope and seal it. The sealed envelopes will be collected by staff at your ward the day after they have been distributed and then handed over to the researchers.

Additionally, information about your hospital stay (such as admission diagnosis, medical department, surgeries, admission date and type, number of roommates, and use of sleep medications) will be required. To minimise the time needed for your participation, these details will be gathered by staff from your ward.

Benefits and Risks

Participation in the study does not offer any personal benefits for you. However, it can contribute to a better understanding of sleep during hospital stays and the use of informational materials in studies. The insights gained will help identify needs and areas for improvement. You will not receive individual feedback about the scientific results of the study.

Participation in the study is usually not associated with any significant risks, but a minimal burden cannot be completely ruled out. However, this risk is considered to be very low.

II Data Protection

Study-Specific Information

(1) Data Processing and Usage

In this study, personal data (i.e., personal and health-related information) as defined by Art. 4 para. 1 and Art. 9 of the EU General Data Protection Regulation (DS-GVO) will be collected, processed, and used in paper form. The use of your data is based on legal provisions (pursuant to Art. 6 para. 1(a), Art. 7, and Art. 9 para. 2(a) DS-GVO) and requires your consent before participating in the study.

All information collected about you during the study will initially be pseudonymised (i.e., without mentioning names or identifiable data, using an assigned code of numbers and letters). A coding list, which links full names with pseudonyms, allows the questionnaires to be matched to individuals. However, the use of sealed envelopes ensures that identifying data and questionnaires are always stored separately. Only

one staff member from your ward will have access to the coding list. They will be instructed to permanently destroy the coding list immediately after data collection is complete. From that point onward, the data will be anonymised (i.e., the data can no longer be linked to any specific person).

The sealed envelopes you provide will only be opened by the researchers once the study materials arrive at the study center. All information collected about you during the study will be securely stored and protected from unauthorised access. The anonymised data will be stored and analysed at the Institute of Nursing Science, University of Cologne.

All data will be used solely for scientific research purposes and in accordance with your informed consent. The collected data is intended for use in publications, but you will not be identifiable as a participant. Additionally, the data collected about you in this study may be reused in future research projects (known as "secondary data analyses") and shared in anonymised form with national and international collaborators, both within and outside the University of Cologne.

(2) Duration of Data Storage

The collected data will be stored by the University of Cologne for a period of ten years and will then be deleted.

(3) Right to Withdraw and Data Removal

Participation in the study is voluntary. You can withdraw from the study at any time without providing a reason. This will not result in any disadvantages for you. Upon request, your data will be corrected, anonymised, or deleted. You also have the right to object to further processing of your data or to demand limitations on its use. You can address your objection to the ward supervisor and/or the researchers, either verbally or in writing.

If the data has already been anonymised, it can no longer be linked to you. Therefore, please note that anonymised data and data used in scientific analyses cannot be deleted upon request.

General Information

(1) The responsible parties according to Art. 4 para. 7 DS-GVO are:

University of Cologne
Gesa Diekmann and Alexander May
Albertus-Magnus-Platz
50923 Cologne
Phone: 0221 470 3872
E-mail: dsb@verw.uni-koeln.de
Website: https://verwaltung.uni-koeln.de/stabsstelle02.3/

You can contact our Data Protection Officer using the contact details provided above or our postal address (see above) with the addition "Data Protection Officer."

(2) You have the right to lodge a complaint with a data protection supervisory authority regarding the processing of your personal data within our organisation.

The State Commissioner for Data Protection and Freedom of Information North Rhine-Westphalia:

Helga Block
Kavalleriestraße 2-4
40213 Düsseldorf
Phone: 0211 384240
E-mail: poststelle@ldi.nrw.de
Website: https://www.ldi.nrw.de

(3) If you have any concerns, questions, or complaints regarding data processing and the compliance with data protection regulations, you may of course contact the study director at any time. The contact details can be found at the end of this letter.

(4) The study management will take all reasonable steps to ensure the protection of your data in accordance with the General Data Protection Regulation and other laws. The data will be protected from unauthorised access. Personal data will be anonymised as soon as possible according to the research purpose, unless legitimate interests of the data subject prevent this. Until then, the data will be stored separately with the identifiers that allow personal or material details to be attributed to a specific or identifiable person. These identifiers will only be merged with the individual data when necessary for the research purpose.

(5) The responsible party will only publish personal data if you have expressly consented to such publication.

(6) You have the right to withdraw your data protection consent at any time. The withdrawal of consent will not affect the legality of the processing carried out based on the consent until the withdrawal.

III Contact Study Team

Institute of Nursing Science, Faculty of Medicine, University of Cologne
Prof. Dr phil. Sascha Köpke
Gleueler Straße 176-178
50935 Cologne
Phone: 0221 478 51658
E-mail: sascha.koepke@uk-koeln.de

Sleep Acute

Scientific study to analyse patients' sleep during inpatient hospital care

Responsible according to §4 Abs. 7 DS-GVO:

Institute for Nursing Science, Faculty of Medicine, University of Cologne
Prof. Dr phil. Sascha Köpke
Gleueler Straße 176-178
50935 Cologne
Phone: 0221 478 51658
E-mail: sascha.koepke@uk-koeln.de

Informed Consent for Patients

____ (Name of the person providing information) provided me ____ (Name of the participating person) with information about the nature, scope and significance of the above-mentioned study on __________________ (Date). All my questions were answered satisfactorily.

The study "Sleep Acute" aims to learn more about sleep during inpatient hospital care. Based on this, the study intends to identify needs and derive approaches to improve the current care situation. Additionally, the acceptability and effectiveness of the study's informational materials will be examined. This will help to develop recommendations for recruiting potential study participants in future research projects.

I have been informed that personal data will be processed as part of this study. I am specifically aware of the purpose, scope, legal basis, and duration of data storage. In addition, I am aware of my rights regarding my personal data with respect to the responsible party.
I have received, read, and understood the corresponding written study information.

I have received, read and understood the corresponding written study information.

I am aware that participation is voluntary and that I can revoke it at any time, without providing a reason and without any personal disadvantage, either in writing or verbally. My data will then be completely deleted, and I will receive a notification about this.

I had enough time to think about participating in the study and make a decision.

I agree to participate in the above-mentioned study and consent to the processing of my personal data, as explained to me. Insofar as special personal data, as defined in Art. 9 DS-GVO (such as health data), is collected, my consent also extends to this information.

I have received a copy of this consent declaration.

Place and date	Surname and first name (in block letters) of the participating person	Signature of the participating person

I have informed the person participating in the study both orally and in writing about the objectives, duration, procedure, benefits and all risks of the study. All questions that arose were answered by me clearly and in sufficient detail. The participating person has given their consent voluntarily. I have provided the participating person with the written study information and a copy of this declaration of consent.

Place and date	Surname and first name (in block letters) of the person providing information	Signature of the person providing information

Page 6 of 6

Appendix 8a: Information and Consent Flyer for Patients (Part I and II)

Study Participation

Who can participate in the Study?

You can participate if you are an adult and have spent at least two nights in a regular hospital ward (not a monitoring or intensive care unit) at the time of data collection

What does participation involve?

- If you participate in the study, you will complete a questionnaire about your sleep before and during your hospital stay. You will also evaluate the written study materials and answer questions about yourself.
- The questionnaire takes about 15–20 minutes to complete.
- Once completed, place the questionnaire in the provided envelope. It will be collected one day after distribution.
- Information about your hospital stay will be recorded by a staff member from your ward.

What are the benefits and risks of the Study?

There is no direct benefit to you from participating. However, your contribution is valuable for achieving the study's research goals.

Participation involves minimal burden, but risks cannot be entirely ruled out. These risks are considered to be very low.

Data Protection

- The Data Protection Officers of the University of Cologne[1] are responsible in accordance with Art. 4 Para. 7 DS-GVO.
- If required, if needed, you can contact the study leader[2].
- You also have the right to file a complaint with a data protection authority[3].

Data processing, Usage and storage

- Personal data (including health-related data) will be collected, processed, and used on paper during the study.
- The use of your data requires your written consent.
- Data will initially be pseudonymised (i.e., no identifying information, only a code with letters and numbers). A list linking names to pseudonyms will be maintained and destroyed permanently after data collection. After this, the data will be anonymised (i.e., not traceable to any person).
- Anonymised data will be securely stored at the Institute of Nursing Science at the University of Cologne and protected against unauthorised access. The data will be deleted after ten years.
- During this time, the data will only be used for the purposes of scientific research based on your informed consent. It may be used for publications, future studies, or shared with research partners if necessary.

Right to withdraw and request removal

- Participation in the study is voluntary and can be ended at any time without giving a reason and without any disadvantage to you. You can notify your ward manager or the researchers verbally or in writing to withdraw.
- Fully anonymised data cannot be linked to any person and, therefore, cannot be deleted upon request.

Information about a Research Study by the Institute of Nursing Science at the University of Cologne

Sleep Acute

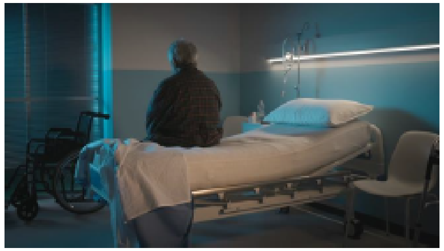

Survey of patients sleep during hospital inpatient care

Dear Patient,

The **Sleep Acute** study aims to learn more about sleep during a hospital stay. You are currently a patient and can support our research by participating **voluntarily**.

Information video

Scan the QR code or use the link to watch a video. The video explains the study's goals and procedures.

https://www.youtube.com/watch?v=TyRctoDIQ_o

In addition to the video, you can find all necessary information for study participation in this flyer.

If you have any unanswered questions, feel free to speak with your ward manager or contact us directly. Our contact details are at the end of the flyer.

Your Study Team

Background and Study Objectives

Sleep is a dynamic state that is vital for health, well-being, and daily functioning. Although illness often increases the need for rest and sleep, previous research show that sleep problems are common during hospital stays.

Our goal is to learn more about sleep and sleep problems in hospitalised patients. This will help identify needs and develop ways to improve care.

We are also analysing the acceptability and effectiveness of our written information materials. This will provide insights for designing materials for future studies.

Contact

[2]Study management

Prof. Dr phil. Sascha Köpke
Institute of Nursing Science, University of Cologne
Gleueler Straße 176-178
50935 Cologne
0221 478 51658
sascha.koepke@uk-koeln.de
https://pflegewissenschaft.uni-koeln.de

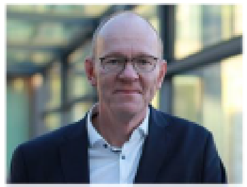

[1]Data Protection Officer, University of Cologne

Gesa Diekmann and Alexander May
Albertus-Magnus-Platz
50923 Cologne
0221 470 3872
dsb@verw.uni-koeln.de
https://verwaltung.uni-koeln.de/stabsstelle02.3/

[3]State Commissioner for Data Protection and Freedom of Information NRW

Helga Block
Kavalleriestraße 2-4
40213 Düsseldorf
0211 384240
poststelle@ldi.nrw.de
https://www.ldi.nrw.de

Consent for a research study by the Institute of Nursing Science at the University of Cologne

Sleep Acute

Survey of patients sleep during hospital inpatient care

Name of the participant:

____

Name of the person providing information:

____

I have received information about the nature, scope, and significance of the study. All my questions were answered satisfactorily.

The **Sleep Acute** study aims to learn more about sleep during inpatient care in hospitals. Based on this, needs are identified and approaches to improving the care situation are derived.

In addition, the acceptability and effectiveness of the study information materials used will be analysed. This serves to develop recommendations for recruiting potential study participants in future research projects.

I have been informed that personal data will be processed in the study. I understand the purpose, scope, legal basis, and duration of data storage. Furthermore, I am aware of my rights regarding the responsible entity.

I have received, read, and understood the corresponding written study information.

I am aware that participation in the study is voluntary and that I can withdraw at any time without providing reasons and without any personal disadvantage.

I agree to participate in the study and consent to the associated processing of my personal data. My consent also extends to special categories of personal data as defined in Art. 9 DS-GVO.

____

Place and date, signature of the participant

Appendix 8b: Script for the Information Video for Patients (Part I and II)

Information video for patients - Script

General conditions:

- Total duration: Approx. 2-5 minutes
- Speakers: Sascha Köpke and Marcelina Roos
- Recording location: Institute for Nursing Science at the University of Cologne

Contents:

What is behind the "Sleep Acute" study?

"Sleep Acute" is a nursing science study conducted by the Institute of Nursing Science at the University of Cologne. The primary aim of the study is to find out more about patients' sleep during an inpatient stay in hospital. We hope to be able to identify needs and derive approaches for improving the sleep situation from the knowledge gained.

At the same time, we are investigating another research question by analysing the acceptance and effectiveness of the written information materials we used in the sleep study. The aim is to obtain information on how study participants can be successfully recruited for research projects in the future."

Who can take part in the study and what exactly does participation look like?

"Patients can take part in the study if they are of legal age and have already spent at least two nights in hospital on a so-called normal ward, i.e. not a monitoring or intensive care unit, at the time of data collection.

Participation in the study involves completing two questionnaires. One of the questionnaires asks questions about the patient's sleep before and during hospitalisation. This allows us to compare sleep during hospitalisation with normal sleep. In the other questionnaire, patients are asked to evaluate the information material provided on the sleep study. Individual questions also relate to personal details, such as the age or gender of the participants. The questionnaires are expected to take 15 to 20 minutes to complete.

In order to minimise the duration of the survey for participating patients, further data relating to the hospital stay is collected via ward staff. This includes, for example, information on the day of admission, the admission diagnosis or the intake of sleep medication.

Why should patients take part in the study?

"The successful implementation of our study can only be realised with the support of patients. Participation will therefore make an important contribution to adequately describing the sleep situation of patients in hospital for the first time in Germany, identifying needs and deriving approaches to promoting sleep. The parallel investigation of the information materials also enables us to gain important insights into the design of the information materials in future scientific studies."

Conclusion

"Of course, your participation in the study is voluntary. You will find further information on the study in the flyer you have been given. If you have any questions, please speak to your ward manager or contact us. You will find our contact details on the back of the flyer."

Appendix 9: Questionnaire for Evaluating the Written Study Information

Study ID*: _____ _____ - _____ _____ - _____ _____ **Date**: _____._____.__________

*The study ID will be added by members of the study team after the questionnaire has been completed

Questionnaire for Evaluating the Written Study Information

Dear patient,

Thank you for participating in this survey. In this questionnaire, we kindly ask you to evaluate the information letter you received about the study. Providing as complete answers as possible will help us better understand your impressions.

Please answer the following questions by selecting the value on the response scales that applies to you.

1. How do you evaluate the fact that you were provided with written study information?

0	1	2	3	4	5
Very negative					Very positive

2. Did the written study information encourage you to participate in the study?

0	1	2	3	4	5
Not at all					Very much

3. How do you rate the written study information overall?

0	1	2	3	4	5
Very negative					Very positive

4. How do you evaluate the format and design of the written study information?

0	1	2	3	4	5
Very negative					Very positive

Page 1 of 4

5. How do you rate the written study information in terms of its clarity and understandability?

0 1 2 3 4 5

Not understandable at all — Very understandable

6. How do you evaluate the written study information in terms of its completeness?

0 1 2 3 4 5

Not complete — Complete

7. How do you evaluate the written study information in terms of its scope?

0 1 2 3 4 5

Not appropriate at all — Completely appropriate

8. How do you evaluate the content of the written study information in terms of its relevance?

0 1 2 3 4 5

Not relevant at all — Very relevant

Please write you're answers below each question.

9. What do you particularly like about the written study information?

10. What do you like less about the written study information?

11. What should be changed in the written study information?

Page 3 of 4

At the end of the questionnaire, we kindly ask you to provide the following information about yourself:

12. Please indicate your highest level of education.

- ☐ No school leaving certificate
- ☐ Lower secondary school certificate
- ☐ Intermediate school certificate
- ☐ University entrance qualification
- ☐ Completed vocational training
- ☐ (Specialised) university degree
- ☐ Doctorate

13. Please indicate your employment status.

- ☐ School student
- ☐ University student
- ☐ Apprentice
- ☐ Employed
- ☐ Self-employed
- ☐ Job-seeking/ unemployed
- ☐ Housewife or househusband
- ☐ Retired
- ☐ Other: ______________________________

14. Please specify your nationality.

____

15. Please specify your country of birth.

____

16. Please specify your native language.

____

Thank you for participating in the survey!

Page 4 of 4

Appendix 10: Information and Consent Letter for Healthcare Professionals (Part I)

This appendix provides additional details relevant to the host study (Part I).
