# Supplementary material for: Effects of a non-standard information leaflet on patient recruitment in acute care: Embedded cluster-randomised controlled trial
Source: PLoS One. 2025 Aug 1;20(8):e0327634. doi: 10.1371/journal.pone.0327634 (PMC12316219; doi:10.1371/journal.pone.0327634)
Supplement: Attachment — Submitted filename: Response to Reviewers.docx [file pone.0327634.s009.docx]

**PONE-D-25-02678**

**Effects of an information leaflet on patient recruitment for a survey in acute care: Embedded cluster-randomised controlled trial**

**PLOS ONE**

|  | **Journal Requirements** | **Response to Reviewers** |
| --- | --- | --- |
| 1. | When submitting your revision, we need you to address these additional requirements. Please ensure that your manuscript meets PLOS ONE's style requirements, including those for file naming. | Thank you for your guidance. We have reviewed the manuscript and all files, including file naming, to ensure full compliance with PLOS ONE’s style requirements. All necessary revisions have been made accordingly. |
| 2. | We note that you have selected “Clinical Trial” as your article type. PLOS ONE requires that all clinical trials are registered in an appropriate registry (the WHO list of approved registries is at https://www.who.int/clinical-trials-registry-platform/network/primary-registries" https://www.who.int/clinical-trials-registry-platform/network/primary-registries and more information on trial registration is at http://www.icmje.org/about-icmje/faqs/clinical-trials-registration/). Please state the name of the registry and the registration number (e.g. ISRCTN or ClinicalTrials.gov) in the submission data and on the title page of your manuscript.  a) Please provide the complete date range for participant recruitment and follow-up in the methods section of your manuscript.  b) If you have not yet registered your trial in an appropriate registry, we now require you to do so and will need confirmation of the trial registry number before we can pass your paper to the next stage of review. Please include in the Methods section of your paper your reasons for not registering this study before enrolment of participants started. Please confirm that all related trials are registered by stating: “The authors confirm that all ongoing and related trials for this drug/intervention are registered”. Please see http://journals.plos.org/plosone/s/submission-guidelines#loc-clinical-trials for our policies on clinical trials. | We registered our study in the German Clinical Trials Register (DRKS), which is included in the WHO list of approved registries. The name of the registry and the registration number are already stated on the title page of the manuscript, as required. p.2 Study registration: “DRKS-ID: DRKS00029707. Registered on 30. August 2022.”  a) We already reported the date range of participant recruitment (p.9): “Recruitment and data collection took place from 6 May 2021 to 29 June 2022.”  b) The study was registered retrospectively in August 2022 due to an unfortunate miscommunication within the study team, which is now addressed in the methods section (p.4): “The study registration was carried out retrospectively due to organisational reasons.” We also discuss this as limitation in the discussion section (p. 16): “Due to organisational reasons within the study team, the study was registered retrospectively. However, the study protocol had been approved by the ethics board before the study commenced. Also, as this is cross-sectional trial with a relatively simple study design, we do not consider this a major problem.” |
| 3. | In the online submission form, you indicated that [The data underlying the results presented in the study are available from the authors.].  All PLOS journals now require all data underlying the findings described in their manuscript to be freely available to other researchers, either 1. In a public repository, 2. Within the manuscript itself, or 3. Uploaded as supplementary information.  This policy applies to all data except where public deposition would breach compliance with the protocol approved by your research ethics board. If your data cannot be made publicly available for ethical or legal reasons (e.g., public availability would compromise patient privacy), please explain your reasons on resubmission and your exemption request will be escalated for approval. | Besides the supplements already submitted, we have now also provided our raw data as an SPSS file (p. 21: S6 File – SPSS row data), ensuring no personal data is disclosed. The data are provided as part of the manuscript and its supporting information. |
| 4. | Please review your reference list to ensure that it is complete and correct. If you have cited papers that have been retracted, please include the rationale for doing so in the manuscript text, or remove these references and replace them with relevant current references. Any changes to the reference list should be mentioned in the rebuttal letter that accompanies your revised manuscript. If you need to cite a retracted article, indicate the article’s retracted status in the References list and also include a citation and full reference for the retraction notice. | We have checked our list of references and confirm that everything is correct. |
| **Reviewer #1** | |  |
| 5. | The purpose of this research was to investigate the effectiveness and acceptability of an information leaflet including an information video compared to a formal information letter in patient recruitment in acute care.  Accordingly the investigators conducted a cluster-randomised controlled trial embedded in a cross sectional study (“Sleep Acute host study”) addressing patients’ sleep in hospitals. They allocated selected wards either to the intervention group (information leaflet including a link to an information video) or the control group (formal information letter) using external concealed randomization. The design was well thought out and the methodology was well described.  The primary endpoint was recruitment success measured by the participation rate in the Sleep Acute host study. The secondary endpoint was acceptability of the written study information. Persons involved in the analyses were blinded concerning group allocation.  Due to the study’s exploratory nature, there was no formal sample size calculation for the embedded cRCT, given that the sample size is constrained by the number of participants approached in the Sleep Acute host study, although a more rigorous hypothesized effect size would have been ideal . Also given the exploratory and mostly descriptive nature of the presentation it appears that the number per group (analyzed and participated) is adequate for the descriptive results reported.  Analysis was quantitative and qualitative. To analyze the primary endpoint ‘participation’, they used random effects logistic regression adjusted for cluster. Exactly what was the random effect or was it cluster? The odds ratios indicated no difference between the two groups. They also conducted bivariate linear regressions with the secondary endpoint . The explanation of the approaches was reasonable for this type of data. Qualitative data analysis involved the usual open-ended questions in a structured process. Comparison across questions is noted in Table 3. Differences of acceptability was not evident examining the confidence intervals of the slopes.  They do conclude that the use of an information leaflet does appear to improve participant recruitment. In terms of acceptability, they found some indications that study participants perceive certain elements of an information leaflet positively. Such was the mainly descriptive nature of the cautionary presentation. | We would like to thank you for your thoughtful review of our manuscript. |
| **Reviewer #2** | |  |
| 6. | Thank you for the opportunity to comment on this interesting, nested study (it is not a formal SWAT as the host study is not a trial). It is clearly described and the methods look rigorous, and is based on a large sample of wards and patients (although the sample was smaller than expected). I only had a few comments. The study is interesting as it is in a hospital context. | We would like to thank you for your thoughtful review of our manuscript.  You are right that the host study is not a clinical trial. We still chose to use the term SWAT as it is well-established and enhances the clarity and comprehensibility of our methodological approach. Although the host study is observational, the embedded study follows key SWAT principles. For this reason, we titled our study: Effects of a non-standard information leaflet on patient recruitment in acute care: Embedded cluster-randomised controlled trial. |
| 7. | The first line of the introduction seems to define ‘recruitment’ to include ‘retention’, whereas these are generally distinguished in the literature. | To avoid confusion, we decided not to include the aspect of retention in the introduction, as our study focuses solely on recruitment and does not involve follow-up/retention procedures (p.3): “Recruitment is defined as the identification of study participants, ensuring an adequate and/or representative sample [1].” |
| 8. | Can they clarify the consent procedures for the nested study? It is mentioned 3 times (page 4, 7 and 8). I think they just need to state in one place that people consented to the sleep survey, but not the embedded study. | Informed consent was obtained for participation in the host study (the sleep survey), but not specifically for the embedded study. For this reason, we deleted the sentence (p. 4, Methods - Study design) “All participants gave their written informed consent after ward randomisation” and specified it more clearly in the Methods - Outcomes and data collection section (p. 7): ”Patients received the written study information one day before data collection by a ward nurse. If ward nurses did not have enough time, the information was handed out on the day of data collection. The study team personally contacted all interested patients and provided information, answered questions, obtained patients’ written informed consent, and handed out and collected paper-based questionnaires for both the host study and the embedded cRCT. Of patients who declined to participate in the study, only their anonymously recorded decision was used for data analysis.” |
| 9. | On page 7, they suggest that extensive development is a basis for validity. I am not sure that is the consensus view and I do not think they need to make that claim. They just need to be clear what they did do for validity assessment, and what they did not do – and let readers judge the validity. | We did not conduct psychometric testing during the development of the questionnaire. Therefore, we deleted the sentence on page 7 “Even though there was no psychometric testing, the extensive development process let us assume that the questionnaire validly captures the acceptability” and specified it more clearly in the Discussion – Strengths and limitations section (p. 14-15): “A limitation of our study is the lack of psychometric evaluation of the questionnaire. Although we developed the instrument through an extensive process, we refrained from conducting a formal validation for pragmatic reasons. This should be addressed in any future use of the questionnaire.” |
| 10. | Can they clarify the proportion of patients who received the information one day before, and those who got it on the day (page 6)? I think that would be useful context. | Unfortunately, we do not have the data on the proportion of patients who received the information the day before and those who received it on the day of data collection, so we are unable to make valid statements regarding this distribution.  p. 16: “If ward nurses were unable to distribute written study information on the day before data collection due to time constraints, researcher provided it to the patients on the day of data collection. As a result, patients had varying amounts of time to review the study materials, which may have influenced their decision to participate [5]. Unfortunately, we are unable to provide valid data on the date of distribution of study information.” |
| 11. | I think it would be useful to comment on the baseline level of participation (which I think is 20% of eligible patients being approached at all, and around 50% of those approached taking part). | Sorry, this seems to be a misunderstanding. Of the n= 1,321 patients assessed for eligibility (n= 736 in the intervention group and n= 585 in the control group), approximately one-third (n= 498) were eligible and randomly assigned to the respective intervention (n= 262 in the intervention group and n= 236 in the control group). Of the eligible patients, 62% (n= 165) received the allocated intervention and 65% (n= 154) received the allocated control intervention.  For clarity, we have added “eligible for study participation” at the Allocation level in the flowchart (Fig 1). |
| 12. | Am I right in thinking that the ‘window’ for people to take part once asked was 24 hours (‘same or following day’)? That might have limited the potential impact of the intervention. | The time window for expressing willingness to participate varied between one and 24 hours. While this may have influenced participation, we are unable to determine the extent or direction of this effect.  p. 16: “If ward nurses were unable to distribute written study information on the day before data collection due to time constraints, researcher provided it to the patients on the day of data collection. As a result, patients had varying amounts of time to review the study materials, which may have influenced their decision to participate [5]. Unfortunately, we are unable to provide valid data on the date of distribution of study information.” |
| 13. | Do they know how many people visited the website through the QR code? I think that would be useful information to provide. If they do not have that (and I have been in that position), I would be explicit. | We recorded how often the information video was assessed via the QR code within the study period. Unfortunately, we cannot determine the number of people who accessed the information video as we cannot rule out multiple accesses by the same person, although we would not expect a significant number of multiple viewers. To clarify this, we have added the following on page 11 (Results – Acceptability): “During data collection, the information video was accessed through the QR code 15 times.” And on page 14 (Discussion – Strengths and imitations): “Another strength of our study is the inclusion of an informational video as part of the leaflet, accessible via QR code. This video offers an easy way to access study information without the need to read, potentially increasing accessibility and participation. However, the video was rarely accessed and we cannot rule out multiple access by the same person or access by persons outside the target group.” |
| 14. | I think it would help if the intervention was better labelled. The title says ‘information leaflet’ and does not mention the full intervention. I think many readers may assume that a ‘leaflet’ is pretty standard and it would be helpful to label the intervention in such a way that highlights its differences from ‘usual care’ (which I assume is length/detail, and the QR link).  Apologies for the self-citation but Madurasinghe et al. BMC Med. 2023 Nov 8;21(1):425. doi: 10.1186/s12916-023-03081-5 might be a relevant comparison as these studies involved links to multimedia. | We agree that a clearer labelling of the intervention will enhance understanding for the readers. In response to your suggestion, we have updated the title to: "Effects of a non-standard information leaflet on patient recruitment in acute care: Embedded cluster-randomised controlled trial."  Your results support and strengthen our discussion. We have therefore added the following sentence to our manuscript (p. 13): “A recent meta-analysis of SWATs supports this finding, indicating that the use of multimedia alongside written study information results in little or no difference in recruitment rates [24].” |
| 15. | Can they clarify the reason for the missing data in Table 1? Is it right that they did not have age and gender data for all patients? | Yes, you are correct — we do not have age and gender data for all patients. As noted in Table 1 (“*Data only available for participants in the host study”), these demographic data were only collected within the questionnaire of the host study. Initially, we assumed that this would be sufficient. However, it later became clear that this approach resulted in missing data for several patients: specifically, those who participated only in the Sleep-Recruit study, but not in the host study and those who were eligible but did not receive the allocated intervention or control materials, for reasons such as lack of interest, physical or emotional burden, lack of time, or other factors (Fig. 1 – Flowchart).  To clarify this, we have added the following on page 16 (Discussion – Strengths and limitations): “Another limitation of our study is the lack of age and gender data for all participants, as these were only collected in the host study and are missing for those in Sleep-Recruit only or for eligible individuals who did not receive the intervention.” |
| 16. | There is a statement in the discussion (page 15) which talk about ‘potential impact’ and the abstract says that the intervention group showed larger effects but that it was ‘not significant’. Formally this is true, but it is a particular framing – ‘no statistically significant effect of the intervention’ would be equally valid. I would go for the most neutral framing possible I think | Thank you for this thoughtful comment. We acknowledge your point regarding the framing of the results. However, we would prefer to retain our current wording, as it aligns with recent methodological recommendations that emphasize not focusing solely on statistical significance, but also considering effect sizes and confidence intervals. Stating only that there was “no statistically significant effect” would omit information about the direction and magnitude of the observed effects, which we believe are relevant for interpretation.  Schünemann HJ, Vist GE, Higgins JPT, Santesso N, Deeks JJ, Glasziou P, Akl EA, Guyatt GH. Chapter 15: Interpreting results and drawing conclusions [last updated August 2023]. In: Higgins JPT, Thomas J, Chandler J, Cumpston M, Li T, Page MJ, Welch VA (editors). Cochrane Handbook for Systematic Reviews of Interventions version 6.5. Cochrane, 2024. Available from [cochrane.org/handbook](https://training.cochrane.org/handbook)  Guyatt GH, Oxman AD, Kunz R, Brozek J, Alonso-Coello P, Rind D, et al. GRADE guidelines 6. Rating the quality of evidence--imprecision. J Clin Epidemiol. 2011 Dec;64(12):1283–93. |
